# A deep neural network-enhanced multi-scale coupled model of patient-specific acoustic wave propagation for mechanotransduction stimulation of healthy and osteoarthritic knee articular cartilage

**DOI:** 10.1007/s10237-026-02138-7

**Published:** 2026-10-17

**Authors:** Nuno A. T. C. Fernandes, Betina Hinckel, Ana leal, Óscar Carvalho

**Affiliations:** 1 Center for Micro-Electro Mechanical Systems (CMEMS), University of Minho, 4800-058 Guimarães, Portugal; 2 Department of Orthopaedic Surgery, William Beaumont Hospital, Royal Oak, MI 48067 USA; 3 Associate Laboratory in Biotechnology and Bioengineering and Microelectromechanical Systems (LABBELS), University of Minho, 4800-058 Guimarães, Portugal

**Keywords:** Ultrasound stimulation, Knee osteoarthritis, Chondrocyte, Multi-scale modelling, Deep neural network, Mechanotransduction, Wave propagation, Complex media, Linear and nonlinear acoustics

## Abstract

This study presents a multi-scale computational framework to quantify how externally applied acoustic waves propagate through the knee joint and induce mechanotransductive strain at the level of the chondrocyte nucleus. Patient-specific knee geometries (*n* = 8) were used to simulate acoustic wave transmission through layered tissues, while a representative volume element modelled the coupled solid–shell–acoustic behaviour of the chondrocyte, pericellular matrix, and extracellular matrix. Modal and frequency–response analyses identified the spheroidal *s*_3_ mode as the dominant contributor to nuclear deformation. Strain amplification was found to occur within defined frequency bands rather than at a single frequency, with healthy patients exhibiting a broader mid-frequency amplification region (50–170 kHz) and diseased patients showing a shifted low-frequency (30–85 kHz) dominance due to hypertrophy and altered stiffness. Macro-to-micro coupling demonstrated that, despite anatomical variability, consistent transmissibility trends were preserved across patients, with ideal frequencies for healthy and OA patients. Time-explicit simulations comparing linear and nonlinear acoustic formulations revealed negligible harmonic generation at the applied pressure levels, indicating that linear acoustics adequately describe propagation within the mechanotransductive strain range considered. Overall, the results demonstrate that cartilage stimulation is frequency-band dependent and disease-sensitive, and they provide a mechanistically grounded basis for optimizing ultrasound excitation parameters based on cellular-scale mechanics rather than empirical frequency selection.

## Introduction

Osteoarthritis (OA) is one of the most pressing public-health issues of our time: globally it affects over 500 million people (7% of the world population) and places massive strain on healthcare systems and societies (Leifer et al. 2022). Knee OA is a degenerative joint disease that develops when the articular cartilage and/or menisci progressively degenerate, leading to friction, inflammation, direct bone-to-bone contact within the knee joint and symptoms like pain, stiffness and reduced mobility. This process results in varying levels of pain and functional limitation, often impairing the patient’s ability to perform daily activities (). In terms of economic impact, OA’s annual cost (direct and indirect) in countries with market economies can amount to 1–2.5% of gross national product, with individual annual costs ranging from about US$700–US$15 600 (Leifer et al. 2022; Zhang et al. 1990). Despite this burden, therapeutic options remain mostly symptomatic and invasive (including joint replacement) and there is a distinct lack of truly non-invasive treatments that protect cartilage without accelerating degeneration (Fernandes et al. 2022). If demonstrated to be clinically effective, ultrasound stimulation could contribute to reducing this burden by preserving cartilage integrity, slowing disease progression, maintaining joint function, and potentially delaying the need for invasive procedures and long-term symptomatic treatment. However, the magnitude of this economic benefit remains uncertain and will require confirmation through clinical and health-economic studies.

Recent work has increasingly explored the use of ultrasound waves as a general biological stimulation therapy, and more specifically as a non-invasive mechanostimulation strategy for cartilage repair and OA. In vitro studies on both healthy and OA chondrocytes and cartilage explants have shown that ultrasound can enhance matrix synthesis (e.g., proteoglycan and type II collagen production), promote cell proliferation and migration, and suppress catabolic and inflammatory pathways (such as NF-*κ*B, JNK, p38) in OA cells while activating anabolic signalling (PI3K/Akt, ERK) in healthy cells (Uddin et al. 2016; Oliveira et al. 2023, 2022, 2025; Korstjens et al. 2008). These findings suggest that ultrasound may convert acoustic pressure waves into mechanical strain at the cell boundary and within the pericellular matrix, thereby triggering mechanotransduction in chondrocytes. However, while the biological evidence is promising, detailed mechanistic understanding (particularly of how the mechanical stimulus is transmitted through the ECM/PCM–cell system) and the optimal ultrasound parameters remain under investigation (Oliveira et al. 2023; Sekino et al. 2018).

According to vibration and acoustic wave propagation theory, each structure exhibits characteristic resonance frequencies that depend on its geometry, boundary conditions, and material properties (Rao xxxx). When the excitation frequency approaches one of these natural frequencies, energy transfer into the structure is significantly amplified, resulting in higher local deformation and stress fields. In the context of ultrasound stimulation of chondrocytes, identifying such resonance frequencies could enable maximization of mechanical energy delivery to the cell and its substructures (e.g., plasma membrane, cytoskeleton, and nucleus). This resonance-based optimization may enhance mechanotransduction efficiency by amplifying membrane tension, ion channel activation, and intracellular strain transmission, all of which are implicated in chondrocyte biosynthetic responses (Louw et al. 2013). Therefore, performing a parametric modal analysis of the chondrocyte– extracellular matrix (ECM) system provides a physically grounded approach to determine the optimal ultrasound frequency range for therapeutic stimulation.

Despite its potential, accurately modelling ultrasound propagation and resonance within biological tissues remains a significant challenge. The highly heterogeneous and anisotropic nature of cartilage, comprising the ECM, pericellular matrix (PCM), and chondrocytes, leads to complex scattering, reflection, and refraction phenomena that affect acoustic energy transmission. Moreover, the nonlinear viscoelastic behaviour of both the solid and fluid phases introduces amplitude-dependent responses and damping effects that attenuate wave propagation (Rudenko 2022; Peng 2021). These factors make it difficult to predict how mechanical energy is distributed at the cellular level, especially under realistic geometrical and boundary conditions. Consequently, achieving a reliable numerical representation requires the coupling of acoustic–structure interaction models with nonlinear material laws and frequency-dependent damping formulations to capture the true dynamic behaviour of cartilage under ultrasound excitation (Fernandes et al. 2025a).

The objective of this study is to develop a realistic computational framework to simulate and analyse ultrasound-induced wave propagation from the skin surface to the knee cartilage. To achieve physiological accuracy, patient-specific MRI data are used to reconstruct the geometry of the knee joint, allowing the model to capture individual anatomical variation. The mechanical and acoustic properties of the biological tissues are defined using previously characterized and experimentally validated datasets (Fernandes et al. 2025a), ensuring that the numerical model reflects the real mechanical behaviour of soft tissues. A multiscale modelling framework is adopted to couple the macroscopic propagation of ultrasound through layered joint tissues with the microscopic mechanical environment surrounding chondrocytes within the cartilage. At the microscale, representative volume elements (RVEs) composed of the extracellular matrix, pericellular matrix, and chondrocyte are used to investigate local mechanical responses. Within this context, a modal analysis in the frequency domain is performed to determine the natural resonance frequencies of chondrocytes, which are hypothesized to correspond to optimal ultrasound stimulation frequencies that maximize local strain and mechanotransductive efficiency. To evaluate the influence of physiological variability at the microscale, a sensitivity analysis was conducted in conjunction with surrogate modelling based on a deep neural network (DNN). The sensitivity analysis was used to quantify the contribution of the investigated geometrical and material parameters to the variability of the predicted mechanical response, thereby identifying the parameters with the greatest influence on the numerical outputs. The most relevant variables were subsequently incorporated into the surrogate model, which enabled the parameter space to be explored at a substantially higher resolution than would be computationally feasible using repeated finite-element simulations alone. This combined approach provided a more detailed assessment of the effects of biological variability while reducing the computational cost associated with extensive parametric analyses.

To fully characterize the dynamic behaviour of the system, simulations are conducted across three complementary regimes. In the frequency domain, harmonic analysis provides steady-state responses and resonance identification; in the linear transient domain, time-dependent responses to impulsive or chirped excitations are examined; and in the nonlinear time-explicit domain, viscoelasticity, damping, and nonlinear wave propagation are incorporated to capture the realistic behaviour of cartilage and surrounding tissues. This comprehensive, experimentally grounded, and multiscale approach enables the identification of frequency ranges and excitation conditions that enhance mechanical energy transfer to the chondrocytes, offering new insights into the optimization of non-invasive ultrasound therapies for osteoarthritis.

## Materials and methods

### Physics theory

The propagation of ultrasound or mechanical pressure waves in a fluid–structure system can be described in both the time-domain and frequency‐domain by the linear acoustic wave equation, and when amplitude or medium nonlinearity is non-negligible, by the Westervelt equation. In the linear (small‐amplitude) regime, the scalar acoustic pressure field p(x,t) in a homogeneous inviscid fluid satisfies Eq. (1)

1 $${\nabla }^{2}p-\frac{1}{{c}^{2}}\frac{{\partial }^{2}p}{\partial {t}^{2}}=0$$

where c is the speed of sound in the medium (Temkin 1981).

In the frequency domain, one writes $p\left(x,t\right)=\mathfrak{R}\{P\left(x,\omega \right){e}^{i\omega t}\}$, thus rewriting Eq. (1) into Eq. (2), also known as Helmholtz equation

2 $${\nabla }^{2}P+{k}^{2}P=0$$

where k is the wave number expressed as $k=\frac{\omega }{c}$. Both of these classical wave equations establish direct relationships among pressure, particle velocity, and acoustic impedance (density × sound speed) of the medium(Temkin 1981).

When sound propagation involves significant viscous, thermal or amplitude-dependent effects (for example in soft tissue or biological media), the Westervelt equation offers a more general model. The typical form in a lossless fluid is shown in Eq. (3)

3 $${\nabla }^{2}p-\frac{1}{{c}^{2}}\frac{{\partial }^{2}p}{\partial {t}^{2}}=\frac{\beta }{{\rho}_{0}{c}^{4}}\frac{{\partial }^{2}}{{\partial t}^{2}}({p}^{2})$$

where $\beta =1+\frac{B}{2A}$ is the nonlinearity coefficient (dimensionless), in which A and B are, respectively, the first- and second-order coefficients in the Taylor expansion of the equation of state relating pressure to density variations, often being represented too as B/A, the thermodynamic nonlinearity parameter of the medium, and ${\rho}_{0}$ the ambient density (kg/m^3^) (Blackstock xxxx; Hamilton and Blackstock 1988). When dissipation (sound diffusion) is included, a dissipative term enters Eq. (3), modifying it into Eq. (4)

4 $${\nabla }^{2}p-\frac{1}{{c}^{2}}\frac{{\partial }^{2}p}{\partial {t}^{2}}+\delta \frac{\partial }{\partial t}{\nabla }^{2}p=\frac{\beta }{{\rho}_{0}{c}^{4}}\frac{{\partial }^{2}}{{\partial t}^{2}}({p}^{2})$$

where δ is a sound-diffusivity parameter (m^2^/s) reflecting viscous and thermal attenuation (Blackstock xxxx; Hamilton and Blackstock 1988).

In a coupled solid–fluid system (e.g., a cell and its surrounding medium or between soft tissues and bones), wave propagation must respect continuity (normal stress) and normal particle velocity at the interface. As such, an Acoustic-Structure Interface (ASI) is created in the shared boundary where the particle velocity of the fluid is defined as Eq. (5)

5 $$\rho _{f} \frac{{\partial \overrightarrow {{v_{f} }} }}{{\partial t}} = - \nabla p,\nabla \cdot \overrightarrow {{v_{f} }} = - \frac{1}{{\rho _{f} c^{2} }}\frac{{\partial p}}{{\partial t}}$$

where $\overrightarrow{{\mathbf{v}}_{{\boldsymbol{f}}}}$ is the fluid particle velocity (m/s) and ${\rho}_{f}$ its density (kg/m^3^). For the solid domain, Eq. (6) is enforced

6 $$\rho _{s} \frac{{\partial ^{2} \overrightarrow {u} }}{{\partial t^{2} }} = - \nabla \cdot \sigma ,\sigma = \mathbb{C}:,\varepsilon = \frac{1}{2}\left[ {\nabla \overrightarrow {u} + (\nabla \overrightarrow {u} )^{T} } \right]$$

where $\overrightarrow{{\boldsymbol{u}}}$ is the displacement vector (m), ${\boldsymbol{\sigma}}$ is the second-order Cauchy stress tensor (Pa), ${\boldsymbol{\varepsilon}}$ is the second-order infinitesimal strain tensor (dimensionless), and ${\mathbb{C}}$ is the fourth-order elasticity tensor (Pa). The double contraction ${\mathbb{C}}:{\boldsymbol{\varepsilon}}$ defines the constitutive relation between strain and stress. As such, at the interface one enforces Eq. (7)

7 $$\overrightarrow {{v_{f} }} \cdot \overrightarrow {n} = \frac{{\partial \overrightarrow {u} }}{{\partial t}} \cdot \overrightarrow {{\mathbf{n}}} , - p\overrightarrow {n} = \sigma \cdot \overrightarrow {{\mathbf{n}}}$$

where $\overrightarrow{\mathbf{n}}$ is the outward normal from the fluid into the solid.

Furthermore, an acoustic wave does not propagate the same way in fluid and solid media. Due to the presence of longitudinal and transversal waves in solid media, Eq. (1) is modified into an equation that can account for both wave types, the Navier–Cauchy equation (Eq. (8))

8 $$\rho \frac{{\partial ^{2} \overrightarrow {{2u}} }}{{\partial t^{2} }} = \left( {\lambda + \mu _{{Lam\mathop e\limits }} } \right)\nabla \left( {\nabla \cdot \overrightarrow {{2u}} } \right) + \mu _{{Lam\mathop e\limits}} \nabla ^{2} \overrightarrow {{2u}}$$

which results from combining Hooke’s law for linear, isotropic elasticity with the balance of linear momentum. Here, $\mathrm{u}$ is the displacement field (m), ρ is the density (kg/m^3^), and λ and μ are the Lamé parameters (Pa) that characterize the material’s resistance to volumetric and shear deformation. The left-hand side represents inertial forces (mass times acceleration), while the right-hand side encapsulates the internal restoring forces produced by elastic stresses. The term $\left( {\lambda + \mu_{{{\mathrm{Lame}}}} } \right)\nabla \left( {\nabla \cdot u} \right)$ governs compressional or longitudinal waves, which arise from local changes in volume and travel at speed. $c_{p} = \sqrt {{{\left( {\lambda + 2\mu_{{{\mathrm{Lame}}}} } \right)} \mathord{\left/ {\vphantom {{\left( {\lambda + 2\mu_{{{\mathrm{Lame}}}} } \right)} \rho }} \right. \kern-0pt} \rho }} .$ The second term, $\mu_{{{\mathrm{Lame}}}} \nabla^{2} u,$ governs shear waves, driven by distortional strain, propagating at $c_{s} = \sqrt {{{\mu_{{{\mathrm{Lame}}}} } \mathord{\left/ {\vphantom {{\mu_{{{\mathrm{Lame}}}} } \rho }} \right. \kern-0pt} \rho }}$ However, the relevant outcome of this study is not the pressure field itself, but the strain induced within the chondrocyte and, critically, within its nucleus. Mechanical stimulation becomes biologically meaningful only when intracellular and intranuclear deformations exceed specific thresholds known to trigger mechanotransductive signalling. Multiple studies have quantified how much nuclear deformation is required to trigger mechanotransduction pathways. For instance, in the landmark study by Driscoll et al. found that mesenchymal stem cells subjected to a 3% global substrate stretch (1 Hz cyclic) exhibited a Lagrangian nuclear strain of approximately 5–10%, and it was clear only when the nucleus was appropriately deformed via the cytoskeleton that the mechanosensitive transcriptional co-activator YAP/TAZ mechanism translocated to the nucleus (Driscoll et al. 2015). More broadly, Kalukila et al. outlines in their review report that nuclear deformations in the range of 1–10% (and sometimes up to 30% for larger structural changes) suffice to alter chromatin conformation, nuclear pore transport dynamics and gene expression (Kalukula et al. 2022). Although the exact threshold varies with cell type, loading mode and duration, a working range is that 1–10% strain is biologically meaningful for mechanotransduction via nuclear deformation.

Therefore, the purpose of coupling the acoustic wave equations (Eqs. 1–4) with the elastic solid equations (Eqs. 6, 7) is to determine how an externally applied acoustic or mechanical wave propagating from the patient’s skin all the way through cartilage ultimately produces deformation at the nuclear scale. The fluid–solid interface conditions ensure that acoustic pressure in the ECM is transmitted into stresses within the cell body, while the Navier–Cauchy equation governs how these stresses generate compressional (P-wave) and shear (S-wave) deformation patterns inside the cytoplasm, PCM, and nucleus. By solving these equations in the frequency domain, one can quantify the frequency–response function relating applied pressure $p\left(\omega \right)$, thereby identifying the modal frequencies that most efficiently amplify nuclear deformation.

### Geometric and anatomical modelling

#### Articular cartilage geometry

**Fig. 1:**
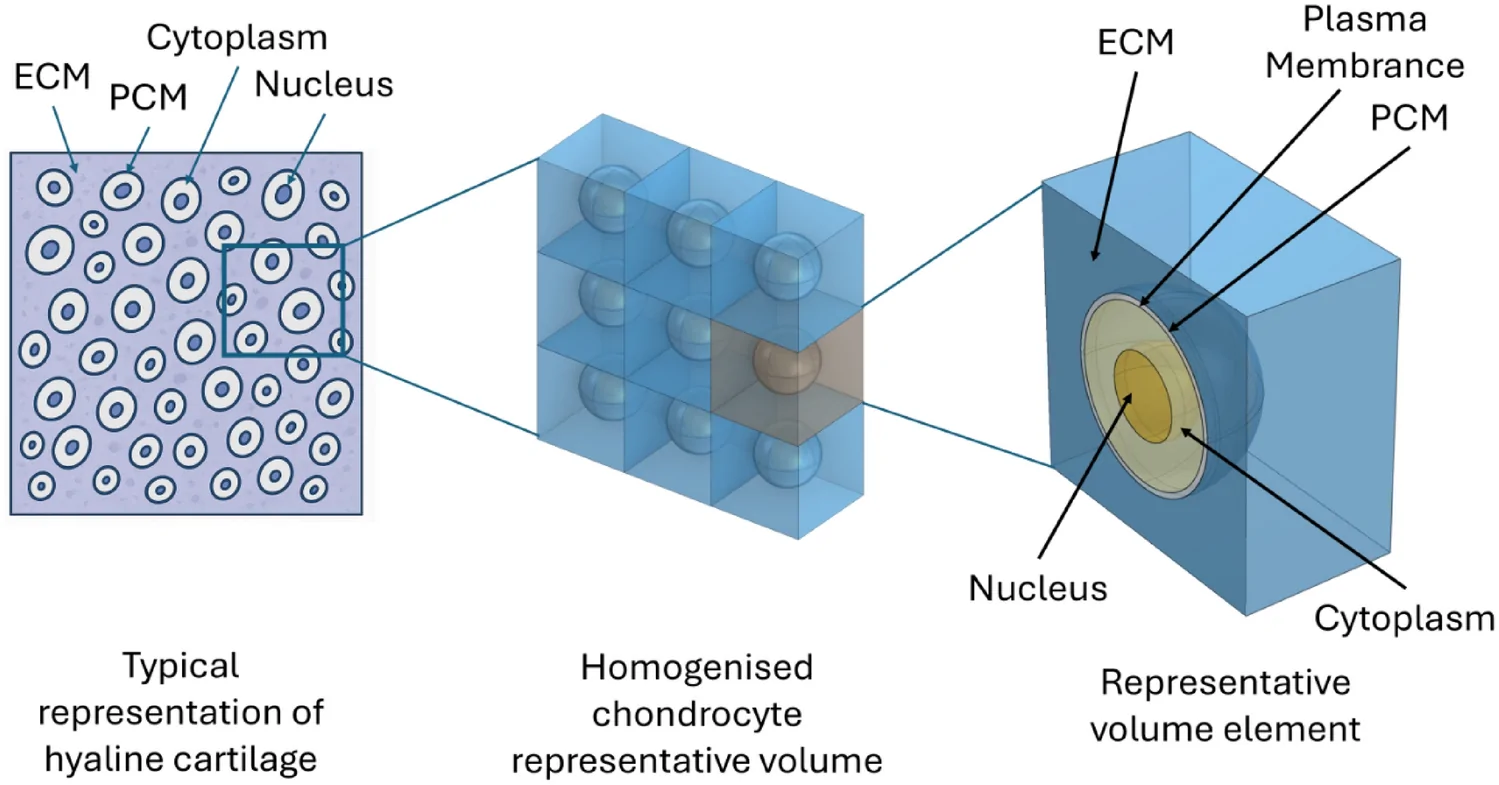
Schematic illustration of hyaline cartilage structure, showing ECM, PCM, and embedded chondrocytes. A representative chondrocyte volume is isolated and expanded to highlight nucleus, cytoplasm, plasma membrane, and surrounding PCM, forming the basis for the homogenized representative volume element used in modelling

In order to represent hyaline cartilage, a parametric homogenized representative volume element (RVE) was employed, as is displayed in Fig. 1. Within this framework, the size and distribution of the constituent components were varied parametrically to account for the natural heterogeneity observed in cartilage tissue. More specifically, a sensitivity analysis (Sect. 2.5) and a surrogate modelling strategy was employed to perform a parametric sweep (Sect. 2.6) over the identified intervals defined in Table 1, in order to study how chondrocyte radius, PCM thickness, plasma membrane thickness, and chondrocyte spacing influence the model. This approach allows for capturing the mechanical and structural variability of hyaline cartilage while maintaining a manageable computational model.

**Table 1:** Morphological parameters of chondrocytes and their pericellular environment in healthy and OA cartilage, including cell radius, nuclear radius, membrane and PCM thicknesses, and inter-cellular spacing, with corresponding literature references

Parameter	Values		Reference
	Healthy	OA	
Chondrocyte radius (${r}_{c}$–*µ*m)	5–10	10–20	Nahian and Sapra (2025); Chawla et al. (2022)
Nucleus radius (${r}_{n}$–*µ*m)	$\frac{{r}_{c}}{\sqrt[3]{2}}$	$\frac{{r}_{c}}{\sqrt[3]{2}}$	Nahian and Sapra (2025); Chawla et al. (2022); Chan and Marshall (2010); Pascarelli et al. (2015)
Plasma membrane thickness (${t}_{\mathrm{P}\mathrm{M}}$–*µ*m)	0.005–0.01	0.005–0.01	Campos Muñiz and Fernández Perrino (2023)
PCM thickness (${t}_{\mathrm{P}\mathrm{C}\mathrm{M}}$–*µ*m)	2–4	1–3	Guilak et al. (2018)
Distance between chondrocytes (${d}_{c}$–*µ*m)	40–80	50–100	Nahian and Sapra (2025); Sandell and Aigner (2001); Pettenuzzo et al. (2023)

To dimension the RVE, the dimensions used for each of the chondrocytes is displayed in Table 1.

In osteoarthritic tissue, the chondrocyte radius was assumed to increase as a consequence of hypertrophic changes (Chawla et al. 2022). The nucleus was taken to be approximately half the size of the cell (Chan and Marshall 2010), a proportionality reported in healthy cartilage and considered to remain consistent even under hypertrophy (Chawla et al. 2022). Although Pascarelli et al. reported enlarged and morphologically altered nuclei in osteoarthritic chondrocytes, they did not quantify nuclear and cytoplasmic volumes, and therefore do not establish preservation of the nuclear-to-cytoplasmic volume ratio (Pascarelli et al. 2015). Because no reliable measurements of plasma-membrane thickness in chondrocytes (particularly in OA patients) were identified in the literature, a standard reference value for mammalian cells was adopted (Campos Muñiz and Fernández Perrino 2023). The PCM thickness, however, was assumed to decrease in OA due to the well-documented structural degradation of this matrix (Guilak et al. 2018). Regarding chondrocyte spacing, healthy adult hyaline cartilage typically exhibits intercellular distances on the order of tens of micrometres, commonly between 40 and 80 *µ*m depending on the depth zone (Nahian and Sapra 2025). In OA tissue, this spacing becomes more variable: some regions exhibit increased separation due to cell loss, whereas others form clusters (chondrons) in which cells are positioned closer together, leading to a broader estimated range of 50–100 *µ*m (Sandell and Aigner 2001; Pettenuzzo et al. 2023).

Chondrocyte spacing and spatial periodicity were incorporated into the model, with their implementation detailed in Sect. 2.3, “Mesh and Boundary Conditions.”

#### Patients’ data for anatomic knee models

A cohort that consisted of eight individuals available at the OpenKnee project database was used (Chokhandre et al. 2021, 2023), whose demographic and anthropometric information is summarized in Table 2.

**Table 2:** Demographic and anthropometric characteristics of the subjects included in the study. These parameters were incorporated into the computational workflow to ensure physiologically representative joint geometry

Patient	Cartilage damage	Knee	Age	Gender	Height (m)	Weight (Kg)	BMI
A	Medial patella—grade 2	Right Knee	71	Male	1.83	77.1	23.1
B	Medial central femur—grade 1 Medial trochlea—grade 1 Lateral patella—grade 2	Right Knee	67	Female	1.55	45.3	18.9
C	Grade 0	Left Knee	25	Female	1.73	68	22.8
D	Grade 0	Right Knee	46	Female	1.575	54.43	21.9
E	Posterior medial tibia—grade 1 Medial patella—grade 3	Right Knee	71	Female	1.524	49.4	21.3
F	Lateral posterior femur—grade 1 Medial trochlea—grade 1 Medial/lateral patella—grade 2	Right Knee	71	Male	1.8	65.77	22.7
G	Grade 0	Right Knee	40	Male	1.778	63.5	20.09
H	Lateral posterior femur—grade 1	Left Knee	34	Male	1.8	68.3	20

MRI data in the coronal plane were used to reconstruct the 2D macroscale knee models employed in the acoustic simulations. The MRI datasets were obtained from the Open Knee(s)/3DTechniques database, which provides specimen-specific knee imaging data suitable for anatomical reconstruction and computational modelling. The imaging protocol included a 3D T1-weighted sequence without fat suppression for general anatomical reconstruction (TR = 20 ms, TE = 6 ms; spatial resolution: 0.5 mm × 0.5 mm × 0.5 mm). Cartilage imaging was based on a 3D T1-weighted sequence with fat suppression (TR = 29 ms, TE = 5.3 ms; spatial resolution: 0.35 mm × 0.35 mm × 0.7 mm). Connective tissues were imaged using three orthogonal acquisitions in the axial, sagittal, and coronal planes, based on a proton-density sequence (TR = 10,000 ms, TE = 9.7 ms; spatial resolution: 0.35 mm × 0.35 mm × 2.8 mm) (Chokhandre et al. 2023).

For the 2D macroscale model, the coronal slice was selected to represent the central load-bearing tibiofemoral region, where the femoral condyles, tibial plateau, articular cartilage, menisci, and surrounding soft tissues could be clearly identified. This selection allowed the model to retain relevant subject-specific anatomical features, including cartilage thickness, joint geometry, limb alignment, and soft-tissue distribution, while maintaining a computationally tractable domain for acoustic propagation analysis.

The anatomical regions visible in the selected coronal slice were segmented and discretized into separate computational domains corresponding to the principal tissue components. For the 3D macroscale model, several voxel-threshold-based segmentation procedures were performed in 3D Slicer using the available MRI images to isolate the desired anatomical structures. The segmented volumes were then exported as surface geometries and imported into Blender, where smoothing, surface correction, and Boolean operations were performed to improve geometric continuity. The processed geometries were subsequently imported and rebuilt in COMSOL Multiphysics. Geometry-cleaning tools were applied to remove nonconforming features, correct surface inconsistencies, and ensure mesh-domain conformity before discretization.

### Material properties

#### Articular cartilage material properties

The values exposed in Table 3 summarize the mechanical and acoustic material properties adopted for the chondrocyte, nucleus, plasma membrane, PCM and ECM in both healthy and OA conditions.

**Table 3:** Mechanical and acoustic material properties used for the healthy and OA chondrocyte microenvironment

Parameter	Value				Reference
	Healthy		OA		
*Chondrocyte (cytoplasm)*					
Density (${\rho}_{\mathrm{C}\mathrm{y}\mathrm{t}\mathrm{o}\mathrm{p}\mathrm{l}\mathrm{a}\mathrm{s}\mathrm{m}}$–g/cm^3^)	1.04		0.65		Rohrbach et al. (2017); Bush and Hall (2003)
Young’s modulus (${E}_{\mathrm{C}\mathrm{y}\mathrm{t}\mathrm{o}\mathrm{p}\mathrm{l}\mathrm{a}\mathrm{s}\mathrm{m}}$–kPa)	8.9		4.0		Haudenschild et al. (2011)
Poisson’s ratio (${\nu}_{\mathrm{C}\mathrm{y}\mathrm{t}\mathrm{o}\mathrm{p}\mathrm{l}\mathrm{a}\mathrm{s}\mathrm{m}}$)	0.38		0.36		Trickey et al. (2006)
Damping ratio (${\zeta}_{\mathrm{c}\mathrm{y}\mathrm{t}\mathrm{o}\mathrm{p}\mathrm{l}\mathrm{a}\mathrm{s}\mathrm{m}}$)	$\zeta =\frac{\alpha \bullet c}{2\pi \bullet f}$		$\zeta =\frac{\alpha \bullet c}{2\pi \bullet f}$		Rohrbach et al. (2017)
*Nucleus*					
Density (${\rho}_{\mathrm{n}\mathrm{u}\mathrm{c}\mathrm{l}\mathrm{e}\mathrm{u}\mathrm{s}}$–g/cm^3^)	$0.99{\rho}_{\mathrm{C}\mathrm{y}\mathrm{t}\mathrm{o}\mathrm{p}\mathrm{l}\mathrm{a}\mathrm{s}\mathrm{m}}$		$0.99{\rho}_{\mathrm{C}\mathrm{y}\mathrm{t}\mathrm{o}\mathrm{p}\mathrm{l}\mathrm{a}\mathrm{s}\mathrm{m}}$		Dahl et al. (2008); Schlüßler et al. (2022)
Young’s modulus (${E}_{\mathrm{n}\mathrm{u}\mathrm{c}\mathrm{l}\mathrm{e}\mathrm{u}\mathrm{s}}$–kPa)	${1.4 E}_{\mathrm{C}\mathrm{y}\mathrm{t}\mathrm{o}\mathrm{p}\mathrm{l}\mathrm{a}\mathrm{s}\mathrm{m}}$		${2.0 E}_{\mathrm{C}\mathrm{y}\mathrm{t}\mathrm{o}\mathrm{p}\mathrm{l}\mathrm{a}\mathrm{s}\mathrm{m}}$		Ofek et al. (2009); Attur et al. (2012)
Poisson’s ratio (${\nu}_{\mathrm{n}\mathrm{u}\mathrm{c}\mathrm{l}\mathrm{e}\mathrm{u}\mathrm{s}})$	0.42		0.42		Leipzig and Athanasiou (2008)
Damping ratio (${\zeta}_{\mathrm{n}\mathrm{u}\mathrm{c}\mathrm{l}\mathrm{e}\mathrm{u}\mathrm{s}}$)	$0.5 {\zeta}_{\mathrm{c}\mathrm{y}\mathrm{t}\mathrm{o}\mathrm{p}\mathrm{l}\mathrm{a}\mathrm{s}\mathrm{m}}$		$0.5 {\zeta}_{\mathrm{c}\mathrm{y}\mathrm{t}\mathrm{o}\mathrm{p}\mathrm{l}\mathrm{a}\mathrm{s}\mathrm{m}}$		Guilak et al. (2000)
Plasma membrane					
Density (${\rho}_{\mathrm{P}\mathrm{M}}$–g/cm^3^)	1.14		1.14		McKeel and Jarett (1970)
Young’s Modulus (${E}_{\mathrm{P}\mathrm{M}}$–kPa)	$\frac{0.5}{{t}_{\mathrm{P}\mathrm{M}}}$		$\frac{0.5}{{t}_{\mathrm{P}\mathrm{M}}}$		Doktorova et al. (2019)
Poisson’s ratio (${\nu}_{\mathrm{P}\mathrm{M}})$	0.47		0.47		Trickey et al. (2006); Dahl et al. (2008)
*PCM*					
Density (${\rho}_{\mathrm{P}\mathrm{C}\mathrm{M}}$–g/cm^3^)	1.03		1.02		Julkunen et al. (2009); Wilusz et al. (2013)
Young’s modulus (${E}_{\mathrm{P}\mathrm{C}\mathrm{M}}$–kPa)	38.7		23.5		Alexopoulos et al. (2003); Alexopoulos et al. (2005)
Poisson’s ratio (${\nu}_{\mathrm{P}\mathrm{C}\mathrm{M}})$	0.044		0.03		Alexopoulos et al. (2003); Alexopoulos et al. (2005)
Damping ratio (${\zeta}_{\mathrm{P}\mathrm{C}\mathrm{M}}$)	0.05		0.05		Nguyen et al. (2010)
ECM					
Density (${\rho}_{\mathrm{E}\mathrm{C}\mathrm{M}}$–g/cm^3^)	1.10		1.08		Rohrbach et al. (2017); Nieminen et al. (2004)
Speed of sound (${c}_{\mathrm{E}\mathrm{C}\mathrm{M}}$–m/s)	1590		1558		Rohrbach et al. (2017); Joiner et al. (2001)
Acoustic attenuation coefficient (${\alpha}_{\mathrm{E}\mathrm{C}\mathrm{M}, 45 \mathrm{M}\mathrm{H}\mathrm{z}}$–dB/mm)	10.65		13.3		Rohrbach et al. (2017); Joiner et al. (2001)

The material parameters of the cellular domains were adapted according to current evidence on osteoarthritic alterations. For healthy chondrocytes, the cytoplasmic density was adopted from Rohrbach et al. (Rohrbach et al. 2017). For osteoarthritic chondrocytes, the density was estimated to be approximately 62% of the healthy value, based on the alterations reported by Bush and Hall (Bush and Hall 2003). The Young’s modulus values for the healthy and OA cytoplasm were obtained from Haudenschild et al. (Haudenschild et al. 2011), while the Poisson’s ratios were taken directly from experimental measurements reported in the literature (Trickey et al. 2006). The cytoplasmic damping ratio was calculated by converting the dissipative parameter measured by Rohrbach et al. into an equivalent damping ratio (Rohrbach et al. 2017).

Because direct measurements of chondrocyte nuclear density are not available, the nuclear density was assumed to be approximately 99% of the corresponding cytoplasmic density, meaning that the nucleus was considered approximately 1% less dense than the cytoplasm. The same relative density relationship was applied to healthy and OA chondrocytes because no evidence currently supports an OA-specific alteration in the nuclear-to-cytoplasmic density ratio (Dahl et al. 2008; Schlüßler et al. 2022).

The nuclear Young’s modulus was defined relative to the cytoplasmic modulus. Based on the in situ nuclear deformation analysis reported by Ofek et al., the nucleus was considered to be approximately 1.4–2.0 times stiffer than the surrounding cytoplasm (Ofek et al. 2009). A factor of 1.4 was adopted for the healthy nucleus, while a factor of 2.0 was considered for the OA nucleus to represent a possible increase in relative nuclear stiffness associated with OA-related changes in nuclear structure. This assumption is supported qualitatively by reports of increased lamin A expression and chromatin condensation in OA chondrocytes, both of which may contribute to greater nuclear mechanical resistance. However, because no direct measurements of the Young’s modulus of OA chondrocyte nuclei are currently available, the higher OA stiffness ratio should be interpreted as an evidence-informed modelling assumption rather than a directly measured material property (Attur et al. 2012).

A Poisson’s ratio of 0.42 was assigned to the nucleus in both healthy and OA conditions, based on experimentally determined values for chondrocyte nuclei. The same value was retained for both conditions because the available literature does not demonstrate a significant OA-related change in nuclear compressibility (Leipzig and Athanasiou 2008).

Direct measurements of nuclear dissipation at ultrasonic frequencies are also unavailable. Therefore, the nuclear damping ratio was estimated from the relationship between the loss factor and damping ratio for a lightly damped viscoelastic material. For a Kelvin–Voigt-type interpretation, in a lightly damped system the loss factor (ξ) may be approximated by Eq. (9),

9 $$\zeta \cong \frac{\eta }{2}=\frac{\omega \mu }{E2}$$

where ω is the angular frequency, ${\mu}_{\mathrm{v}\mathrm{i}\mathrm{s}\mathrm{c}}$ is the apparent viscosity and E is the Young’s modulus. Assuming that the cytoplasm and nucleus are subjected to the same excitation frequency, the ratio between their damping ratios may therefore be expressed as Eq. (10).

10 $${\zeta}_{\mathrm{n}\mathrm{u}\mathrm{c}\mathrm{l}\mathrm{e}\mathrm{u}\mathrm{s}}\cong {\zeta}_{\mathrm{c}\mathrm{y}\mathrm{t}\mathrm{o}\mathrm{p}\mathrm{l}\mathrm{a}\mathrm{s}\mathrm{m}}\frac{{\mu}_{\mathrm{v}\mathrm{i}\mathrm{s}\mathrm{c}, \mathrm{n}\mathrm{u}\mathrm{c}\mathrm{l}\mathrm{e}\mathrm{u}\mathrm{s}}}{{\mu}_{\mathrm{v}\mathrm{i}\mathrm{s}\mathrm{c}, \mathrm{c}\mathrm{y}\mathrm{t}\mathrm{o}\mathrm{p}\mathrm{l}\mathrm{a}\mathrm{s}\mathrm{m}}}\frac{{E}_{\mathrm{c}\mathrm{y}\mathrm{t}\mathrm{o}\mathrm{p}\mathrm{l}\mathrm{a}\mathrm{s}\mathrm{m}}}{{E}_{\mathrm{n}\mathrm{u}\mathrm{c}\mathrm{l}\mathrm{e}\mathrm{u}\mathrm{s}}}$$

Based on the nucleus-to-cytoplasm viscosity and stiffness ratios reported in the literature, this relationship produces an estimated nuclear-to-cytoplasmic damping ratio of approximately 0.5 (Guilak et al. 2000). The nuclear damping ratio was consequently defined as.

$0.5 {\zeta}_{\mathrm{c}\mathrm{y}\mathrm{t}\mathrm{o}\mathrm{p}\mathrm{l}\mathrm{a}\mathrm{s}\mathrm{m}}$ for both healthy and OA chondrocytes. This relationship preserves consistency between the assumed elastic and viscous properties of the two intracellular domains while avoiding the introduction of an independent and unsupported nuclear damping parameter. Nevertheless, because the available viscoelastic measurements were obtained under quasi-static or low-frequency loading, the resulting damping ratios should be regarded as effective modelling parameters rather than direct measurements at ultrasonic frequencies.

For the plasma membrane, a density of 1.14 g/cm3 was adopted based on the experimental measurements reported by McKeel et al. (McKeel and Jarett 1970). The Young’s modulus was defined as $\frac{0.5}{{t}_{\mathrm{P}\mathrm{M}}}$ kPa, following the stiffness relationship described by Doktorova et al. (Doktorova et al. 2019), where ${t}_{PM}$ represents the plasma membrane thickness. A Poisson’s ratio of 0.47 was selected based on the experimental findings of Trickey et al. (Trickey et al. 2006) and on the mechanotransduction-related considerations discussed by Dahl et al. (Dahl et al. 2008).

To date, direct measurements comparing the density, Young’s modulus, and Poisson’s ratio of isolated plasma membranes from healthy and osteoarthritic chondrocytes have not been reported. Although osteoarthritic chondrocytes have been associated with increased membrane cholesterol content and reduced membrane fluidity, these alterations cannot be directly translated into quantitative changes in elastic modulus, density, or Poisson’s ratio. In particular, membrane fluidity is not mechanically equivalent to stiffness, and the influence of cholesterol on membrane elasticity depends on the composition and organization of the surrounding lipid bilayer (Arai et al. 2019). Consequently, the same plasma membrane properties were assigned to the healthy and osteoarthritic conditions.

Based on the compositional characteristics reported by Julkunen et al. (Julkunen et al. 2009), the density of the pericellular matrix was assumed to be close to that of water. Osteoarthritis is associated with marked PCM softening, structural deterioration, expansion of the pericellular region, and increased tissue hydration. To represent these alterations, the effective solid fraction was assumed to decrease to approximately 6%, resulting in an estimated OA PCM density of 1.02 g/cm3 (Wilusz et al. 2013). The Young’s modulus and Poisson’s ratio assigned to the PCM were obtained directly from the experimental measurements reported by Alexopoulos et al. (Alexopoulos et al. 2003, 2005).

Because reliable experimental measurements of PCM energy dissipation at ultrasonic frequencies are not currently available, the viscoelastic parameters were defined using the generalized Maxwell formulation adopted by Nguyen et al. (Nguyen et al. 2010). The corresponding deviatoric viscosity was derived from an assumed mechanical loss factor evaluated at the operating frequency. In the absence of evidence supporting an OA-specific modification of this parameter, the same viscoelastic properties were assigned to the healthy and osteoarthritic PCM.

The ECM surrounding the chondrocyte–PCM complex was represented as a continuous, homogeneous acoustic medium. The material properties assigned to the healthy microenvironment were obtained directly from the experimental study conducted by Rohrbach et al. (Rohrbach et al. 2017). Under osteoarthritic conditions, the ECM density was reduced to 1.08 g/cm3 to account for collagen network disruption, progressive matrix loss, and increased tissue hydration (Nieminen et al. 2004), thereby shifting the effective density towards that of water. In addition, based on the findings of Joiner et al. (Joiner et al. 2001), the speed of sound was decreased by 2%, while the frequency-dependent acoustic attenuation was increased by 25% to represent OA-associated matrix degeneration.

#### Biological tissues material properties

Table 3 summarizes the acoustic properties assigned to the soft and hard tissues composing the macro-scale knee model, including skin, adipose tissue, skeletal muscle, bone, cartilage, and tendon (Table 4).

**Table 4:** Acoustic and mechanical properties of the biological tissues included in the macro-scale knee model

Parameter	Value	Reference
*Skin*		
Density (${\rho}_{\mathrm{s}\mathrm{k}\mathrm{i}\mathrm{n}}$–g/cm^3^)	1.13124	Fernandes et al. (2025a)
Speed of sound (${c}_{\mathrm{s}\mathrm{k}\mathrm{i}\mathrm{n}}$–m/s)	1654.93	Fernandes et al. (2025a)
B/A nonlinearity parameter	7.237	Fernandes et al. (2025a)
Sound diffusivity (${\delta}_{\mathrm{s}\mathrm{k}\mathrm{i}\mathrm{n}}$–m^2^/s)	$0.0026\cdot {f}^{-0.934}$	Fernandes et al. (2025a)
*Adipose tissue*		
Density (${\rho}_{\mathrm{a}\mathrm{d}\mathrm{i}\mathrm{p}\mathrm{o}\mathrm{s}\mathrm{e} \mathrm{t}\mathrm{i}\mathrm{s}\mathrm{s}\mathrm{u}\mathrm{e}}$–g/cm^3^)	0.927091	Fernandes et al. (2025a)
Speed of sound (${c}_{\mathrm{a}\mathrm{d}\mathrm{i}\mathrm{p}\mathrm{o}\mathrm{s}\mathrm{e} \mathrm{t}\mathrm{i}\mathrm{s}\mathrm{s}\mathrm{u}\mathrm{e}}$–m/s)	1461.77	Fernandes et al. (2025a)
B/A nonlinearity parameter	10.754	Fernandes et al. (2025a)
Sound Diffusivity (${\delta}_{\mathrm{a}\mathrm{d}\mathrm{i}\mathrm{p}\mathrm{o}\mathrm{s}\mathrm{e} \mathrm{t}\mathrm{i}\mathrm{s}\mathrm{s}\mathrm{u}\mathrm{e}}$–m^2^/s)	$0.0012\cdot {f}^{-0.608}$	Fernandes et al. (2025a)
*Skeletal muscle*		
Density (${\rho}_{\mathrm{s}\mathrm{k}\mathrm{e}\mathrm{l}\mathrm{e}\mathrm{t}\mathrm{a}\mathrm{l} \mathrm{m}\mathrm{u}\mathrm{s}\mathrm{c}\mathrm{l}\mathrm{e}}$–g/cm^3^)	1.05775	Fernandes et al. (2025a)
Speed of sound (${c}_{\mathrm{s}\mathrm{k}\mathrm{e}\mathrm{l}\mathrm{e}\mathrm{t}\mathrm{a}\mathrm{l} \mathrm{m}\mathrm{u}\mathrm{s}\mathrm{c}\mathrm{l}\mathrm{e}}$–m/s)	1562.18	Fernandes et al. (2025a)
B/A nonlinearity parameter	6.632	Fernandes et al. (2025a)
Sound diffusivity (${\delta}_{\mathrm{s}\mathrm{k}\mathrm{e}\mathrm{l}\mathrm{e}\mathrm{t}\mathrm{a}\mathrm{l} \mathrm{m}\mathrm{u}\mathrm{s}\mathrm{c}\mathrm{l}\mathrm{e}}$–m^2^/s)	$0.0024\cdot {f}^{-0.349}$	Fernandes et al. (2025a)
*Bone*		
Density (${\rho}_{\mathrm{B}\mathrm{o}\mathrm{n}\mathrm{e}}$–g/cm^3^)	1.86170	Fernandes et al. (2025a)
Longitudinal wave speed (${c}_{l, \mathrm{B}\mathrm{o}\mathrm{n}\mathrm{e}}$–m/s)	3193.95	Fernandes et al. (2025a)
Shear wave speed (${c}_{s, \mathrm{B}\mathrm{o}\mathrm{n}\mathrm{e}}$–m/s)	1500.00	White et al. (2006)
Sound diffusivity (${\delta}_{\mathrm{B}\mathrm{o}\mathrm{n}\mathrm{e}}$–m^2^/s)	$0.0024\cdot {f}^{-0.349}$	Fernandes et al. (2025a)
*Cartilage*		
Density (${\rho}_{\mathrm{C}\mathrm{a}\mathrm{r}\mathrm{t}\mathrm{i}\mathrm{l}\mathrm{a}\mathrm{g}\mathrm{e}}$–g/cm^3^)	1.03345	Fernandes et al. (2025a)
Speed of sound (${c}_{\mathrm{c}\mathrm{a}\mathrm{r}\mathrm{t}\mathrm{i}\mathrm{l}\mathrm{a}\mathrm{g}\mathrm{e}}$–m/s)	1650.80	Fernandes et al. (2025a)
B/A nonlinearity parameter	8.118	Fernandes et al. (2025a)
Sound diffusivity (${\delta}_{\mathrm{C}\mathrm{a}\mathrm{r}\mathrm{t}\mathrm{i}\mathrm{l}\mathrm{a}\mathrm{g}\mathrm{e}}$–m^2^/s)	$0.0261\cdot {f}^{-1.654}$	Fernandes et al. (2025a)
*Tendon*		
Density (${\rho}_{\mathrm{T}\mathrm{e}\mathrm{n}\mathrm{d}\mathrm{o}\mathrm{n}}$–g/cm^3^)	1.12106	Fernandes et al. (2025a)
Speed of sound (${c}_{\mathrm{T}\mathrm{e}\mathrm{n}\mathrm{d}\mathrm{o}\mathrm{n}}$–m/s)	1827.43	Fernandes et al. (2025a)
B/A nonlinearity parameter	7.047	Fernandes et al. (2025a)
Sound diffusivity (${\delta}_{\mathrm{T}\mathrm{e}\mathrm{n}\mathrm{d}\mathrm{o}\mathrm{n}}$–m^2^/s)	$0.0168\cdot {f}^{-0.801}$	Fernandes et al. (2025a)

For fluid-like soft tissues, wave propagation was described using density, speed of sound, nonlinearity parameter (B/A), and a frequency-dependent sound diffusivity to account for viscous and thermal attenuation. The sound-diffusivity values adopted in the present simulations were obtained from previously published experimental attenuation measurements performed on biological tissue samples over an ultrasonic frequency range. Their derivation and calibration were therefore not repeated in the present study, and the previously established tissue-specific parameters were directly implemented in the numerical mode (Fernandes et al. 2025a). In contrast, bone was modelled as a solid elastic medium, requiring separate longitudinal and shear wave speeds rather than a single acoustic velocity, reflecting its ability to support both compressional and transverse elastic waves. Although the selected tissue properties were derived from porcine and bovine models (Fernandes et al. 2025a), previous studies indicate that the acoustic impedance of animal soft tissues, typically ranging from 1.33 to 1.71 MRayl (Koch et al. 2011; Liu 2006) have got very similar values to that is comparable to the values reported for human soft tissues, which range from 1.33 to 1.72 MRayl (Chen et al. 2022). For cortical bone, the calculated acoustic impedance was 5.9 MRayl, which lies within the commonly reported range of 3.5–6.0 MRayl (Raum et al. 2002). Bone structures were represented as homogeneous effective domains in the present macroscale model. This simplification was adopted because the available MRI data did not provide sufficient anatomical contrast to reliably distinguish cortical and trabecular bone layers throughout the knee geometry. Although cortical and trabecular bone have different acoustic properties, at the investigated frequencies the acoustic wavelength is considerably larger than the characteristic trabecular microarchitecture and local cortical-layer thickness. Therefore, bone is expected to act primarily as a high-impedance reflecting boundary relative to the surrounding soft tissues, with the dominant contribution to the macroscopic pressure field arising from the bone–soft tissue impedance contrast and global joint geometry. Nevertheless, this approximation may influence the local magnitude of reflected waves and the detailed spatial distribution of pressure near bone interfaces. Future work should investigate multilayer bone representations using imaging modalities capable of resolving cortical and trabecular compartments, such as CT or high-resolution MRI.

### Mesh and boundary conditions

#### Representative volume element meshing

The RVE was constructed to capture the coupled mechanical and acoustic behaviour of an individual chondrocyte embedded within its local extracellular environment while ensuring that the simulated domain reproduced the conditions of an infinite periodic tissue. Within the RVE, the nucleus, cytoplasm, and PCM were all modelled as solid, linearly elastic continua, allowing the propagation of both compressional and shear waves throughout the intracellular and pericellular regions. The plasma membrane separating the cytoplasm from the ECM was represented as a thin structural shell, and its interaction with the cytoplasm was enforced through a structure–shell interface condition, ensuring continuity of displacements and tractions between the membrane and the underlying solid domains. Surrounding the cell, the ECM was treated as an acoustic pressure medium, supporting only longitudinal wave propagation and interacting with the solid regions via standard acoustic–structure boundary conditions that impose equality of normal accelerations and balance of normal stresses.

To emulate the repetitive, lattice-like arrangement of chondrocytes within cartilage and to ensure that the RVE behaves as part of a larger continuous medium rather than an isolated cell, periodic Floquet boundary conditions were applied on all faces of the cubic RVE. These conditions enforce that the acoustic pressure field and the solid displacement field on opposite faces differ only by a phase shift corresponding to the imposed excitation, effectively replicating an infinite periodic array of identical units, by applying Eq. (11),

11 $$u\left(r+R\right)={e}^{i\mathrm{k}\mathrm{R}}u(r)$$

where $u\left(r\right)$ is the displacement (or pressure) field, and R is a lattice translation vector representing the distance between two opposite faces of the RVE. The equation states that the field at one side of the periodic cell is identical to the field on the opposite side, except for a phase shift given by ${e}^{i\mathrm{k}\mathrm{R}}$, in which k is the wavevector of the incoming harmonic wave given by Eq. (12),

12 $${k}_{i}=\frac{\pi }{l}$$

where l is the distance between chondrocytes.

#### Macroscale modelling

**Fig. 2:**
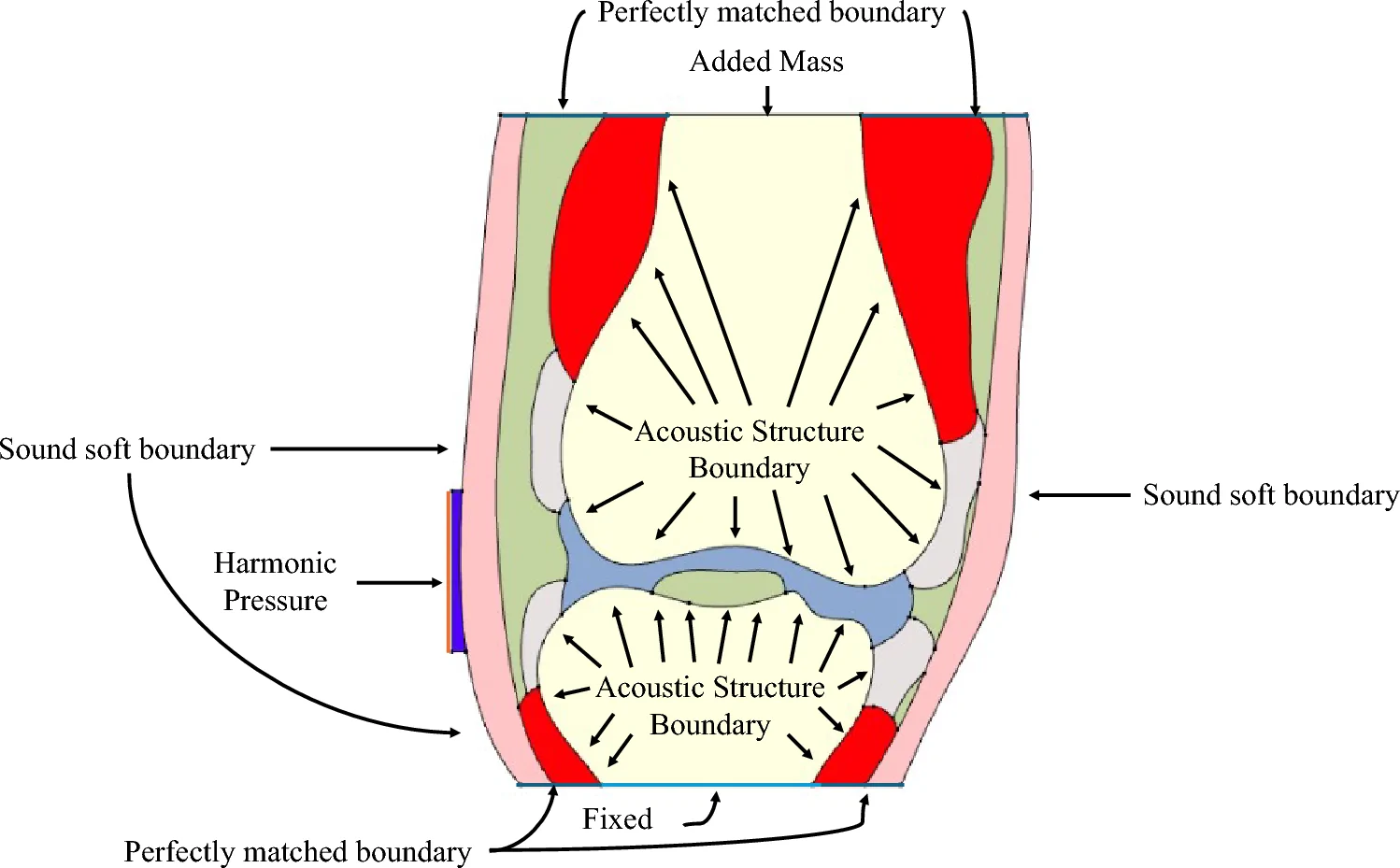
Schematic cross-sectional representation of the human knee joint used for simulation, highlighting the boundaries and the applied harmonic pressure

Figure 2 illustrates the computational domain and acoustic interaction within the knee joint used to investigate cartilage stimulation by acoustic waves.

The femoral and tibial regions, separated by articular cartilage and the meniscus, define acoustically heterogeneous domains in which wave propagation is governed by strong impedance contrasts. These interfaces are identified as acoustic structure boundaries, where reflection, transmission, and scattering of pressure waves dominate the local mechanical stimulus delivered to the cartilage.

The acoustic excitation is modelled as a harmonic pressure field incident in a coupling gel layer, representing the incoming wave generated by the ultrasonic transducer. This gel domain ensures efficient acoustic coupling between the transducer and the biological tissues while preserving realistic wavefront characteristics at the tissue interface. The transducer aperture is varied parametrically between 30 and 70 mm to evaluate the influence of source size on wave focussing, pressure distribution, and energy delivery to the cartilage.

**Fig. 3:**
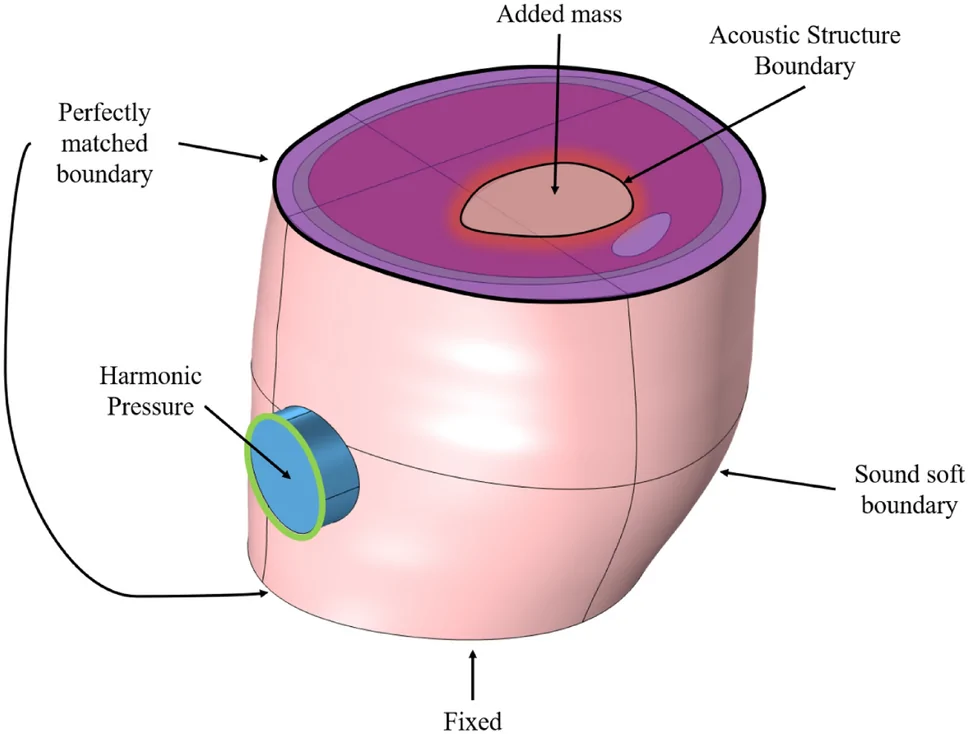
Schematic 3D representation of the human knee joint used for simulation, highlighting the boundaries and the applied harmonic pressure for patient A

Similarly, a three-dimensional anatomical model was developed using the imaging data and geometric characteristics associated with the patients, thereby enabling the corresponding patient-specific morphology to be incorporated into the numerical analysis, as can be seen in Fig. 3.

The frequency-dependent simulations had a spatial discretization selected to ensure numerical stability and accuracy by enforcing a Courant–Friedrichs–Lewy (CFL) condition of 0.2. For time-explicit simulation, the numerical model was solved using a discontinuous Galerkin formulation, using a CFL condition of 2/3 for this formulation coupled with a Runge–Kutta 34 Solver, ensuring that ${h}_{\mathrm{m}\mathrm{a}\mathrm{x}}\le 2{\lambda}_{\mathrm{m}\mathrm{i}\mathrm{n}}/3$. This has been shown to decrease computational cost and increase stability, without sacrificing accuracy (Toulorge and Desmet 2011; Laibe and Lombart 2021), taking into account that the internal solver time step was subsequently determined from the minimum element size and the maximum local wave speed to satisfy the CFL stability condition. However, this procedure necessitates element refinement in post-process. The element size was therefore adapted according to the local wavelength and excitation frequency, ensuring sufficient resolution of the propagating acoustic field within the cartilage layer.

Boundary conditions are defined to replicate physiological and open-domain behaviour. A soft sound boundary condition is applied at external tissue interfaces to minimize artificial reflections and approximate free-field radiation. Perfectly matched boundaries are implemented at the outer boundaries of the computational domain to enforce domain continuity and simulate an unbounded acoustic medium, allowing outgoing waves to be absorbed without reflection. An added mass of 55 kg was added, taking into account the average human body weight and removed the weight below the knee, as has been previously studied (Fernandes et al. 2025b).

To quantify cartilage stimulation, a surface integration of the acoustic pressure field is performed over the cartilage domain, yielding an averaged pressure value representative of the effective mechanical stimulus transmitted to the tissue.

### Simulation setup

#### Modal analysis setup

Modal analysis at the cellular scale was performed in COMSOL Multiphysics using a fully coupled multiphysics formulation that incorporated the solid mechanics, shell mechanics, and acoustic pressure domains relevant to the chondrocyte microenvironment. The nucleus, cytoplasm, and PCM were modelled as linear elastic solids governed by the Navier–Cauchy equation, enabling both longitudinal and shear elastic wave modes to develop within the intracellular space. The plasma membrane was represented as a thin structural shell, whose deformation was coupled to the cytoplasm through a structure–shell interface enforcing continuity of displacement and traction. Surrounding the cell, the ECM domain was modelled using the Pressure Acoustics, Frequency Domain interface, allowing propagation of acoustic pressure waves governed by the Helmholtz equation.

To correctly capture the interplay between these domains, COMSOL’s Acoustic–Structure Boundary conditions were applied at each solid–fluid interface, ensuring that normal accelerations and stresses remained continuous. This allowed pressure fluctuations in the ECM to drive elastic deformation in the solid regions, and conversely, solid vibrations to radiate pressure waves back into the fluid. The shell mechanics interface was similarly coupled to both solid domains to preserve mechanical continuity across the plasma membrane.

A Modal Analysis study was then executed on the fully coupled system, solving for the natural frequencies and eigenmodes of vibration of the composite cell–PCM–ECM structure.

Mode following was performed using the modal assurance criterion, M(i,j), to quantify the similarity between the displacement fields of modes obtained at consecutive stages of the parametric analysis, using Eq. (13),

13 $$M\left(i,j\right)=\frac{{\left|{\int}_{V}{{\boldsymbol{u}}}_{i}.{{\boldsymbol{u}}}_{j}^{\boldsymbol{*}}dV\right|}^{2}}{({\int}_{V}{{\boldsymbol{u}}}_{i}.{{\boldsymbol{u}}}_{j}^{\boldsymbol{*}}dV)({\int}_{V}{{\boldsymbol{u}}}_{j}.{{\boldsymbol{u}}}_{j}^{\boldsymbol{*}}dV)}$$

where ${\mathbf{u}}_{i}$ denotes the displacement eigenvector of reference mode i, ${\mathbf{u}}_{j}$ represents the displacement eigenvector of candidate mode j, the superscript ∗ indicates the complex conjugate, and V corresponds to the computational domain over which the displacement fields were compared. The criterion is normalized and varies between 0 and 1. A value approaching unity indicates a high degree of spatial correspondence between the two mode shapes, whereas a value close to zero denotes negligible similarity.

The first six modes considered relevant to the investigated mechanical response were selected from the reference configuration, were every chosen parameter was set to the minimum values. For each subsequent model condition or parameter value, every reference mode was compared with the available candidate eigenmodes by evaluating M(i,j). The candidate presenting the highest modal assurance value was assigned as the continuation of the corresponding reference mode. Once a candidate mode had been associated with a given reference mode, it was excluded from the remaining assignments to preserve a one-to-one correspondence between modal branches. This procedure was repeated independently for each of the six selected modes.

#### Harmonic analysis setup

Harmonic analysis was conducted in two stages: first at the microscale, using the RVE of a single chondrocyte embedded in its PCM and ECM, and subsequently at the macroscale, where the microscale response was coupled with a tissue-level acoustic model to evaluate pressure–strain transmissibility across scales.

At the cell (RVE) level, harmonic excitation was applied through the ECM domain using the Pressure Acoustics, Frequency Domain interface in COMSOL. A harmonic acoustic pressure p(f) of prescribed amplitude was imposed on one face of the periodic RVE, while Floquet periodic boundary conditions ensured that the domain behaved as part of an infinite lattice of repeating chondrocyte units. The acoustic pressure field was coupled to the solid mechanics domain through acoustic–structure boundaries, allowing harmonic pressure waves in the ECM to induce elastic deformation in the cytoplasm, nucleus, PCM, and plasma membrane shell. The resulting displacement field u(x,f) was used to compute the nuclear strain ε(f) via surface-averaged strain measures on the nuclear boundary. By sweeping across the relevant frequency range, the microscale frequency–response function was obtained using Eq. (14) to study transmissibility,

14 $$\left|{H}_{\varepsilon p}\left(f\right)\right|=\frac{\varepsilon (f)}{p(f)}$$

where ε(f) is the induced nuclear strain and p(f) is the applied harmonic pressure amplitude. This provided a direct quantification of how efficiently an incident acoustic wave at the tissue scale can deform the nucleus of an individual cell.

At the macroscale, harmonic analysis was performed on a continuum knee-level acoustic domain using a similar pressure-acoustics formulation, accounting for global tissue geometry and boundary conditions relevant to ultrasound stimulation, where the frequency response is given by Eq. (15),

15 $$\left|{H}_{pp}\left(f\right)\right|=\frac{{p}_{\mathrm{c}\mathrm{a}\mathrm{r}\mathrm{t}\mathrm{i}\mathrm{l}\mathrm{a}\mathrm{g}\mathrm{e}}(f)}{{p}_{\mathrm{t}\mathrm{r}\mathrm{a}\mathrm{n}\mathrm{s}\mathrm{d}\mathrm{u}\mathrm{c}\mathrm{e}\mathrm{r}}(f)}$$

where ${p}_{\mathrm{c}\mathrm{a}\mathrm{r}\mathrm{t}\mathrm{i}\mathrm{l}\mathrm{a}\mathrm{g}\mathrm{e}}(f)$ is the average pressure in the patient’s cartilage and ${p}_{\mathrm{t}\mathrm{r}\mathrm{a}\mathrm{n}\mathrm{s}\mathrm{d}\mathrm{u}\mathrm{c}\mathrm{e}\mathrm{r}}(f)$ is the applied harmonic pressure amplitude where the transducer is placed. The microscale RVE results were then incorporated through a multiscale coupling strategy, in which the pressure field computed at the macroscale served as the boundary input for the microscale model. At each point in the tissue exposed to harmonic excitation, the local ECM pressure amplitude obtained from the macroscale solution was passed to the corresponding RVE response function ${H}_{\varepsilon p}\left(f\right)$. This allowed the computation of location-dependent nuclear strain fields throughout the tissue without explicitly resolving every individual cell, by using Eq. (16).

16 $$\left| {H_{{{\mathrm{macro}} \to {\mathrm{micro}}}} \left( f \right)} \right| = H_{{\varepsilon p}} \left( f \right) \cdot H_{{pp}} \left( f \right)$$

This two-level harmonic simulation framework enabled the evaluation of pressure-to-strain transmissibility from the joint scale down to the intracellular scale, revealing how harmonic acoustic excitation propagates through cartilage and ultimately generates mechanotransductive nuclear deformation in both healthy and osteoarthritic chondrocytes.

#### Time-explicit simulations setup

Time-domain simulations were performed using an DG explicit time-integration scheme to resolve the transient propagation of acoustic and elastodynamic waves through the coupled fluid–structure system. This formulation was selected due to its numerical robustness for high-frequency wave propagation, its ability to accurately capture short-wavelength phenomena, and its suitability for modelling strongly coupled interfaces between acoustic media and deformable solids.

Both linear and nonlinear acoustic formulations with dissipation were considered. In the linear regime, wave propagation in fluid domains was governed by the classical linear acoustic wave equation (Eq. (1)), while solid domains (cells, cartilage, and bone where applicable) were described by linear elastodynamics. To evaluate whether finite-amplitude effects become relevant at the applied excitation levels, a nonlinear formulation based on the Westervelt equation (Eq. (4)) was additionally implemented in the fluid domains, incorporating quadratic pressure nonlinearity and frequency-dependent attenuation. By directly comparing linear and nonlinear responses under identical loading conditions, the necessity of including nonlinear effects at the target pressure amplitudes could be systematically assessed.

To prevent artificial wave reflections at the computational boundaries, perfectly matched boundaries were replaced by absorbing layers in the time-explicit framework. These absorbing regions were implemented with gradually increasing attenuation to mimic an unbounded domain and ensure stable dissipation of outgoing waves across the entire frequency range of interest. This approach is particularly well suited for transient simulations, where broadband signals and multiple reflections can otherwise contaminate the response.

Pressure excitation was applied as a transient input, allowing the system’s full temporal response to be captured. The resulting pressure fields, displacement fields, and strain histories were monitored within the extracellular matrix as well as at the cellular and nuclear levels. To excite a finite frequency band while preserving temporal localization, the pressure input was prescribed as a Gaussian-modulated tone burst, defined as Eq. (17),

17 $$p\left(t\right)={p}_{0}\mathrm{sin}\left(2\pi {f}_{0}t\right){e}^{-\frac{{(t-{t}_{0})}^{2}}{2{\sigma }^{2}}}$$

where ${p}_{0}$ is the peak pressure amplitude, ${f}_{0}$ the central excitation frequency, ${t}_{0}$ the temporal centre of the pulse, and σ controls the pulse duration and spectral bandwidth. This formulation enables controlled excitation of the dominant vibrational modes of the system while avoiding the nonphysical infinite bandwidth associated with impulsive loading, making it well suited for studying transient wave propagation and mechanotransductive strain in chondrocyte nuclei.

This time-explicit framework enabled direct comparison between linear and nonlinear wave propagation regimes, providing a quantitative basis to determine whether nonlinear acoustic effects significantly influence stress transmission at the desired wave amplitude and nuclear strain at the pressures relevant for mechanobiological stimulation.

### Sensitivity analysis

Sensitivity Analysis was performed to evaluate how variability in the model input parameters influenced the predicted quantities of interest (QoIs). Each uncertain input was represented by an appropriate probability distribution defined over a prescribed range, reflecting the expected variability or uncertainty associated with that parameter. The numerical model may therefore be expressed as Eq. (18),

18 $$Y=\mathcal{M}({X}_{1},{X}_{2},\dots , {X}_{n})$$

where Y denotes a scalar QoI, $\mathcal{M}$ represents the numerical model, ${X}_{i}$ is the i-th uncertain input parameter, and m is the total number of uncertain inputs.

A variance-based global sensitivity analysis was conducted using Sobol indices. In contrast to local sensitivity methods, which assess the model response near a specific set of nominal conditions, the Sobol approach considers the complete probability distribution assigned to each input (Sobol’ et al. 2007). The method decomposes the variance of the model output into contributions attributable to individual parameters and to interactions among them. Consequently, it enables the relative importance of each source of uncertainty to be quantified throughout the investigated parameter space.

For each input parameter ${X}_{i}$, the first-order Sobol index was calculated as Eq. (19),

19 $${S}_{i}=\frac{\mathrm{V}\mathrm{a}\mathrm{r} {X}_{i}[{\mathbb{E}}_{x\sim i}(Y|{X}_{i})]}{\mathrm{V}\mathrm{a}\mathrm{r} (Y)}$$

where ${S}_{{T}_{i}}$ represents the total contribution of ${X}_{i}$ to the output variance, encompassing both its direct effect and all interaction effects involving that parameter. The term ${\mathbb{E}}_{{X}_{i}}(Y\mid {\mathbf{X}}_{\sim i})$ is the expected response obtained by varying ${X}_{i}$ while holding the other inputs fixed. The corresponding variance measures the contribution of all parameters other than ${X}_{i}$; subtracting its normalized value from unity therefore isolates every variance component associated with ${X}_{i}$.

The difference between the total-effect and first-order indices ${S}_{Ti}-{S}_{i}$ provides an indication of the combined interaction effects involving ${X}_{i}$. A small difference suggests that the parameter acts predominantly through an independent contribution. Conversely, a pronounced difference indicates that its influence is strongly dependent on interactions with one or more of the remaining inputs.

The sensitivity analysis was conducted separately for each QoI. The frequencies of vibration modes retrieved by the simulation described in 2.4.1. and the FRF amplitudes described in 2.4.2. were prescribed the QoI. Parameters presenting high total-effect indices were interpreted as the principal sources of prediction uncertainty and the two most relevant were therefore considered priorities for improved characterization. Sobol indices theoretically range from 0 to 1, where a value of zero indicates that the input parameter does not contribute to the variance of the investigated quantity of interest, whereas a value of one denotes that the parameter accounts for the entire output variance. Higher index values therefore indicate a greater contribution of the corresponding input to prediction uncertainty.

### Deep neural network surrogate modelling

A DNN surrogate model was developed to reduce the computational cost associated with the parametric modal analyses performed in COMSOL Multiphysics. The surrogate was constructed after completion of the global sensitivity analysis, in which the two parameters presenting the highest total-order Sobol indices were selected as inputs to the surrogate model, as they accounted for the largest proportion of the output variance.

A design of experiments was generated over the prescribed variation ranges of the two selected parameters. The sampling domain was defined using the same lower and upper bounds adopted in the uncertainty quantification analysis. Parameter combinations were generated using uniform sampling, providing an approximately uniform coverage of the two-dimensional design space. For each sampled combination, the corresponding parameter values were transferred to the COMSOL model and a modal analysis was performed. The resulting natural frequencies of the first six relevant vibration modes and their respective amplitudes from the FRF were extracted and stored as the target variables of the surrogate model.

The resulting dataset therefore consisted of two input variables and six output variables, as is observed in Eqs. (20), (21) and (22),

20 $${\boldsymbol{x}}=\left[{x}_{1},{x}_{2}\right]$$

21 $${\boldsymbol{\omega}}=\left[{\omega}_{1},{\omega}_{2},{\omega}_{3},{\omega}_{4},{\omega}_{5},{\omega}_{6}\right]$$

22 $${\boldsymbol{y}}=\left[{y}_{1},{y}_{2},{y}_{3},{y}_{4},{y}_{5},{y}_{6}\right]$$

where ${x}_{1}$ and ${x}_{2}$ denote the two most influential parameters identified by the sensitivity analysis, and ${\omega}_{1}$ to ${\omega}_{6}$ represent the natural frequencies of the six tracked modes and ${y}_{1}$ to ${y}_{6}$ their respective amplitudes obtained from the FRF.

The available simulation results were randomly divided into training, validation, and test subsets, corresponding to 70%, 15%, and 15% of the complete dataset, respectively. The training subset was used to optimize the network parameters, while the validation subset was employed to monitor generalization performance and control overfitting. The test subset was retained until the end of the training procedure and was used exclusively for the independent assessment of predictive accuracy. The division was performed after dataset generation, and the distributions of both input parameters were verified to ensure comparable coverage across the three subsets.

Network training was formulated as a supervised regression problem. The model parameters were optimized by minimizing the mean squared error between the COMSOL results and the corresponding neural-network predictions, using Eq. (23),

23 $${\mathcal{L}}_{{{\mathrm{MSE}}}} = \frac{1}{{Nn_{m} }}\sum\nolimits_{n = 1}^{N} {\sum\nolimits_{m = 1}^{{n_{m} }} {\left( {f_{n,m}^{{{\mathrm{COMSOL}}}} - f_{n,m}^{{{\mathrm{DNN}}}} } \right)} }^{2} ,$$

where N is the number of training samples, ${n}_{m}=6$ is the number of tracked modes, ${f}_{n,m}^{\mathrm{C}\mathrm{O}\mathrm{M}\mathrm{S}\mathrm{O}\mathrm{L}}$ is the modal frequency calculated using the finite-element model, and ${f}_{n,m}^{\mathrm{D}\mathrm{N}\mathrm{N}}$ is the corresponding surrogate prediction.

The surrogate consisted of a fully connected feedforward neural network. The input layer contained two neurons, corresponding to the selected model parameters, while the output layer contained six neurons representing the predicted modal frequencies. The intermediate representation is presented individually in the supplemental material for of the models trained. Nonlinear activation functions were applied to the hidden layers, whereas a linear activation function was used in the output layer because the predicted quantities were continuous numerical values. The final network architecture was selected through manual testing, considering the number of hidden layers, number of neurons, learning rate, batch size, and regularization parameters.

Following validation, the trained network was used as a computationally efficient replacement for repeated COMSOL evaluations within the investigated parameter range. The training and validation of the used models is available in appendix A. New combinations of the two dominant parameters were supplied directly to the surrogate, which returned simultaneous predictions for the outputs. Because neural-network inference required only matrix operations, the computational time per evaluation was substantially lower than that required for a complete finite-element analysis. The surrogate was used only for interpolation within the parameter ranges represented in the training dataset; predictions outside these limits were not considered reliable without additional COMSOL simulations and subsequent model retraining.

## Results

### Micro-scale

#### Chondrocyte modal analysis

**Fig. 4:**
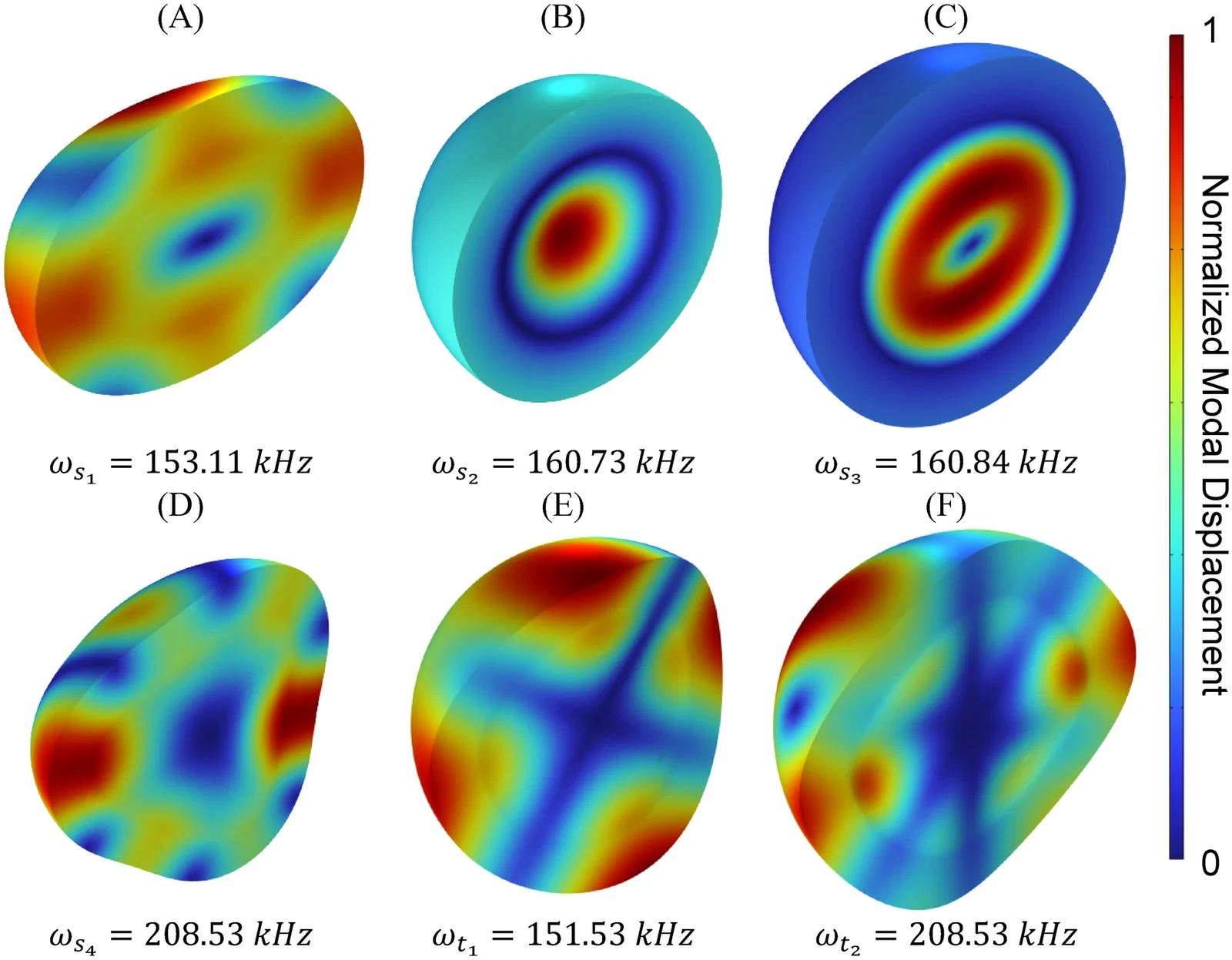
Fundamental vibration modes of healthy chondrocytes, without ASI, considering ${r}_{c}=5 \mu m$, ${t}_{\mathrm{P}\mathrm{C}\mathrm{M}}=2 \mu m$ and ${t}_{\mathrm{P}\mathrm{M}}=0.005 \mu m$, the vibration modes are: **A** ${s}_{1}$, **B** ${s}_{2}$, **C** ${s}_{3}$, **D** ${s}_{4}$, **E** ${t}_{1}$, and **F** ${t}_{2}$

The modal analysis of the chondrocytes modelling without being surrounded by the ECM is observed in Fig. 4.

The modal analysis performed on the chondrocyte model in the absence of the surrounding fluid reveals that the six most relevant vibration modes closely resemble those described for free elastic spheres in classical theory (Hosseini-Hashemi and Anderson 1988). These modes correspond to the characteristic spheroidal and torsional oscillations of an isotropic sphere, where spheroidal modes (${s}_{n}$) involve coupled radial and tangential motion, and torsional modes (${t}_{n}$) involve purely tangential shear deformation without volume change.

Among the identified vibration modes, a global ${s}_{0}$ mode corresponds to the breathing mode, a radially symmetric oscillation in which the entire cell expands and contracts uniformly, was not identified. Inspection of the mode shapes indicated that the ${s}_{2}$ and ${s}_{3}$ ​ modes produced the highest normalized displacement within the nuclear region, located at the centre of the spherical cell model. In these modes, the normalized deformation field compresses the nuclear region significantly, suggesting that it plays a dominant role in potential mechanotransductive stimulation of the nucleus when the cell is acoustically excited near its corresponding frequency. On this basis, the six identified modes were selected as the most suitable candidates for subsequent evaluation through the frequency–response function analysis.

**Fig. 5:**
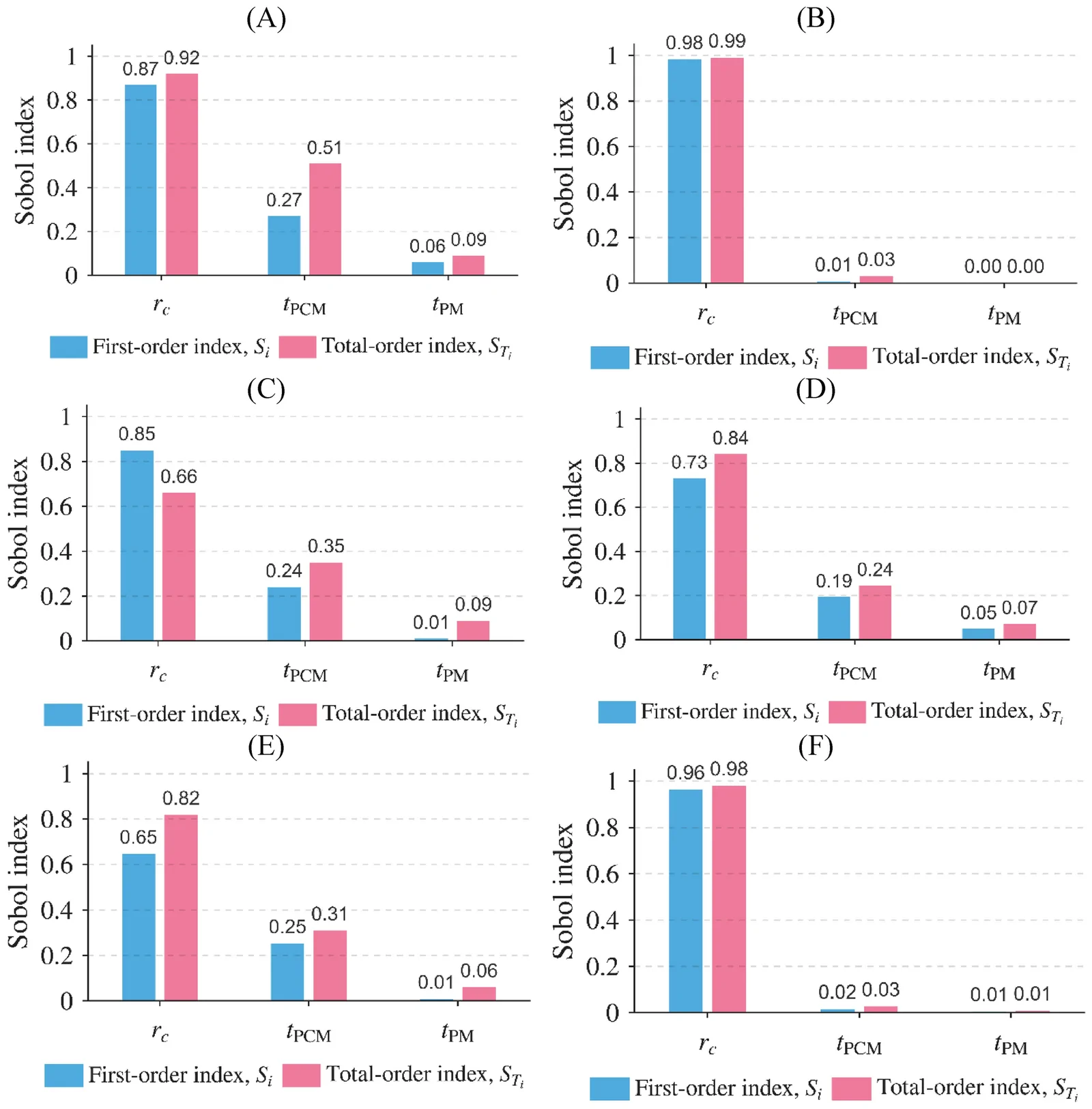
Sobol sensitivity indices quantifying the influence of the geometric parameters on the natural frequencies of a healthy chondrocyte model without the extracellular matrix (ECM): **A** ${s}_{1}$, **B** ${s}_{2}$, **C** ${s}_{3}$, **D** ${s}_{4}$, **E** ${t}_{1}$, and **F** ${t}_{2}$ vibration modes

The sensitivity analysis for healthy chondrocytes without ECM through the Sobol sensitivity indices is presented in Fig. 5.

The sensitivity analysis indicates that the chondrocyte radius is the principal geometric factor governing the natural frequencies of all six modes. Its influence is particularly pronounced for ${s}_{2}$ and ${t}_{2}$, for which the total-order indices reach 0.99 and 0.98, respectively, while the contributions of ${t}_{\mathrm{P}\mathrm{C}\mathrm{M}}$ and ${t}_{\mathrm{P}\mathrm{M}}$ remain negligible. A similarly dominant effect is observed for ${s}_{1}$ and ${s}_{4}$, although the PCM thickness also contributes to their variability, with total-order indices of 0.51 and 0.24, respectively. For ${t}_{1}$, ${r}_{c}$ remains the most influential parameter, followed by ${t}_{\mathrm{P}\mathrm{C}\mathrm{M}}$. The differences between the first- and total-order indices for ${s}_{1}$ and ${t}_{1}$ suggest that interactions involving the cell radius and PCM thickness contribute appreciably to the variance of these modal frequencies. By contrast, the plasma membrane thickness exhibits only a minor effect across the investigated modes, with total-order indices not exceeding 0.09. As ${r}_{c}$ and ${t}_{\mathrm{P}\mathrm{C}\mathrm{M}}$ consistently exhibited the greatest influence on the modal frequencies, the subsequent parametric analysis focussed on variations in these two geometric parameters.

**Fig. 6:**
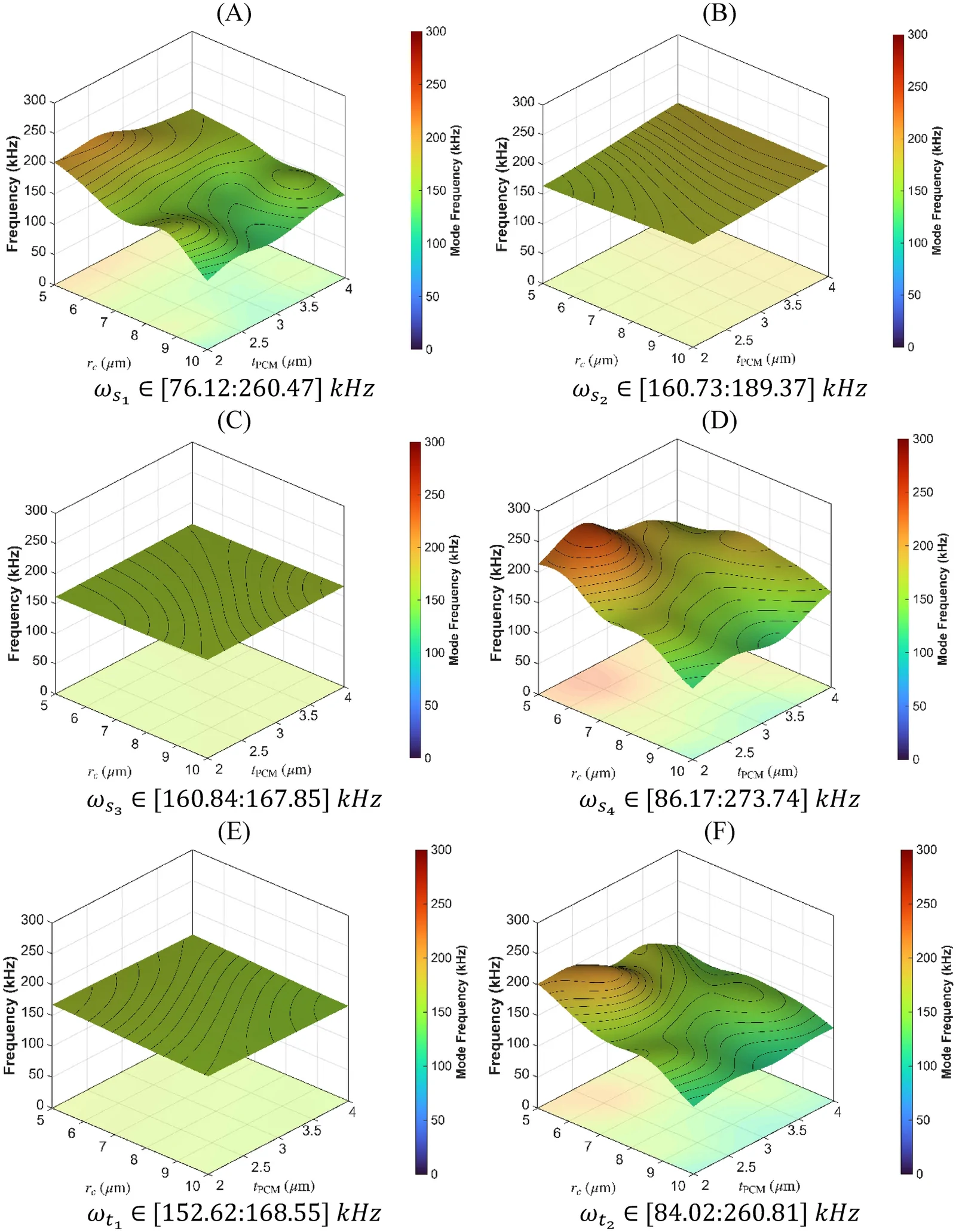
DNN surrogate predictions of the natural frequencies of a healthy chondrocyte model without the extracellular matrix as functions of chondrocyte radius (${r}_{c}$, 5–10 *µ*m) and pericellular matrix thickness (${t}_{\mathrm{P}\mathrm{C}\mathrm{M}}$, 2–4 *µ*m): **A** ${s}_{1}$, **B** ${s}_{2}$, **C** ${s}_{3}$, **D** ${s}_{4}$, **E** ${t}_{1}$, and **F** ${t}_{2}$

The parametric analysis performed using DNN surrogate model for healthy chondrocytes without ECM can be observed in Fig. 6.

The DNN-derived response surfaces demonstrate that the effects of ${r}_{c}$ and ${t}_{\mathrm{P}\mathrm{C}\mathrm{M}}$ differ substantially among the investigated vibration modes. ${s}_{1}$, ${s}_{4}$, and ${t}_{2}$ exhibit the greatest frequency variability across the geometric domain, with predicted ranges of 184.35, 187.57, and 176.79 kHz, respectively. Their curved surfaces and nonuniform contour spacing indicate pronounced nonlinear dependencies and combined effects between cell radius and PCM thickness. Consequently, relatively small geometric variations within specific regions of the parameter space may produce substantial shifts in the corresponding resonant frequencies. Conversely, ${s}_{2}$, ${s}_{3}$, and ${t}_{1}$ have a smaller frequency variation range in the selected inputs. ${s}_{3}$ shows the narrowest interval, varying by only 7.01 kHz, followed by ${t}_{1}$ and ${s}_{2}$, with variations of 15.93 and 28.64 kHz, respectively. Their comparatively smooth surfaces suggest a more gradual and predictable response to geometric variation. Overall, the results indicate that geometric variability should be considered on a mode-specific basis: ${s}_{1}$, ${s}_{4}$, and ${t}_{2}$ are particularly susceptible to changes in cellular dimensions, whereas ${s}_{3}$ and ${t}_{1}$ remain comparatively stable throughout the evaluated domain.

**Fig. 7:**
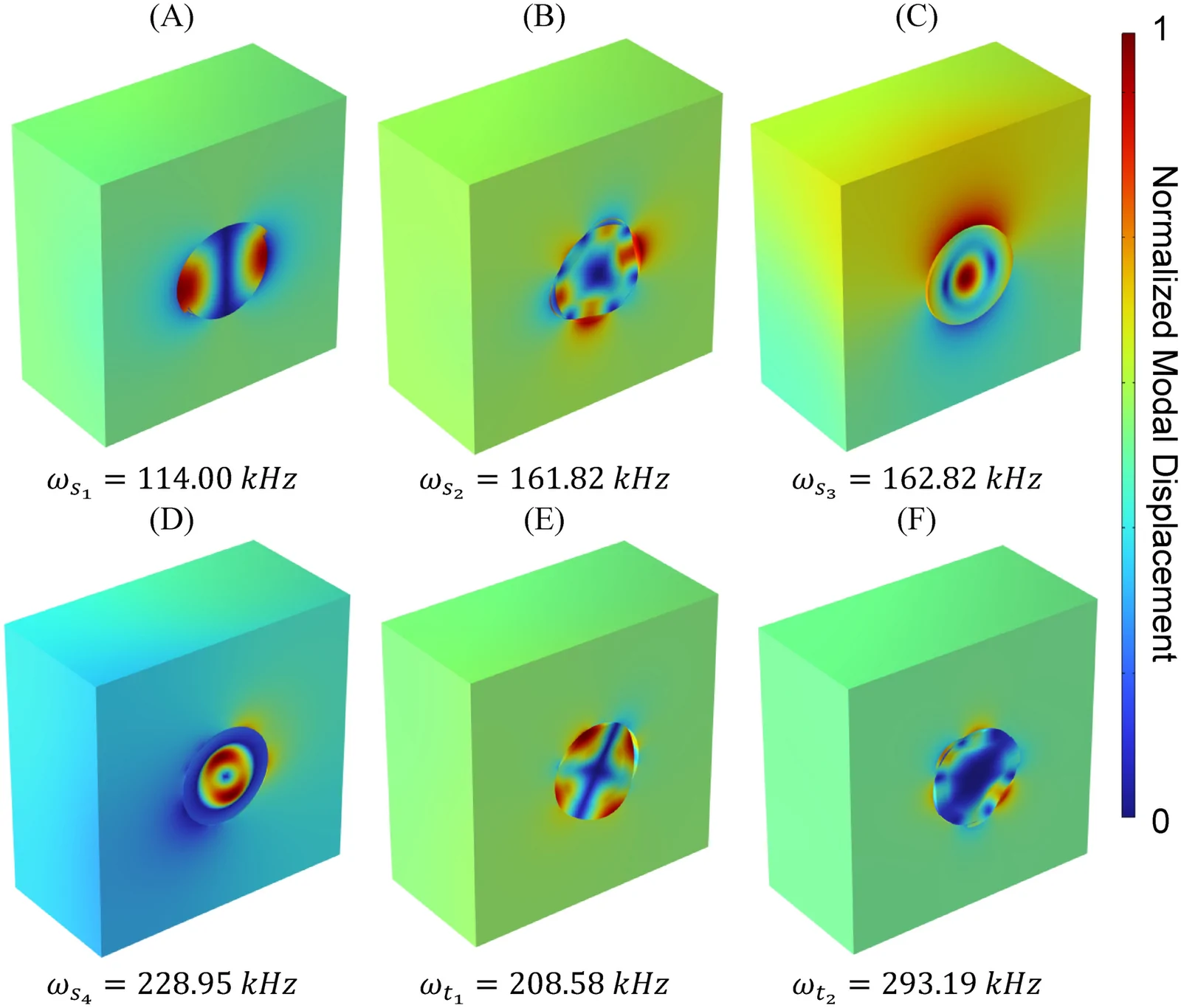
Fundamental vibration modes of healthy chondrocytes, with ASI, considering ${r}_{c}=5 \mu m$, ${t}_{\mathrm{P}\mathrm{C}\mathrm{M}}=2 \mu m$, ${t}_{\mathrm{P}\mathrm{M}}=0.005 \mu m$ and ${d}_{c}=40 \mu m$ where the vibration modes are: **A** ${s}_{1}$, **B** ${s}_{2}$, **C** ${s}_{3}$, **D** ${s}_{4}$, **E** ${t}_{1}$, and **F** ${t}_{2}$

A modal analysis was also performed considering the ECM as a fluid, to study how the acoustic-structure interaction would change the vibration modes of the system as shown in Fig. 7.

When the ECM is introduced and modelled as a surrounding fluid, the modal landscape changes significantly. The presence of the fluid introduces acoustic loading and viscous damping, which disrupt the discrete nature of the Lamb resonances. Instead of sharp eigenmodes, the system exhibits quasi-modes (broad, heavily damped responses with strong coupling between the solid and fluid domains). This damping effect is particularly pronounced for ${s}_{1}$, ${s}_{2}$, and ${s}_{3}$, whose energy is predominantly radial and thus strongly coupled to the surrounding medium.

**Fig. 8:**
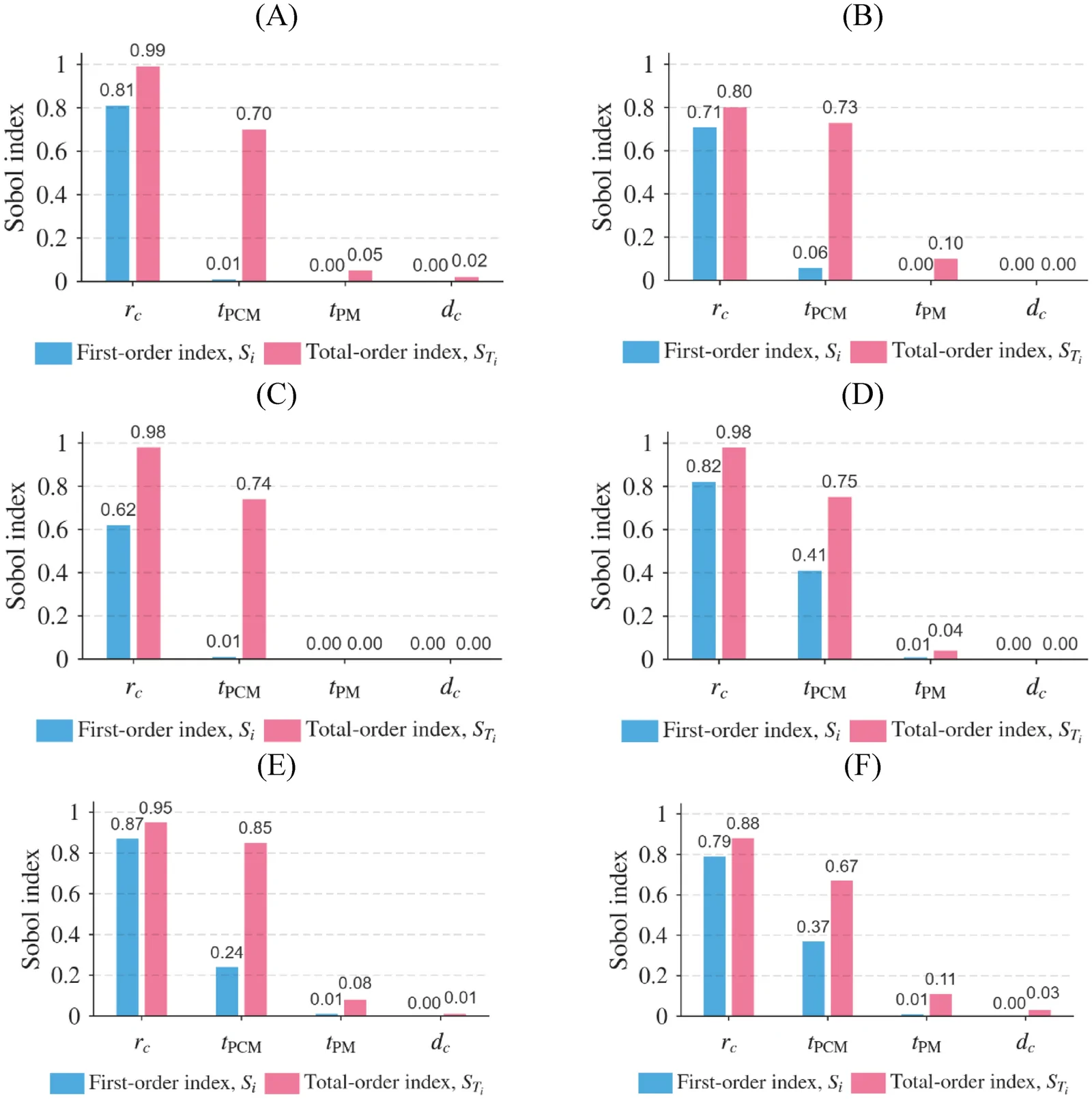
Sobol sensitivity indices quantifying the influence of geometric parameters on the natural frequencies of the healthy chondrocyte model embedded within the extracellular matrix (ECM): **A** ${s}_{1}$, **B** ${s}_{2}$, **C** ${s}_{3}$, **D** ${s}_{4}$, **E** ${t}_{1}$, and **F** ${t}_{2}$

The results of the Sobol-based global sensitivity analysis for healthy chondrocytes with ECM are presented in Fig. 8.

The chondrocyte radius was the predominant geometric parameter across all vibration modes, exhibiting first-order indices between 0.62 and 0.87 and total-order values ranging from 0.80 to 0.99. This result indicates that variations in cell size directly govern a substantial proportion of the predicted modal-frequency variance. The PCM thickness also exerted a considerable influence, with total-order indices of 0.70–0.85. Its markedly lower first-order contributions, particularly for ${s}_{1}$–${s}_{3}$, demonstrate that its effect arises primarily through interactions with other geometric inputs rather than through an isolated contribution. For ${s}_{4}$, ${t}_{1}$, and ${t}_{2}$, ${t}_{\mathrm{P}\mathrm{C}\mathrm{M}}$ displayed more appreciable independent effects, with ${S}_{i}$ values of 0.41, 0.24, and 0.37, respectively. In contrast, plasma membrane thickness had only a limited effect, with total-order indices not exceeding 0.11. Intercellular distance was essentially negligible throughout the analysis, reaching a maximum total-order value of 0.03. Overall, the results identify ${r}_{c}$ and ${t}_{\mathrm{P}\mathrm{C}\mathrm{M}}$ as the principal geometric determinants of the natural frequencies.

**Fig. 9:**
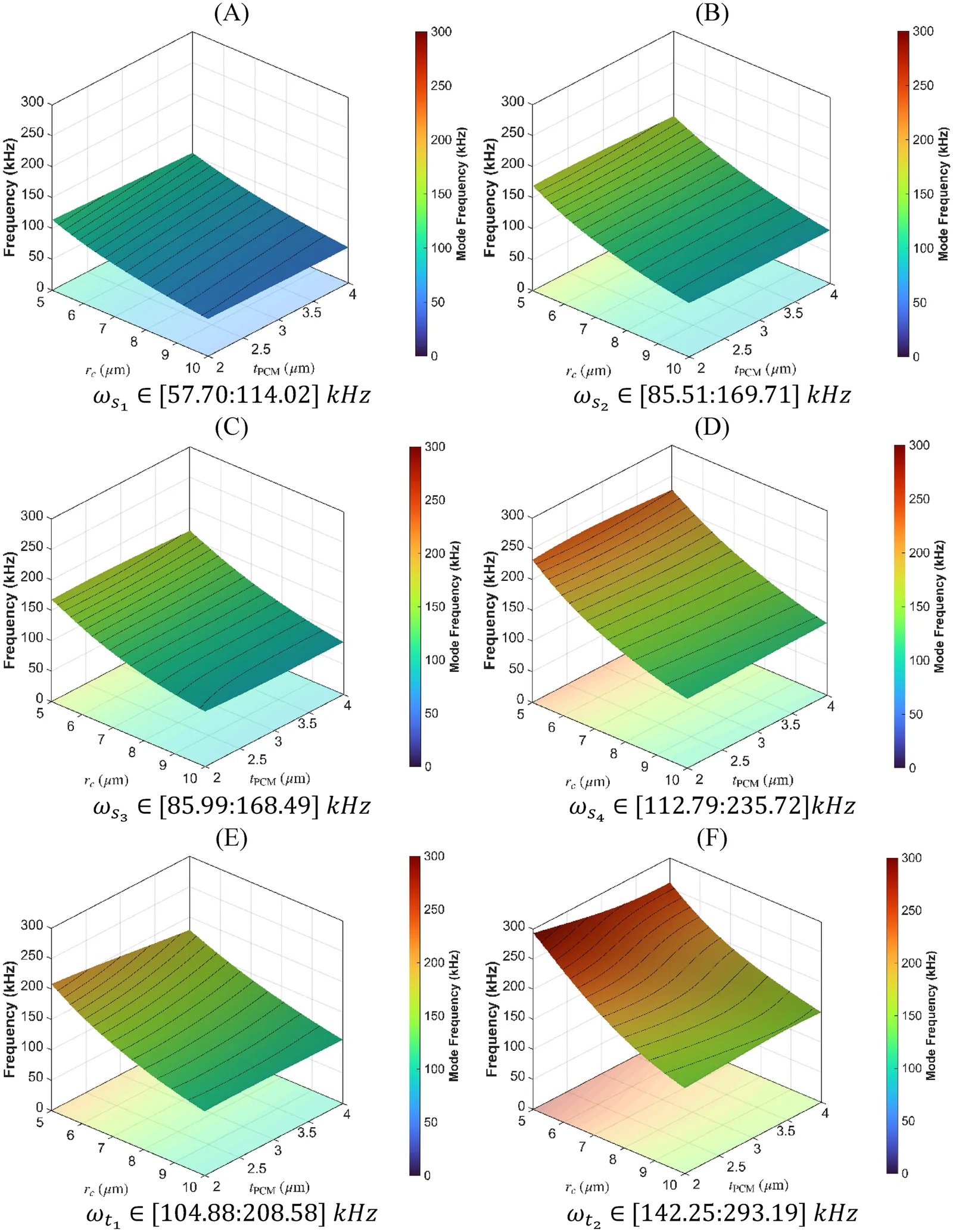
DNN surrogate predictions of the natural frequencies of a healthy chondrocyte model embedded within the ECM as functions of chondrocyte radius (${r}_{c}$, 5–10 *µ*m) and pericellular matrix thickness (${t}_{\mathrm{P}\mathrm{C}\mathrm{M}}$, 2–4 *µ*m): **A** ${s}_{1}$, **B** ${s}_{2}$, **C** ${s}_{3}$, **D** ${s}_{4}$, **E** ${t}_{1}$, and **F** ${t}_{2}$

The results of the parametric analysis for healthy chondrocytes with ECM conducted using the DNN surrogate model are presented in Fig. 9 Fig. 6.

The DNN response surfaces indicate a consistent reduction in modal frequency with increasing ${r}_{c}$, confirming cell radius as the predominant geometric determinant. Variations in ${t}_{PCM}$ provide an additional, mode-dependent modulation of the response. ${s}_{1}$–${s}_{4}$ exhibit comparatively regular surfaces, whereas ${t}_{1}$ and ${t}_{2}$ display greater curvature, suggesting a more pronounced nonlinear dependence on the selected parameters. Among the ECM-containing configurations, ${t}_{2}$ reaches the highest predicted frequency and presents the broadest interval (150.94 kHz), followed by ${s}_{4}$ (122.93 kHz) and ${t}_{1}$ (103.70 kHz). The inclusion of the ECM substantially modifies both the frequency intervals and the geometric sensitivity observed in the model without this constituent. For ${s}_{1}$ and ${s}_{4}$, the predicted ranges become narrower, decreasing from 184.35 to 56.32 kHz and from 187.57 to 122.93 kHz, respectively. Conversely, the variability of ${s}_{2}$, ${s}_{3}$, and ${t}_{1}$ increases markedly, particularly for ${s}_{3}$, whose interval expands from 7.01 to 82.50 kHz, and ${t}_{1}$, for which the range increases from 15.93 to 103.70 kHz. The ${t}_{2}$ frequencies shift towards higher values, although their overall span decreases moderately from 176.79 to 150.94 kHz. These changes demonstrate that the surrounding ECM does not produce a uniform frequency shift; instead, it alters the effective mechanical constraints and redistributes geometric sensitivity among the vibration modes. Consequently, the ECM should be explicitly represented when estimating resonance frequencies and evaluating their dependence on cellular geometry.

**Fig. 10:**
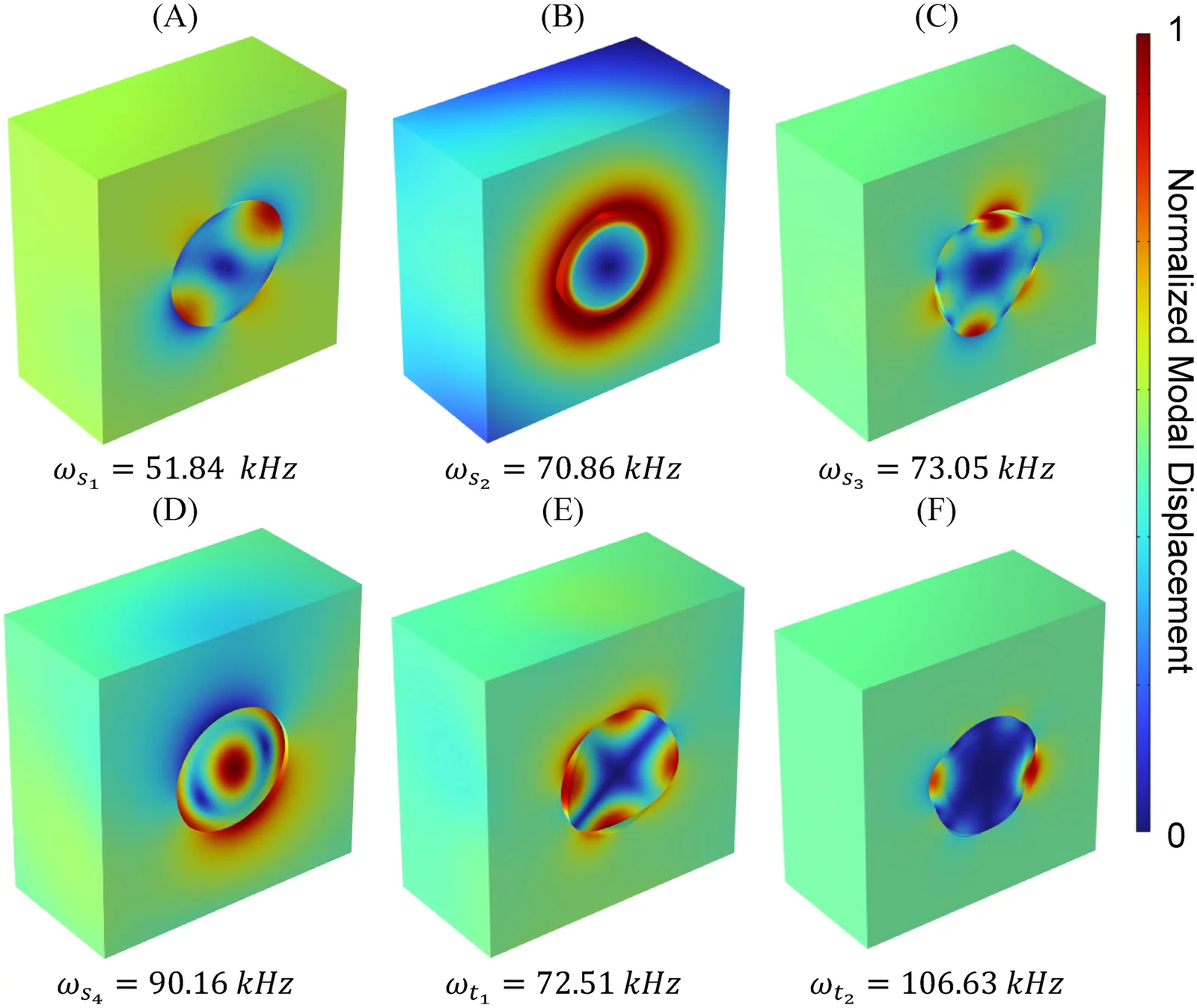
Fundamental vibration modes of OA chondrocytes, with ASI, considering ${r}_{c}=10 \mu m$, ${t}_{PCM}=1 \mu m$, ${t}_{PM}=0.005 \mu m$ and ${d}_{c}=50 \mu m$, the vibration modes are: **A** ${s}_{1}$, **B** ${s}_{2}$, **C** ${s}_{3}$, **D** ${s}_{4}$, **E** ${t}_{1}$, and **F** ${t}_{2}$

The same modal analysis was applied to OA chondrocytes, as can be seen in Fig. 10.

When the same modal analysis was applied to OA chondrocytes, all identified eigenfrequencies exhibited a marked downshift compared to healthy cells. This frequency decrease aligns with expectations from OA-related morphological and mechanical alterations: cell hypertrophy increases the effective mass of the system, while the loss of cytoskeletal integrity and reduction in elastic modulus lower its stiffness. Together, these factors shift the natural frequencies towards lower values, consistent with the general behaviour of a softening resonant structure.

**Fig. 11:**
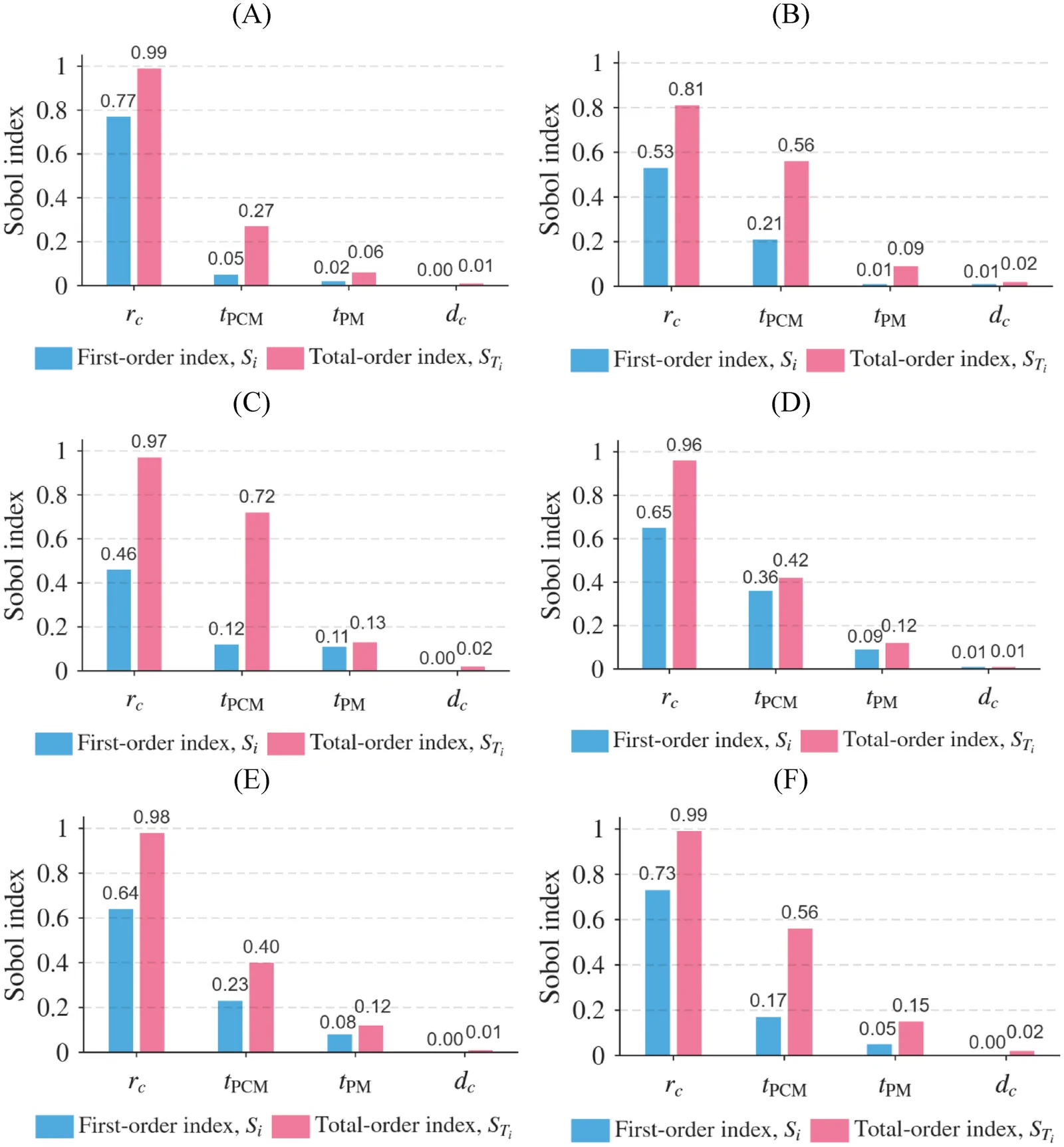
Sobol sensitivity indices quantifying the influence of geometric parameters on the natural frequencies of the OA chondrocyte model embedded within the ECM: **A** ${s}_{1}$, **B** ${s}_{2}$, **C** ${s}_{3}$, **D** ${s}_{4}$, **E** ${t}_{1}$, and **F** ${t}_{2}$

The results of the Sobol-based global sensitivity analysis of the OA chondrocyte vibration modes are presented in Fig. 11.

The chondrocyte radius remains the principal determinant of modal-frequency variability in the OA configuration, with total-order indices ranging from 0.81 to 0.99. Its first-order contribution varies between 0.46 and 0.77, indicating a substantial direct effect across all six modes. The difference between the first- and total-order indices is particularly pronounced for ${s}_{3}$, for which ${S}_{i}=0.46$ and ${S}_{{T}_{i}}=0.97$. This result suggests that the influence of ${r}_{c}$ on this mode is strongly mediated by interactions with other geometric parameters. The PCM thickness represents the second most influential input, although its contribution is mode dependent. Its total-order index ranges from 0.27 for ${s}_{1}$ to 0.72 for ${s}_{3}$, with relevant effects also observed for ${s}_{2}$ and ${t}_{2}$ (${S}_{{T}_{i}}=0.56$). Plasma membrane thickness has a comparatively limited influence, reaching a maximum total-order value of 0.15 for ${t}_{2}$. Intercellular distance remains negligible, with ${S}_{{T}_{i}}\le 0.02$ for all modes. Compared with the healthy model, OA reduces the independent contribution of ${r}_{c}$, while its total-order influence remains similarly high. This wider separation between ${S}_{i}$ and ${S}_{{T}_{i}}$ indicates that osteoarthritic alterations increase the relative importance of parameter interactions. The influence of ${t}_{\mathrm{P}\mathrm{C}\mathrm{M}}$ is generally lower than in the healthy configuration, particularly for ${s}_{1}$, ${s}_{4}$, and ${t}_{1}$, whose total-order indices decrease from 0.70, 0.75, and 0.85–0.27, 0.42, and 0.40, respectively. By contrast, the plasma membrane exhibits a modest increase in relevance for several OA modes. Overall, the pathological configuration preserves cell radius as the dominant geometric factor but redistributes part of the modal-frequency variance towards interaction effects and, to a lesser extent, plasma membrane thickness.

**Fig. 12:**
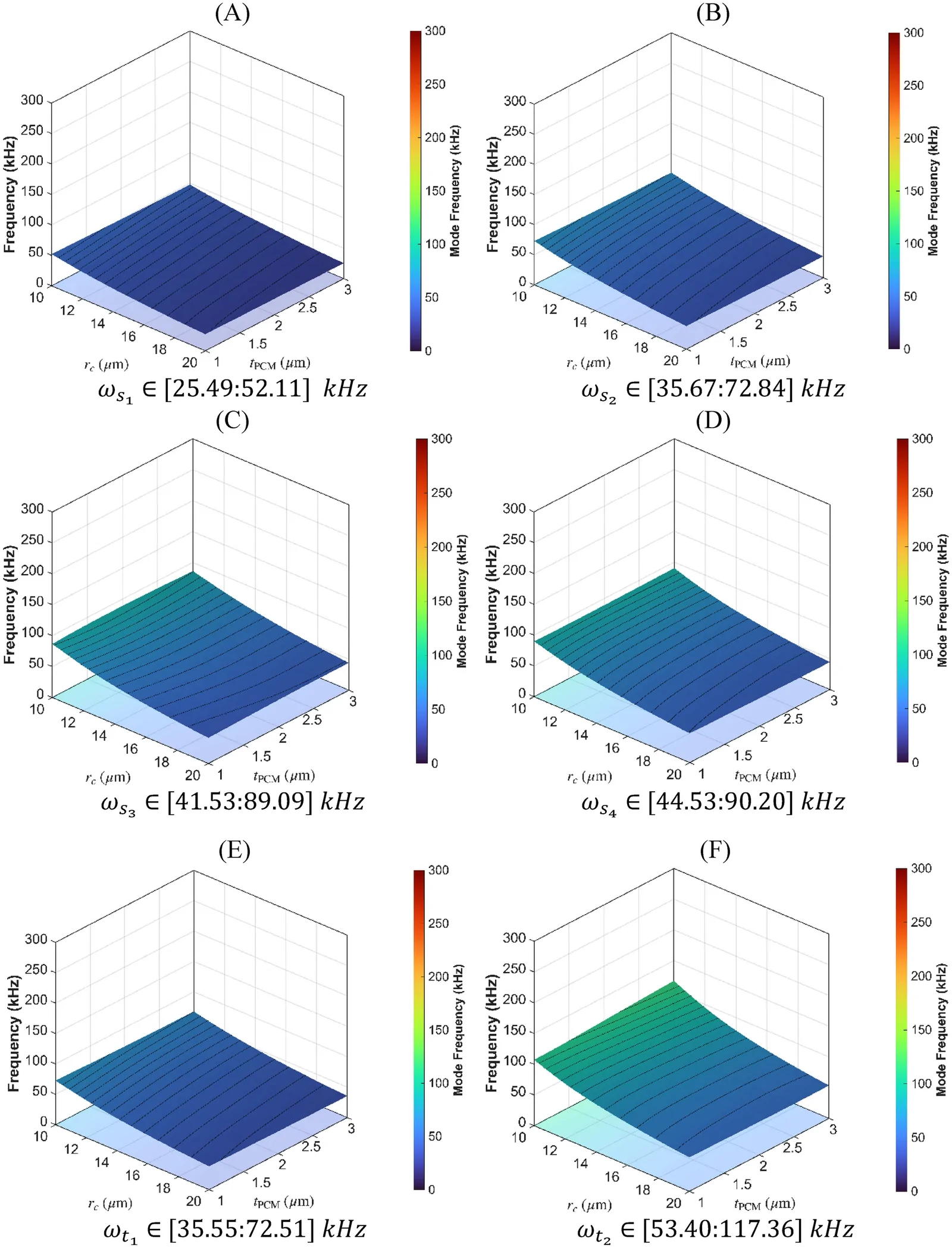
DNN surrogate predictions of the natural frequencies of the osteoarthritic (OA) chondrocyte model embedded within the ECM as functions of chondrocyte radius (${r}_{c}$, 10–20 *µ*m) and pericellular matrix thickness (${t}_{PCM}$, 1–3 *µ*m): **A** ${s}_{1}$, **B** ${s}_{2}$, **C** ${s}_{3}$, **D** ${s}_{4}$, **E** ${t}_{1}$, and **F** ${t}_{2}$

 Fig. 12 presents the DNN surrogate-based parametric analysis of OA chondrocytes embedded within the ECM.

The DNN-derived surfaces show that the modal frequencies decrease consistently with increasing ${r}_{c}$, confirming chondrocyte radius as the principal geometric determinant in the OA model. Variations in ${t}_{\mathrm{P}\mathrm{C}\mathrm{M}}$ produce a smaller but mode-dependent modulation of the predicted response. T2 exhibits the highest frequencies and the widest interval (63.96 kHz), whereas S1 presents the lowest values and the narrowest range (26.62 kHz). The relatively regular surfaces indicate a predominantly monotonic dependence within the investigated domain, although their curvature suggests that interactions between ${r}_{c}$ and ${t}_{\mathrm{P}\mathrm{C}\mathrm{M}}$ remain relevant. Compared with the healthy ECM-containing model, the OA configuration produces a pronounced downward shift in all six frequency bands. The upper limits predicted for S1, S2, S4, T1, and T2 remain below the corresponding healthy lower bounds, while only limited overlap is observed for S3. Furthermore, the frequency ranges become narrower under OA conditions, decreasing from 56.32–150.94 kHz in the healthy model to 26.62–63.96 kHz in the pathological configuration, depending on the mode. The greatest reduction in variability occurs for T2, whose interval decreases from 150.94 to 63.96 kHz. These findings indicate that the combination of OA-associated material alterations, cellular hypertrophy, and changes in PCM geometry shifts the modal response towards lower frequencies and reduces its variation across the evaluated parameter space.

While previous studies on chondrocyte ultrasound stimulation have targeted frequencies in the MHz range (ideally 5 MHz for suspended cells) (Louw et al. 2013; Miller et al. 2017), our model predicts the dominant nuclear‐coupled mode ${s}_{3}$ at much lower frequencies in the kHz band (healthy: 63.81–162.12 kHz; OA: 26.979–51.84 kHz). This discrepancy reflects the realistic embedding of the cell in the PCM/ECM and the consequent additional acoustic‐structure loading and damping. Accordingly, applying ultrasound or vibration at MHz frequencies may excite other modes, but might not efficiently drive the nuclear‐strain mode in situ. Our results thus provide a rationale for shifting stimulus design towards the kHz band when targeting nucleus‐centric mechanotransduction in chondrocytes.

#### Micro frequency results

**Fig. 13:**
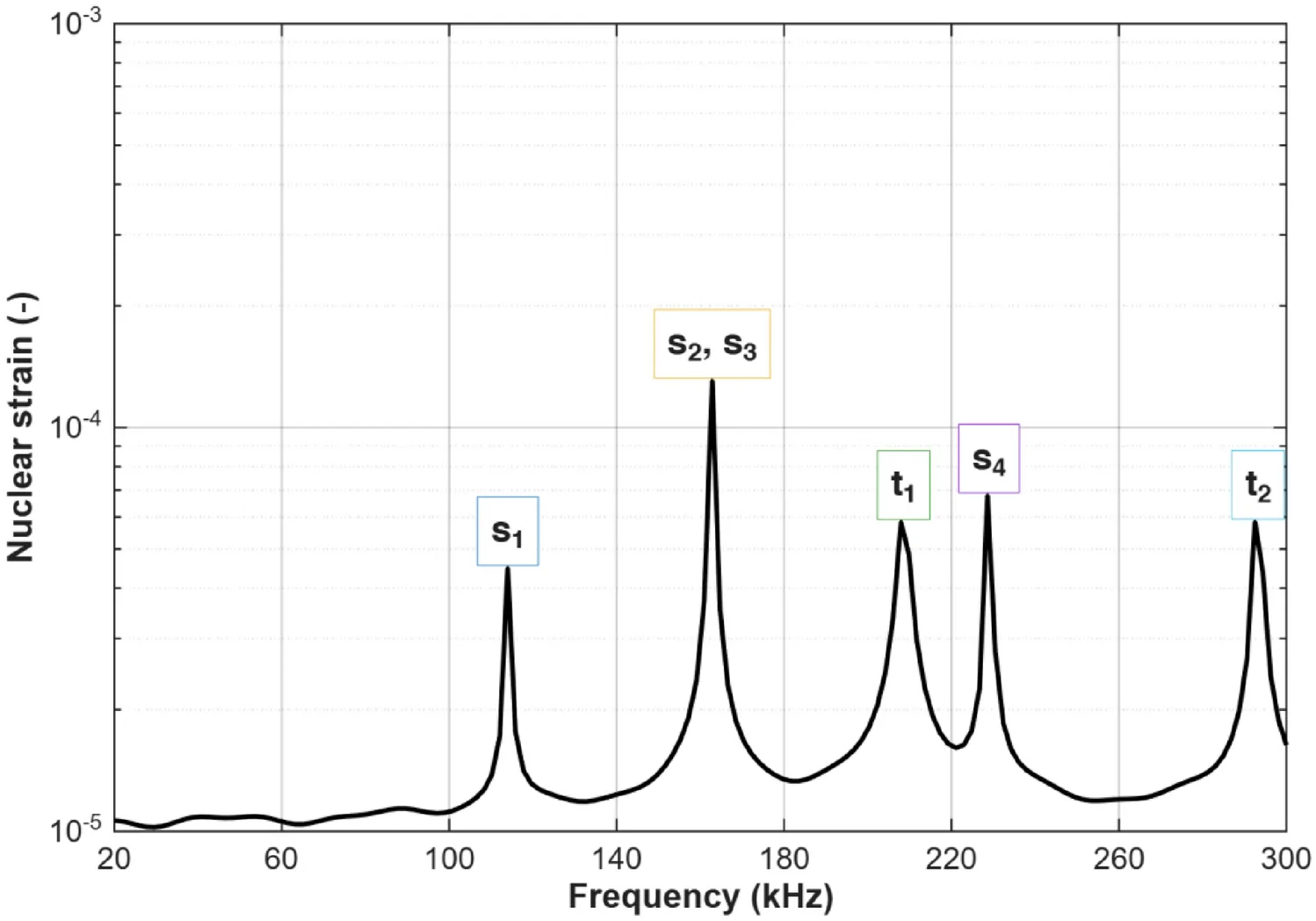
Frequency response of a healthy chondrocyte, considering ${r}_{c}=5 \mu m$, ${t}_{\mathrm{P}\mathrm{C}\mathrm{M}}=2 \mu m$, ${t}_{\mathrm{P}\mathrm{M}}=0.005 \mu m$ and ${d}_{c}=40 \mu m$

The harmonic analysis of the RVE of the healthy chondrocyte cell is visualized in Fig. 13.

The initial FRF analysis revealed a distinct hierarchy among the vibrational modes in terms of strain amplification. The coupled ${s}_{2},{s}_{3}$ mode exhibited the highest response, reaching a nuclear strain of 1.24·10^–4^, followed by ${s}_{4}$ with 9.11·10^–5^, and ${t}_{2}$ with 7.52·10^–5^. The ${s}_{3}$ and ${s}_{4}$ mode, which had been identified in the modal analysis as the one most strongly coupling with the nucleus, thus produced the highest peaks, while ${s}_{1}$ and ${t}_{1}$ showed the lowest peaks, with a nuclear strain of 3.49·10^–5^ and 9.11·10^–5^, respectively. Although there was a coupled ${s}_{2},{s}_{3}$ mode, it is possible that as parameters change, these modes might become isolated.

**Fig. 14:**
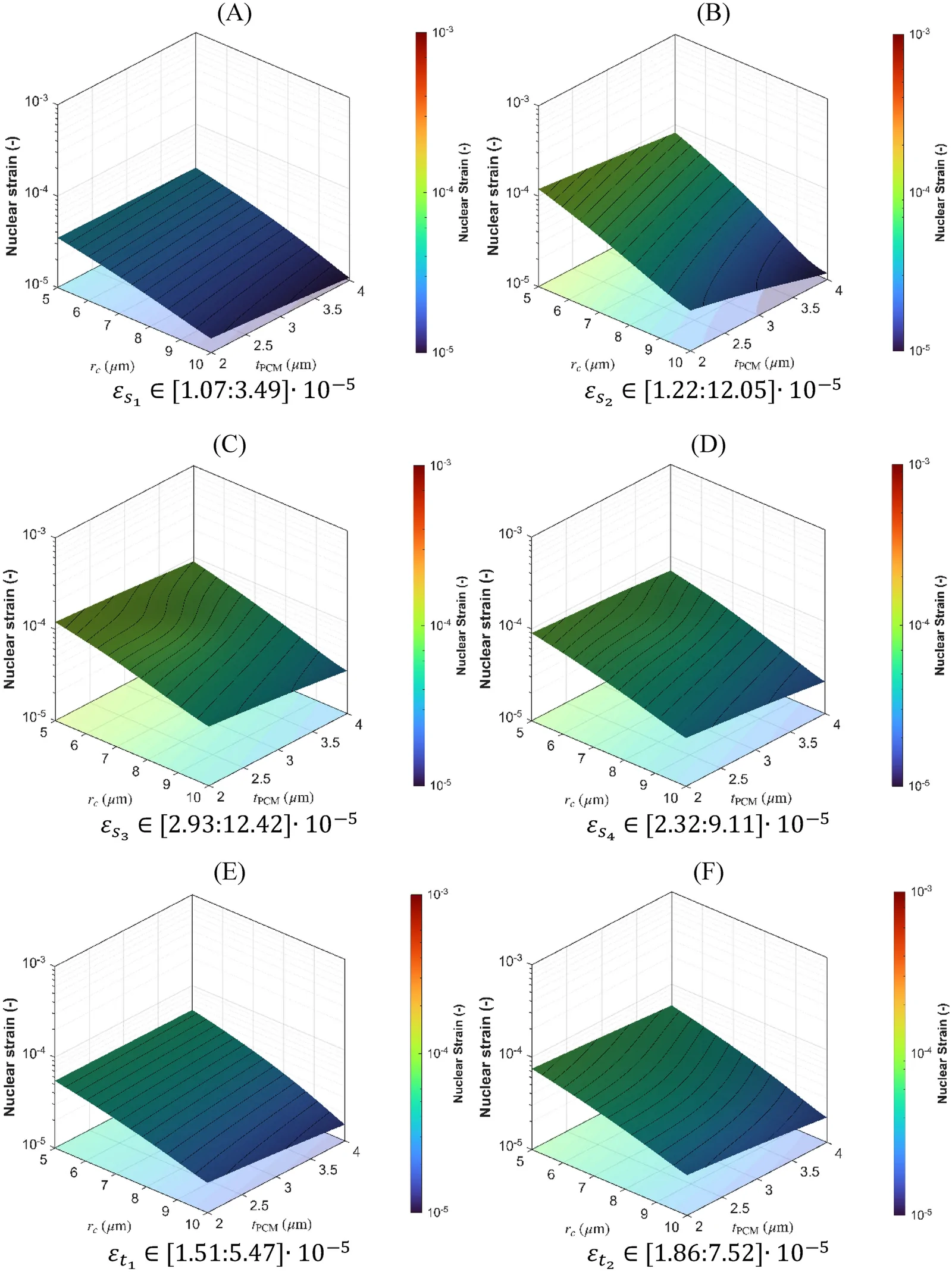
Parametric variation in the nuclear strain predicted by the DNN surrogate model for healthy chondrocytes as a function of chondrocyte radius, ${r}_{c}$, and pericellular matrix thickness, ${t}_{\mathrm{P}\mathrm{C}\mathrm{M}}$: **A** ${s}_{1}$, **B** ${s}_{2}$, **C** ${s}_{3}$, **D** ${s}_{4}$, **E** ${t}_{1}$, and **F** ${t}_{2}$

A parametric sweep was performed in the geometric parameters as outlined in Table 1, in order to observe how it could change the FRF of the chondrocytes, as can be observed in Fig. 14.

The DNN-derived response surfaces indicate that chondrocyte radius exerted the strongest geometrical influence on nuclear strain across all six vibration modes, with larger cells generally producing lower deformation levels. By comparison, variations in PCM thickness generated more moderate, mode-dependent changes. This behaviour suggests that cellular size governs the principal redistribution of deformation within the chondrocyte–PCM system, whereas PCM thickness modifies the mechanical transmission between the extracellular environment and the intracellular compartments.

The ${s}_{1}$ mode exhibited the lowest overall response and a comparatively smooth variation throughout the parameter space, with predicted nuclear strain ranging from $1.07\cdot {10}^{-5}$ to $3.49\cdot {10}^{-5}$. Conversely, the ${s}_{2}$, ${s}_{3}$, and ${s}_{4}$ modes generated substantially greater maximum values, reaching $12.05\cdot {10}^{-5}$, $12.42\cdot {10}^{-5}$, and $9.11\cdot {10}^{-5}$, respectively. The similarity between the upper response regions of ${s}_{2}$ and ${s}_{3}$, particularly for smaller chondrocyte radii, indicates that these modes initially produced comparable levels of nuclear deformation. Nevertheless, their surfaces progressively separated as the geometry changed. In particular, the ${s}_{2}$ response decreased more sharply and reached a markedly lower minimum of $1.22\cdot {10}^{-5}$, whereas ${s}_{3}$ retained a higher minimum of $2.93\cdot {10}^{-5}$. Thus, although the two modal branches were initially associated with similar strain responses, their distinct sensitivities to ${r}_{c}$ and ${t}_{\mathrm{P}\mathrm{C}\mathrm{M}}$ led to a progressive decoupling across the parametric domain. This separation indicates that the corresponding deformation patterns evolved independently as cellular geometry changed, reducing their similarity in terms of nuclear strain magnitude. Modes ${t}_{1}$ and ${t}_{2}$ displayed intermediate behaviour. The ${t}_{1}$ surface showed a relatively regular and nearly monotonic decrease, while ${t}_{2}$ presented greater curvature and a wider strain interval.

**Fig. 15:**
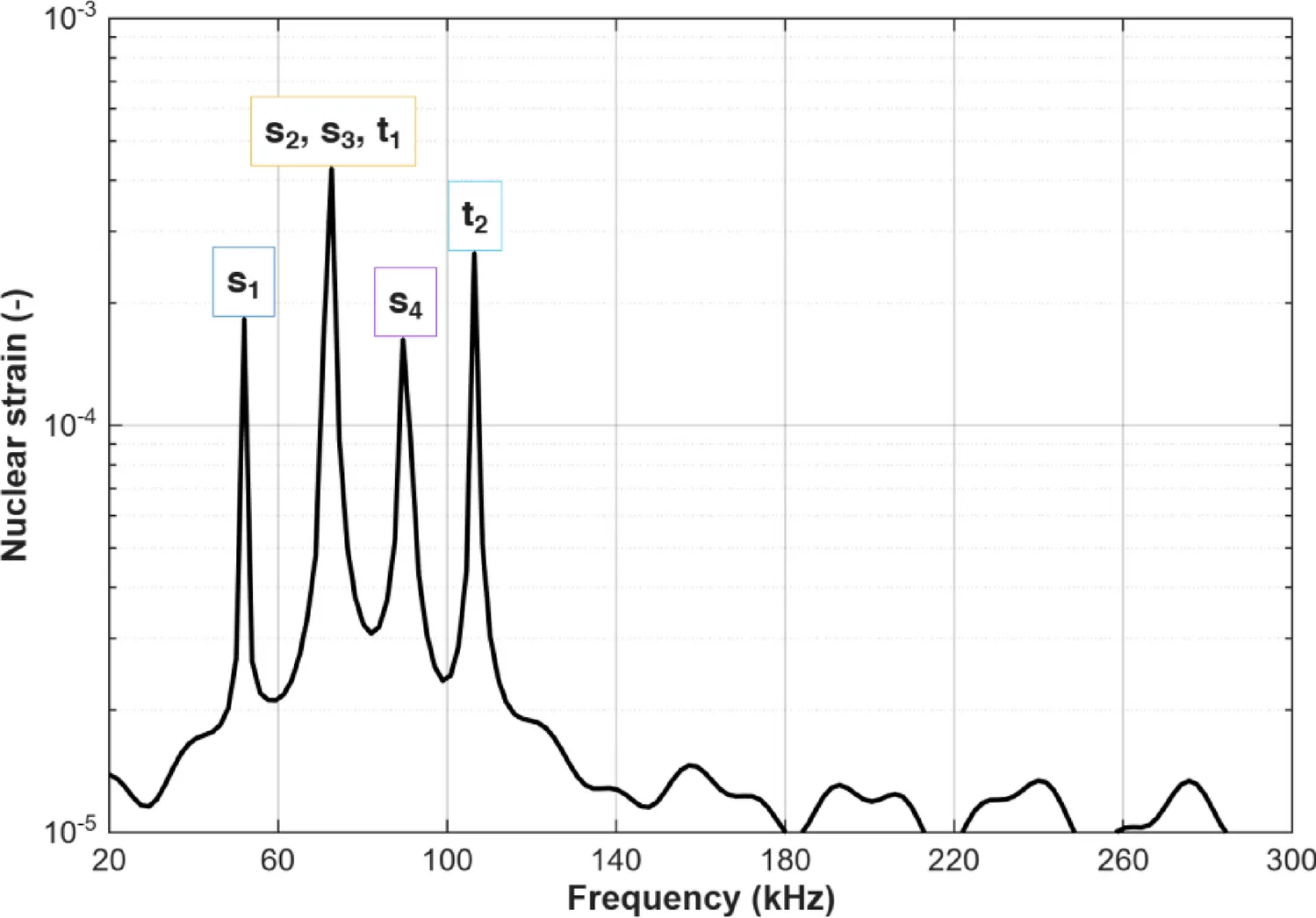
Frequency response of a OA chondrocyte, considering ${r}_{c}=10 \mu m$, ${t}_{\mathrm{P}\mathrm{C}\mathrm{M}}=1 \mu m$, ${t}_{\mathrm{P}\mathrm{M}}=0.005 \mu m$ and ${d}_{c}=50 \mu m$

The same harmonic analysis of the RVE was performed on an OA chondrocyte cell as can be seen in Fig. 15.

For OA chondrocytes, the FRF analysis revealed a clear reorganization of the modal hierarchy when compared with healthy cells. The coupled ${s}_{2}, {s}_{3},{t}_{1}$ mode became the dominant contributor, with a much higher nuclear strain of 4.02·10^–4^, over an order of magnitude larger than any mode in the healthy configuration. This was followed by ${t}_{2}$ (2.74·10^–4^), ${s}_{1}$​ (1.89·10^–4^), and ${s}_{4}$ (1.65·10^–4^). Thus, whereas in both healthy cells the ${s}_{3}$ drove the largest dynamic nuclear strain under harmonic pressure excitation. The much narrower frequency shift with the diseased state suggests that osteoarthritic hypertrophy and softening not only lower the natural frequencies, but also redistribute how external acoustic energy is partitioned among vibrational modes, potentially altering which patterns of deformation are most efficiently excited in the nuclear region.

**Fig. 16:**
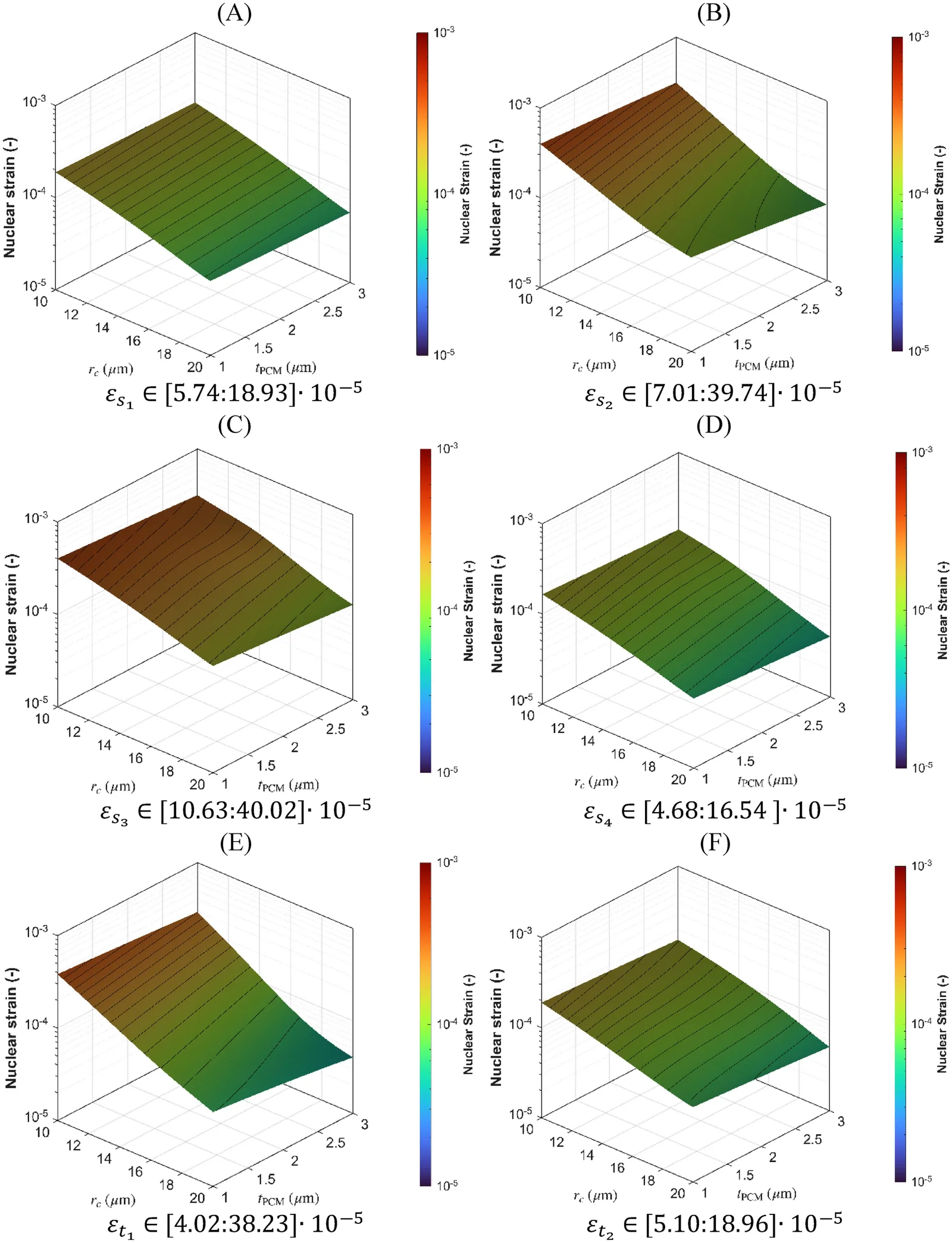
DNN surrogate-model predictions of nuclear strain in osteoarthritic chondrocytes as a function of chondrocyte radius, ${r}_{c}$, and pericellular matrix thickness, ${t}_{\mathrm{P}\mathrm{C}\mathrm{M}}$, for the first six tracked vibration modes: **A** ${s}_{1}$, **B** ${s}_{2}$, **C** ${s}_{3}$, **D** ${s}_{4}$, **E** ${t}_{1}$, and **F** ${t}_{2}$

The same parametric analysis was repeated for osteoarthritic chondrocytes. The resulting trends are presented in Fig. 16.

The surrogate-model predictions show that nuclear strain generally decreased with increasing chondrocyte radius across all vibration modes. PCM thickness produced a weaker but mode-dependent contribution, indicating that cellular enlargement was the dominant geometric factor governing nuclear deformation within the investigated osteoarthritic domain. The highest strain was predicted for ${s}_{3}$, ranging from $10.63\cdot {10}^{-5}$ to $80.16\cdot {10}^{-5}$, followed by ${s}_{2}$, with values between $7.01\cdot {10}^{-5}$ and $39.74\cdot {10}^{-5}$. Modes ${s}_{1}$, ${s}_{4}$, and ${t}_{2}$ exhibited lower and comparatively uniform responses, whereas ${t}_{1}$ showed a pronounced nonlinear reduction as the cell radius increased.

A comparison with the healthy configuration reveals a substantial increase in the nuclear strain predicted for the osteoarthritic cell. In the healthy model, the maximum values for ${s}_{2}$ and ${s}_{3}$ were $12.05\cdot {10}^{-5}$ and $12.42\cdot {10}^{-5}$, respectively. These maxima increased to $39.74\cdot {10}^{-5}$ and $80.16\cdot {10}^{-5}$ in the osteoarthritic geometry, corresponding to significant increases. This amplification may be associated with the combined influence of cellular hypertrophy and changes in the thickness and mechanical properties of the PCM, which alter load transmission towards the nucleus.

In the healthy configuration, ${s}_{2}$ and ${s}_{3}$ generated similar maximum strain levels, suggesting that the two modal branches initially induced comparable nuclear deformation. In the osteoarthritic model, the response of ${s}_{3}$ was coupled with that of ${s}_{2}$ and ${t}_{1}$, with values of nuclear strain that didn’t follow the same trend as parameters changed. This progressive separation indicates that ${s}_{2}$ and ${t}_{1}$ became decoupled from ${s}_{3}$ as the cellular geometry and surrounding microenvironment changed. More precisely, the three tracked modes developed distinct sensitivities to the geometric parameters: ${s}_{3}$ retained a stronger capacity to concentrate deformation within the nucleus, whereas the amplification of ${s}_{2}$ and ${t}_{1}$ remained comparatively limited. Thus, osteoarthritic remodelling did not affect the modal responses uniformly but promoted a clear differentiation between deformation mechanisms that were comparatively similar in the healthy model.

These findings identify ${s}_{3}$ as the mode most strongly associated with nuclear deformation in the osteoarthritic configuration.

The microscale FRFs reveal distinct frequency-dependent responses between the healthy and OA configurations. The healthy configuration displayed a lower but broader response, with modal contributions distributed over a wider portion of the spectrum. Its principal response occurred near 70–120 kHz, with two peaks at 76.87 kHz and 111.77 kHz with a response of 3.77·10^–5^ ± 1.27·10^–5^ Pa^−1^ and 4.43·10^–5^ ± 1.76·10^–5^ Pa^−1^, followed by secondary features around 200–260 kHz with the highest peak of 4.43·10^–5^ ± 7.69·10^–6^ Pa^−1^ detected at 103.55 kHz. In contrast, the OA model exhibited substantially greater compliance at lower frequencies, with its dominant response occurring approximately between 25 and 120 kHz and reaching a maximum near of 3.28·10^–4^ ± 6.05·10^–5^ Pa^−1^ at 58.29 kHz and 1.82·10^–4^ ± 3.15·10^–5^ Pa^−1^ at 100.54 kHz. Additional local maxima were observed around 100–110 and 135–145 kHz. These results indicate that osteoarthritic alterations shift the principal deformation response towards lower frequencies while increasing its magnitude. The OA response decreased sharply above approximately 110 kHz and approached its baseline level beyond 170 kHz, with limited evidence of higher-frequency resonances. The two curves became comparable around 155–165 kHz, after which the healthy model generally maintained the greater compliance until approximately 285 kHz.

**Fig. 17:**
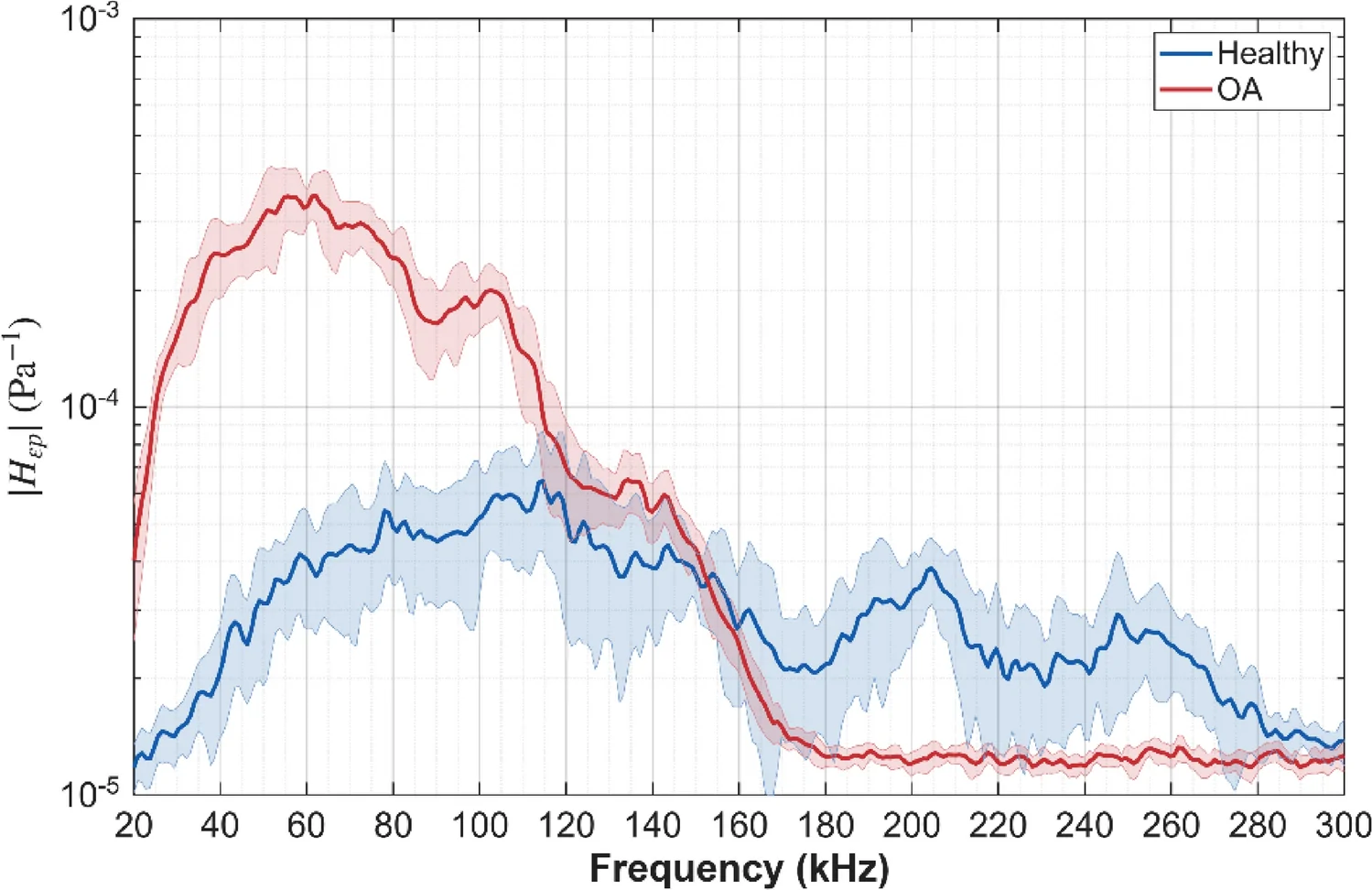
Average microscale FRFs predicted for healthy and OA chondrocyte models over the 20–300 kHz frequency interval

The increased low-frequency response of the OA model is consistent with the combined effects of cellular enlargement and degradation of the surrounding mechanical environment, particularly PCM softening (Pascarelli et al. 2015). These changes reduce the effective stiffness of the chondrocyte–PCM system and promote stronger deformation at lower excitation frequencies. Conversely, the wider spectral distribution observed for healthy chondrocytes suggests that their comparatively stiffer and more mechanically integrated microenvironment supports modal activity over a broader frequency range. The shaded regions further demonstrate that the healthy response was generally more sensitive to geometrical variability at intermediate and higher frequencies, whereas OA variability was concentrated predominantly around the principal low-frequency resonances.Taken together, these comparisons demonstrate that while the qualitative influence of each geometric parameter is preserved between healthy and OA conditions, OA cells consistently exhibit lower resonance frequencies, reduced modal sharpness, and increased dependence on PCM mechanics, reflecting the biomechanical degradation inherent to osteoarthritic tissue (Nahian and Sapra 2025; Chawla et al. 2022) (Fig. 17).

### Macro-scale

#### Macro frequency response

**Fig. 18:**
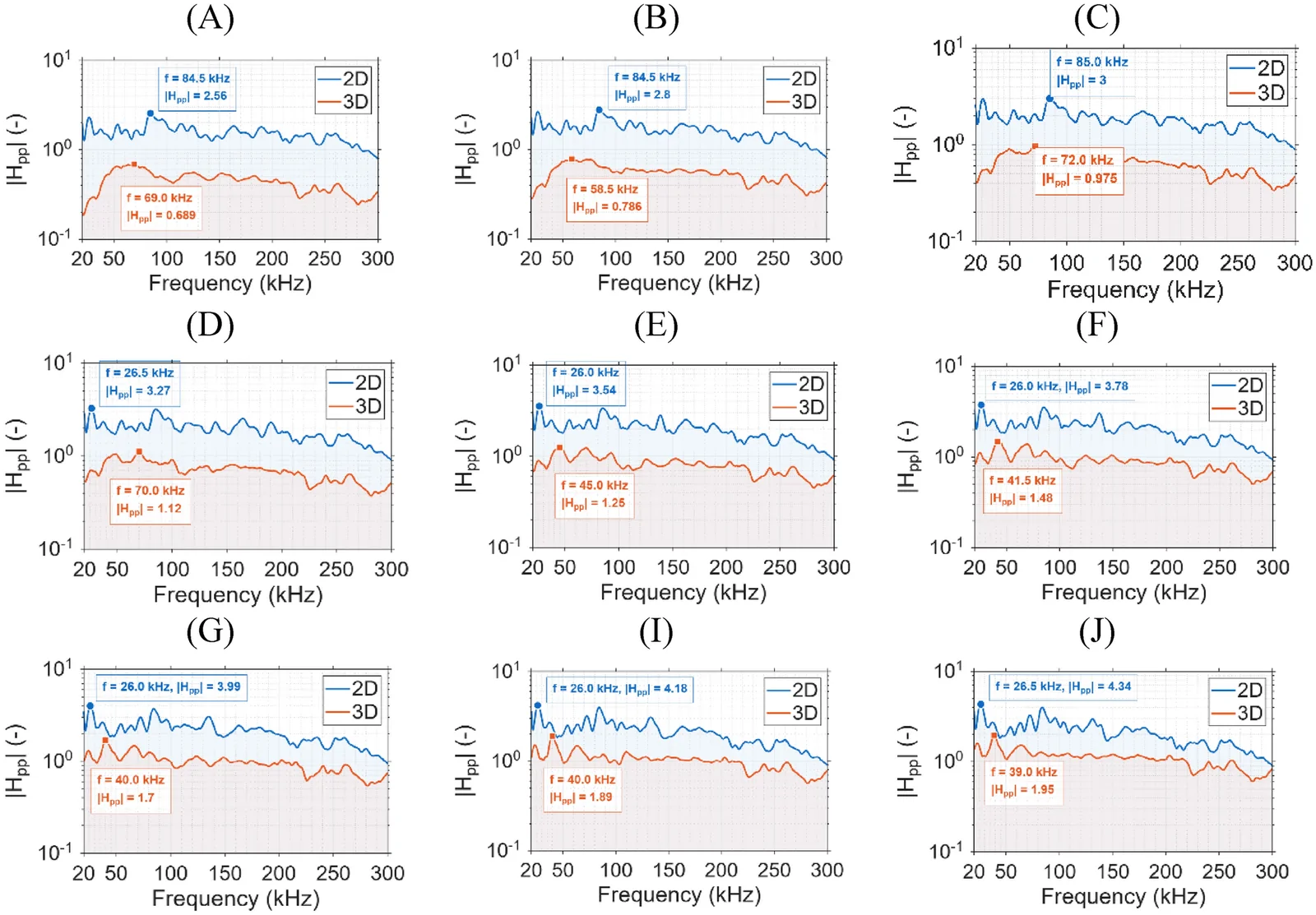
Macroscale frequency–response functions obtained from the cartilage-integrated probe for the 2D and 3D knee models using transducer diameters of **A** 30 mm, **B** 35 mm, **C** 40 mm, **D** 45 mm, **E** 50 mm, **F** 55 mm, **G** 60 mm, **I** 65 mm, and **J** 70 mm

The transfer function of the acoustic response to cartilage for Patient A (Fig. 18) demonstrates a consistent frequency-dependent behaviour across transducer diameter ranging from 30 to 70 mm.

The macroscale results show that the 2D model consistently predicted a greater cartilage response than its 3D counterpart throughout most of the investigated frequency range. For the 30 mm transducer, the maximum 2D response was $\mid {H}_{pp}\mid =2.56$ at 84.5 kHz, whereas the 3D model reached $\mid {H}_{pp}\mid =0.689$ at 69.0 kHz. At 35 mm, these maxima increased to 2.80 at 84.5 kHz and 0.786 at 58.5 kHz, respectively. The 40 mm configuration produced peaks of 3.00 at 85.0 kHz in 2D and 0.975 at 72.0 kHz in 3D.

A transition in the dominant 2D response occurred between transducer diameters of 40 and 45 mm. For diameters of 30–40 mm, the global maximum remained near 85 kHz. At 45 mm, however, the dominant peak shifted to 26.5 kHz and reached 3.27. The peak frequency subsequently remained approximately constant at 26.0–26.5 kHz, while its magnitude progressively increased to 3.54, 3.78, 3.99, 4.18, and 4.34 for the 50, 55, 60, 65, and 70 mm transducers, respectively. This behaviour indicates that increasing the excitation area altered the relative prominence of the frequency-dependent propagation mechanisms, causing the low-frequency component to become dominant in the 2D formulation.

The 3D results also showed a progressive amplification with increasing diameter, although the associated peak frequency varied more gradually. The maximum response increased from 0.689 at 30 mm to 1.12 at 45 mm, with the latter occurring at 70.0 kHz. For larger transducers, the dominant peak shifted towards lower frequencies, decreasing from 45.0 kHz at 50 mm to 41.5 kHz at 55 mm, 40.0 kHz at both 60 and 65 mm, and 39.0 kHz at 70 mm. The corresponding amplitudes increased systematically from 1.25 to 1.48, 1.70, 1.89, and 1.95. Accordingly, the largest 3D response was obtained with the 70 mm transducer, reaching $\mid {H}_{pp}\mid =1.95$ at 39.0 kHz.

Although the 2D predictions remained higher throughout the parametric study, the relative difference between the two models decreased as transducer diameter increased. The ratio between the maximum 2D and 3D responses declined from approximately 3.72 for the 30 mm configuration to 2.23 for the 70 mm case.

For both configurations, the response was generally greater at low and intermediate frequencies and gradually decreased towards 300 kHz. Several local fluctuations were observed, reflecting interference among transmitted, reflected, and geometrically scattered waves within the heterogeneous knee structure. Nevertheless, these features formed comparatively broad response regions rather than sharply defined resonances.

Increasing the transducer diameter produced an overall rise in the cartilage response, particularly in the 3D simulations. The difference between the two formulations consequently became less pronounced for the largest diameters, although the 2D predictions remained higher. This tendency suggests that a larger excitation area improves acoustic coupling and directs a greater proportion of the incident wave field towards the cartilage. For larger transducer diameters, the convergence of the ${H}_{pp}$ curves should be interpreted with caution. Since ${H}_{pp}$ was computed over the finite cartilage integration area, an increase in aperture size beyond the dimensions effectively coupled to the cartilage may not produce a proportional increase in the area-averaged pressure response. In this condition, part of the acoustic beam may bypass the cartilage region, leading to a plateau associated with spatial averaging over a bounded anatomical region rather than definitive stabilization of the entire acoustic field. Therefore, the observed convergence indicates reduced sensitivity of the cartilage-averaged ${H}_{pp}$ to further increases in transducer diameter, rather than complete wave-field robustness.

Across all patients, a similar overall trend was observed (Appendix B, Figs. 26–33, Appendix C, Figs. 34–41). Although absolute amplitude levels and minor peak shifts varied due to anatomical differences, the frequency-dependent shape of the transfer function and the convergence behaviour at larger transducer distances were consistent. In all cases, the dominant amplification regions occurred within comparable frequency bands. This suggests that, within the modelled range, transducer positioning beyond this threshold does not substantially influence the frequency-domain transmission characteristics to cartilage, reinforcing the robustness of the observed acoustic response across patient-specific geometries.

**Fig. 19:**
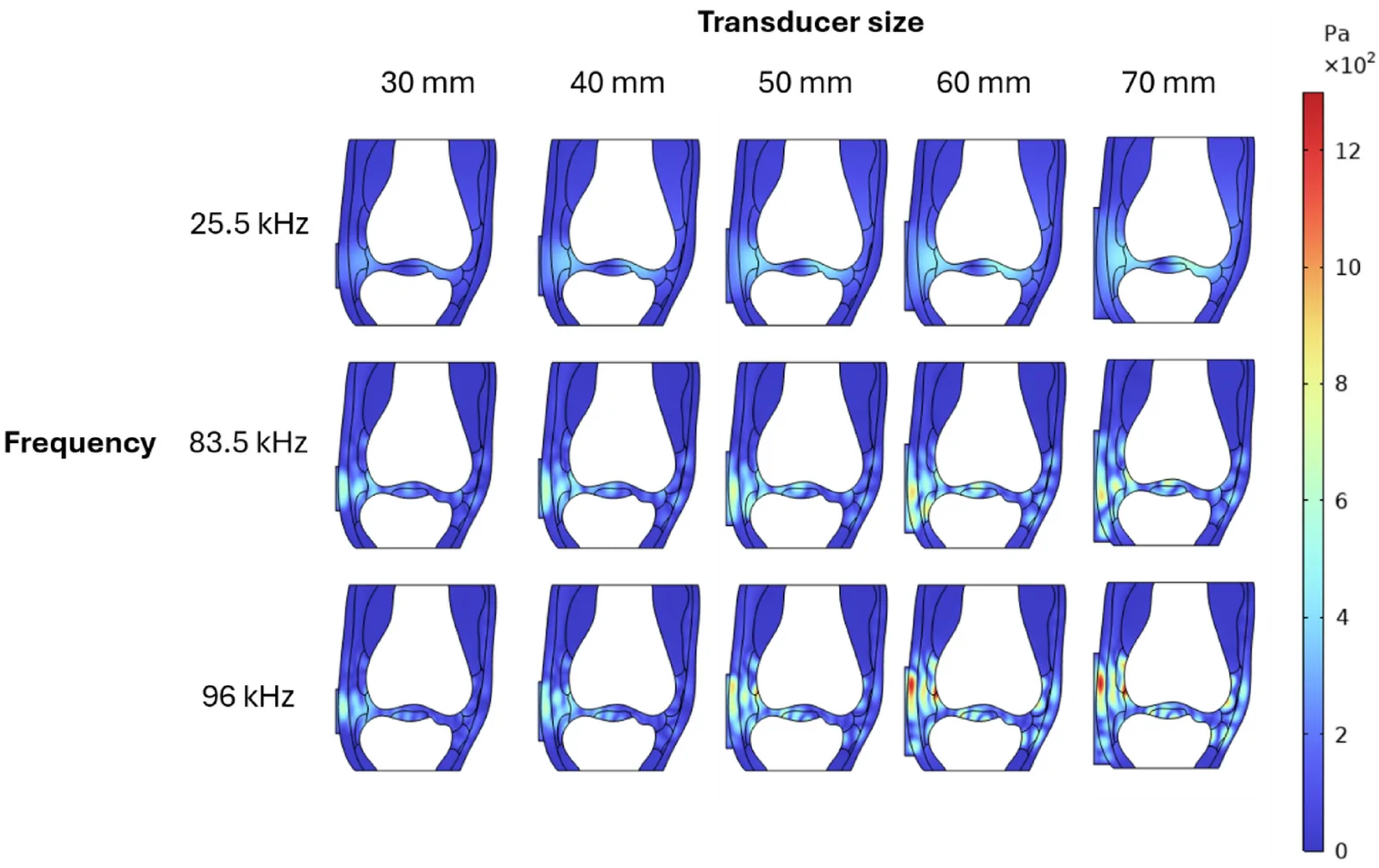
RMS 2D acoustic pressure distribution for Patient A under varying excitation conditions. The colour scale represents pressure amplitude

**Fig. 20:**
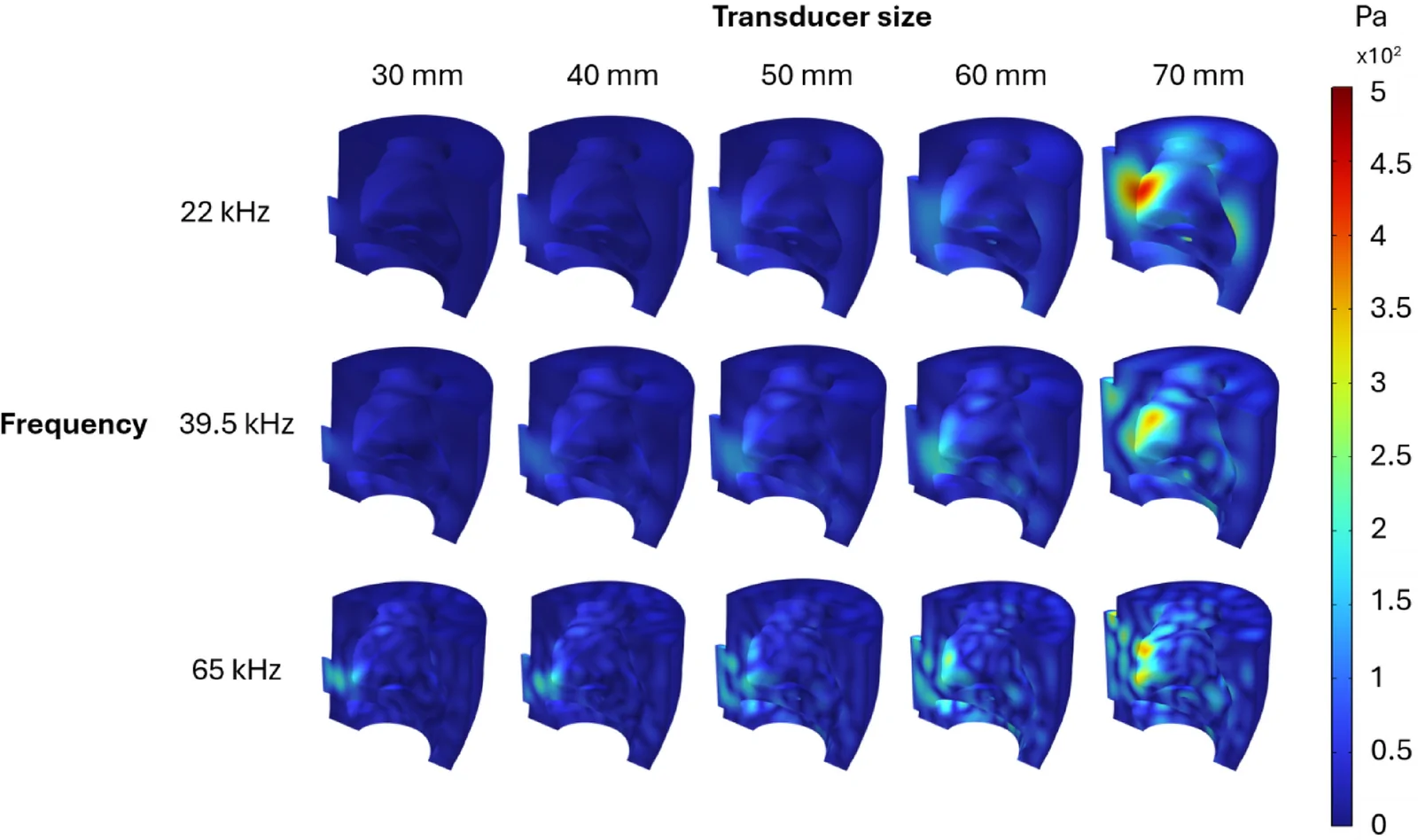
RMS 3D acoustic pressure distribution for Patient A under varying excitation conditions. The colour scale represents pressure amplitude

The RMS acoustic pressure distribution for Patient A (Figs. 19 and 20) illustrates the spatial propagation and attenuation of the wave field through the knee joint structures.

At lower excitation conditions, the acoustic field remains relatively diffuse, with modest pressure amplitudes primarily confined to the soft tissue layers and gradually decreasing towards the cartilage interfaces. The energy transmission appears broadly distributed, indicating that wave propagation is governed primarily by bulk tissue impedance and attenuation.

As the excitation parameters vary, localized amplification zones become more evident, particularly along tissue interfaces and near the cartilage surfaces. These regions of higher RMS pressure suggest constructive interference and impedance-related reflections at structural boundaries, especially at transitions between soft tissues and bone. The spatial concentration of pressure near the joint space indicates that the geometry of the knee plays a significant role in guiding wave energy towards cartilage.

While the overall propagation pattern remains consistent across the conditions shown, the magnitude and localization of peak pressures increase in specific configurations. This indicates that certain parameter combinations enhance energy focussing within the joint cavity. Nevertheless, the general propagation pathway remains structurally preserved, suggesting that the wave transmission mechanism is robust with respect to moderate variations in excitation parameters.

The RMS maps confirm that acoustic energy reaches the cartilage region with spatially heterogeneous distribution, influenced by tissue interfaces and geometric confinement. These results support the transmissibility findings in the frequency-domain analysis, demonstrating that the macro-scale propagation pathway can effectively deliver acoustic energy to the cartilage while exhibiting localized amplification effects governed by anatomical structure.

The 2D and 3D models produced qualitatively different acoustic fields. In the 2D representation, the pressure distribution was comparatively smooth and spatially confined, with energy concentrated around the joint space and along selected tissue interfaces. The highest local values approached $1.3\bullet {10}^{3}$ Pa. By contrast, the 3D model exhibited a more heterogeneous field characterized by multiple spatially distributed regions of constructive and destructive interference. Its maximum pressure was lower, reaching approximately $5.0\bullet {10}^{2}$ Pa.

The greater amplitudes obtained in 2D can be attributed primarily to dimensional confinement. Because out-of-plane propagation is excluded, the acoustic energy remains restricted to the modelling plane, leading to stronger superposition of incident and reflected waves. The 3D geometry permits lateral and out-of-plane spreading, diffraction, and interaction with a larger set of anatomical interfaces. These mechanisms redistribute acoustic energy throughout the joint and reduce the localized amplitudes observed in any individual section.

The maximum pressures identified in both acoustic fields should not be interpreted as the pressure effectively delivered to the articular cartilage. The global maxima occurred within localized regions of acoustic-wave trapping, where repeated reflections and constructive interference caused pressure accumulation between tissue boundaries or within confined anatomical regions. These hotspots therefore describe local features of the overall propagation field rather than direct acoustic impingement on the cartilage surface. Evaluation of cartilage exposure should instead rely on pressure values sampled or spatially integrated specifically within the cartilage domain.

**Fig. 21:**
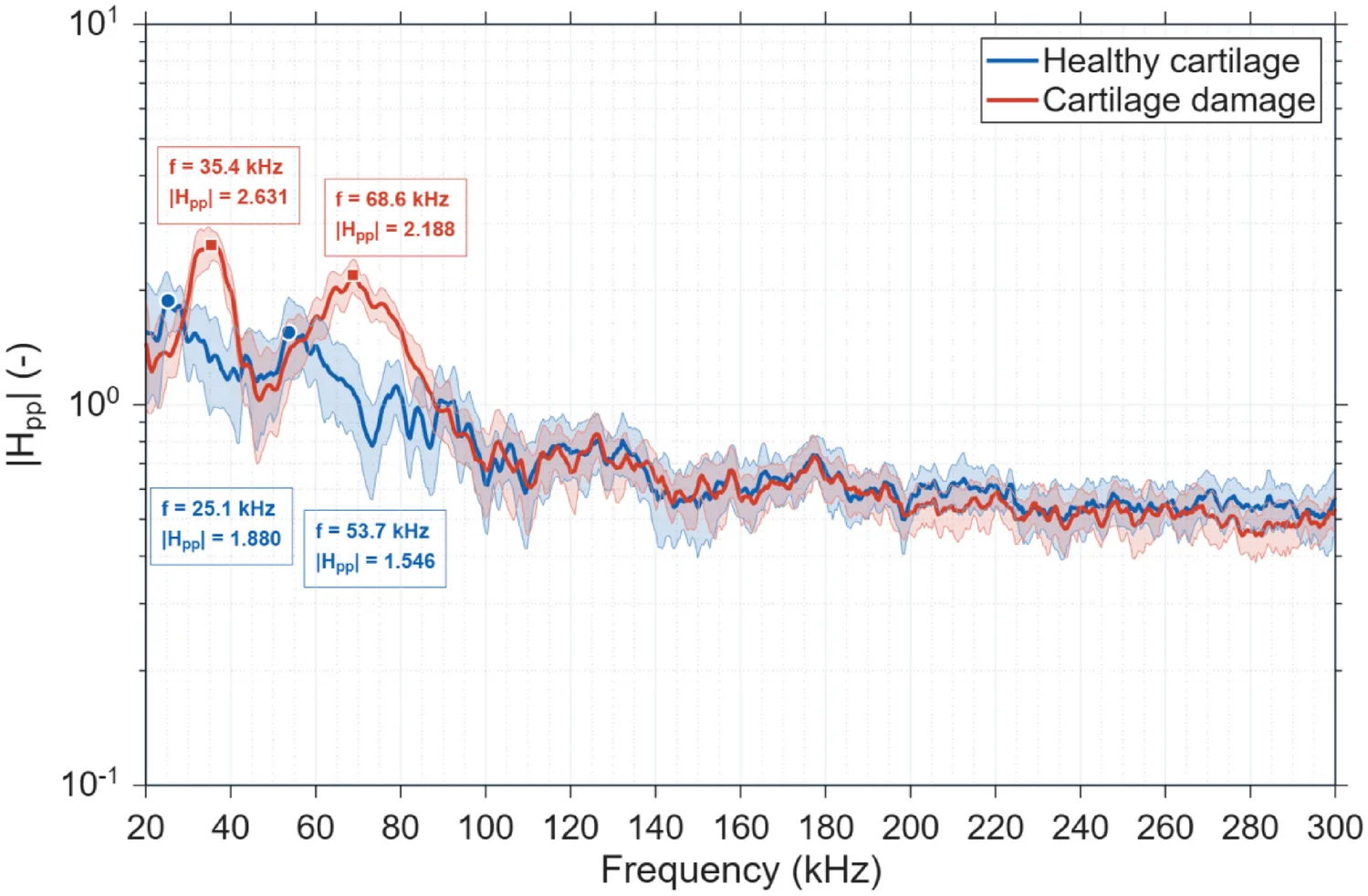
Average transfer function magnitude $\mid {H}_{pp}\mid$ to cartilage across all patients as a function of frequency from the 3d models. The solid line represents the mean response, and the shaded region indicates inter-patient variability

The average transfer function of all patients (Fig. 21) exhibits a clear frequency-dependent decay trend across the studied range.

The frequency–response functions reveal distinct propagation behaviour in healthy and damaged cartilage, particularly below 100 kHz. Healthy cartilage exhibited its highest transmissibility at 25.1 kHz, with $\mid {H}_{pp}\mid =1.880$, followed by a secondary maximum of 1.546 at 53.7 kHz. In damaged cartilage, the two principal peaks shifted to higher frequencies and increased in magnitude, reaching 2.631 at 35.4 kHz and 2.188 at 68.6 kHz. Cartilage damage therefore produced stronger local pressure amplification within the low-frequency region and displaced the dominant response features by approximately 10–15 kHz relative to the healthy condition.

The greatest inter-case variability occurred below approximately 100 kHz, as indicated by the broader shaded regions surrounding both mean curves. This dispersion was particularly evident around the principal peaks and suggests that low-frequency pressure transmission is more sensitive to variations in cartilage geometry, material properties, and surrounding anatomical structures. Although the response of damaged cartilage exceeded that of healthy tissue around 30–40 and 60–80 kHz, the curves intersected at several intermediate frequencies. Consequently, the influence of cartilage condition was frequency dependent rather than a uniform amplification across the entire spectrum.

Above approximately 100 kHz, both transfer functions decreased below unity and became progressively more similar. The responses exhibited moderate local fluctuations between 100 and 190 kHz, followed by a comparatively stable region at higher frequencies. Between approximately 200 and 300 kHz, $\mid {H}_{pp}\mid$ generally remained within the range of about 0.45–0.65, with the damaged-cartilage response tending to be slightly lower than that of healthy tissue. The narrower separation and overlapping variability bands in this region indicate reduced sensitivity to cartilage condition at higher frequencies. Overall, the results suggest that cartilage damage predominantly modifies low-frequency acoustic transmissibility by increasing and shifting the principal amplification peaks, whereas the high-frequency response is more strongly attenuated and less dependent on tissue condition.

The localized pressure amplification factor, ${H}_{pp}$, should be interpreted as an acoustic transmission metric rather than a direct measure of structural deformation. Although elevated ${H}_{pp}$ values indicate stronger local acoustic pressure within the cartilage region, hydrostatic pressure amplification in nearly incompressible biological tissues does not necessarily produce proportional deviatoric strain. The conversion of acoustic pressure into cellular or tissue-level deformation depends on local geometry, impedance contrasts, boundary conditions, material compressibility, damping, and the resulting displacement field. Therefore, ${H}_{pp}$ was used to identify frequency bands associated with enhanced acoustic exposure of the cartilage, while structural strain and mechanotransductive relevance were assessed separately from the corresponding strain-based microscale response.

### Multi-scale coupled results

#### Coupled frequency response

**Fig. 22:**
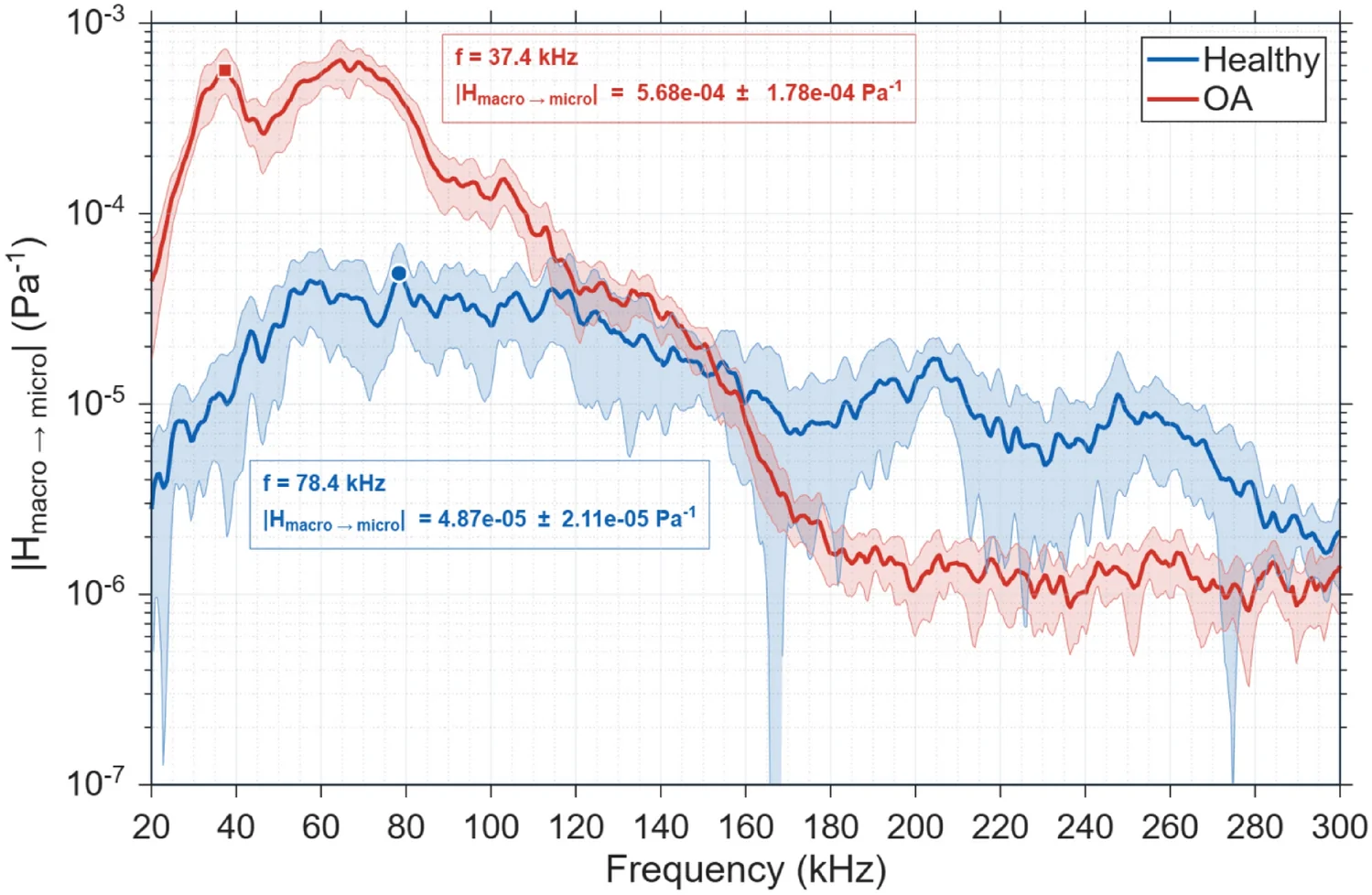
Multi-scale coupled transfer function $|H_{{{\mathrm{macro}} \to {\mathrm{micro}}}} |$ as a function of frequency for healthy and diseased patients. The solid line represents the mean response across patients, and the shaded region indicates inter-patient variability

The multi-scale coupled transfer function results reveal distinct frequency-dependent behaviour between healthy and OA chondrocytes (Fig. 22), highlighting how cellular alterations modulate pressure-to-strain transmission from the macro-scale knee model to the nuclear level.

For healthy chondrocytes, the macro-to-micro transfer function exhibits a broad amplification region extending from 50–170 kHz. The identified maximum occurs at 78.4 kHz, with a mean magnitude of $\mid {H}_{\mathrm{m}\mathrm{a}\mathrm{c}\mathrm{r}\mathrm{o}\to \mathrm{m}\mathrm{i}\mathrm{c}\mathrm{r}\mathrm{o}}\mid =(4.87\pm 2.11)\times {10}^{-5} {\mathrm{P}\mathrm{a}}^{-1}$. The relatively wide variability band in this region indicates sensitivity to patient-specific anatomy and cellular geometry. Beyond 170 kHz, the response gradually decreases, although secondary amplification regions remain visible near 210 and 255 kHz. Healthy chondrocytes therefore retain measurable macro-to-micro coupling across a comparatively broad frequency spectrum.

The OA configuration displays substantially greater transmissibility at low frequencies. A prominent peak is observed at 37.4 kHz, reaching $\mid {H}_{\mathrm{m}\mathrm{a}\mathrm{c}\mathrm{r}\mathrm{o}\to \mathrm{m}\mathrm{i}\mathrm{c}\mathrm{r}\mathrm{o}}\mid =(5.68\pm 1.78)\times {10}^{-4} {\mathrm{P}\mathrm{a}}^{-1}$, one order of magnitude greater than the maximum identified for healthy chondrocytes. The elevated response extends broadly from approximately 30–85 kHz, with additional amplification in 55–80 kHz. Above 85 kHz, the transfer function declines rapidly and approaches the healthy response between 120 and 160 kHz. Beyond 170 kHz, the OA curve stabilizes near ${10}^{-6} {\mathrm{P}\mathrm{a}}^{-1}$, indicating limited high-frequency transmission to the nucleus.

The multi-scale frequency response demonstrate that disease state substantially alters the frequency at which acoustic energy is most efficiently converted into nuclear strain. While healthy cartilage exhibits a mid-frequency amplification regime, osteoarthritic tissue shows enhanced sensitivity primarily at lower frequencies, implying that optimal stimulation strategies may differ between physiological and pathological conditions.

#### Coupled time-explicit response

**Fig. 23:**
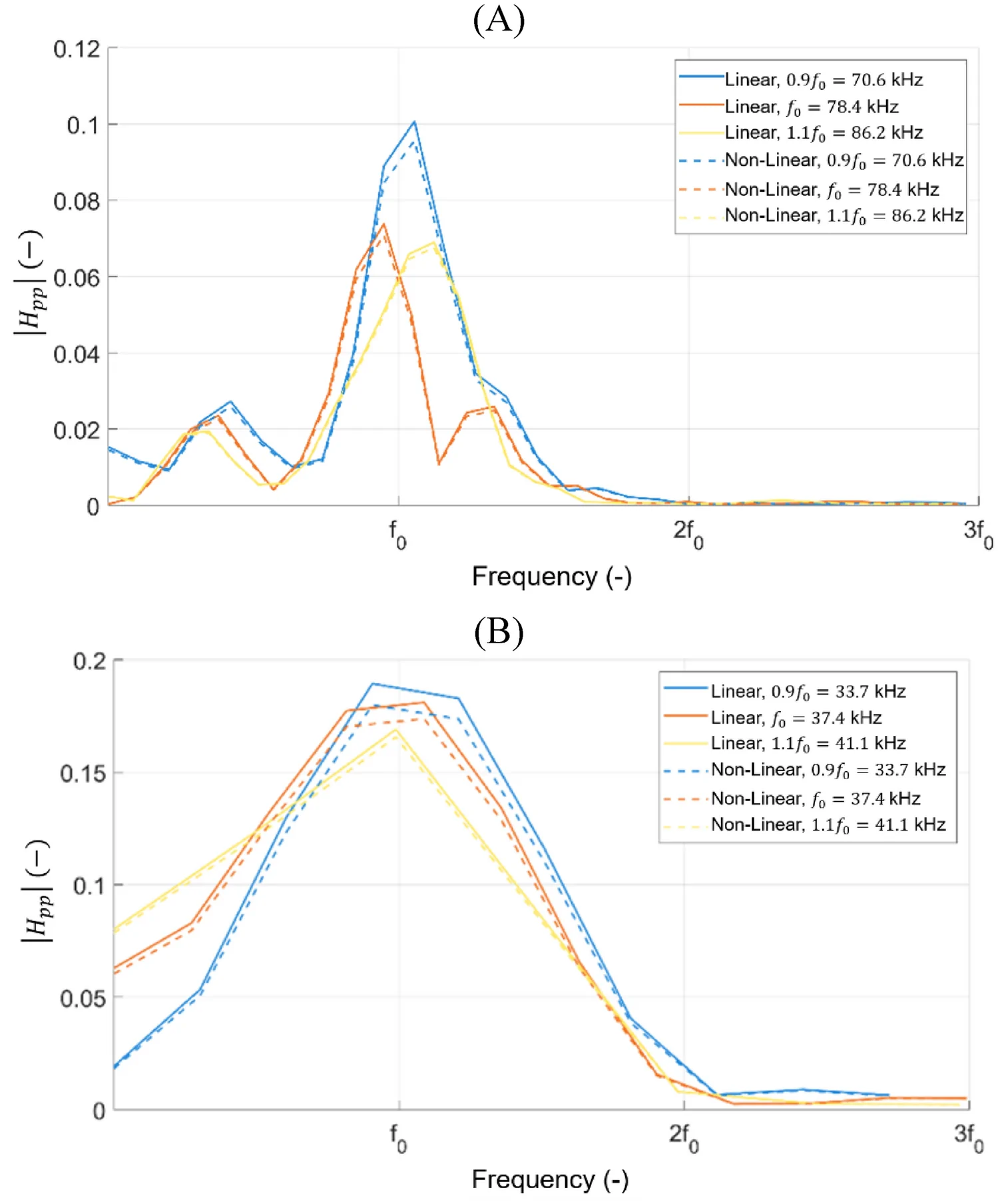
Time-explicit spectral response under linear and nonlinear acoustic propagation for excitation frequencies $0.9{f}_{0}$, ${f}_{0}$, and $1.1{f}_{0}$ for **A** healthy patient A and **B** patient A with OA

Time-explicit simulations were used to assess whether finite-amplitude nonlinear propagation materially alters the response at the stimulation conditions identified as optimal in the frequency-domain analysis (Fig. 23). For each case, three closely spaced excitation frequencies were selected ($0.9{f}_{0}$, ${f}_{0}$, and $1.1{f}_{0}$) to probe sensitivity around the target operating point, and the resulting spectra were compared between linear and nonlinear acoustic formulations.

In both the healthy configuration (Fig. 39 A, ${f}_{0}$) and the OA configuration (Fig. 39 B, ${f}_{0}$), the response is dominated by a strong component at the fundamental excitation frequency, with only minimal energy observed at higher multiples. Importantly, the nonlinear simulations do not exhibit appreciable spectral growth at the second or third harmonics (near $2{f}_{0}$ and $3{f}_{0}$), and the spectral shapes remain closely aligned with the linear results for all three excitation frequencies. This indicates that, under the applied pressure amplitudes and propagation distances considered, waveform steepening and harmonic generation (hallmarks of nonlinear acoustics) are negligible. Consequently, the observed transmission behaviour is well captured by the linear acoustic model, and inclusion of nonlinear terms does not provide a meaningful change in predicted response at the operating conditions relevant for mechanotransductive stimulation.

The results also show that the system is sensitive to excitation frequency within a narrow neighbourhood of ${f}_{0}$, particularly in the healthy case where the response peaks sharply around the fundamental and drops more rapidly for ${0.9f}_{0}$ and ${1.1f}_{0}$. This behaviour is consistent with a resonance-driven amplification mechanism previously identified in the harmonic FRF analysis. In contrast, the OA case displays a broader spectral peak around the fundamental, suggesting a less sharply tuned response, which is consistent with increased damping and mechanical heterogeneity inferred from OA material properties and geometry. Across both phenotypes, however, the near-overlap between linear and nonlinear spectra confirms that the dominant determinant of the response is frequency-dependent transmissibility rather than nonlinear propagation.

**Fig. 24:**
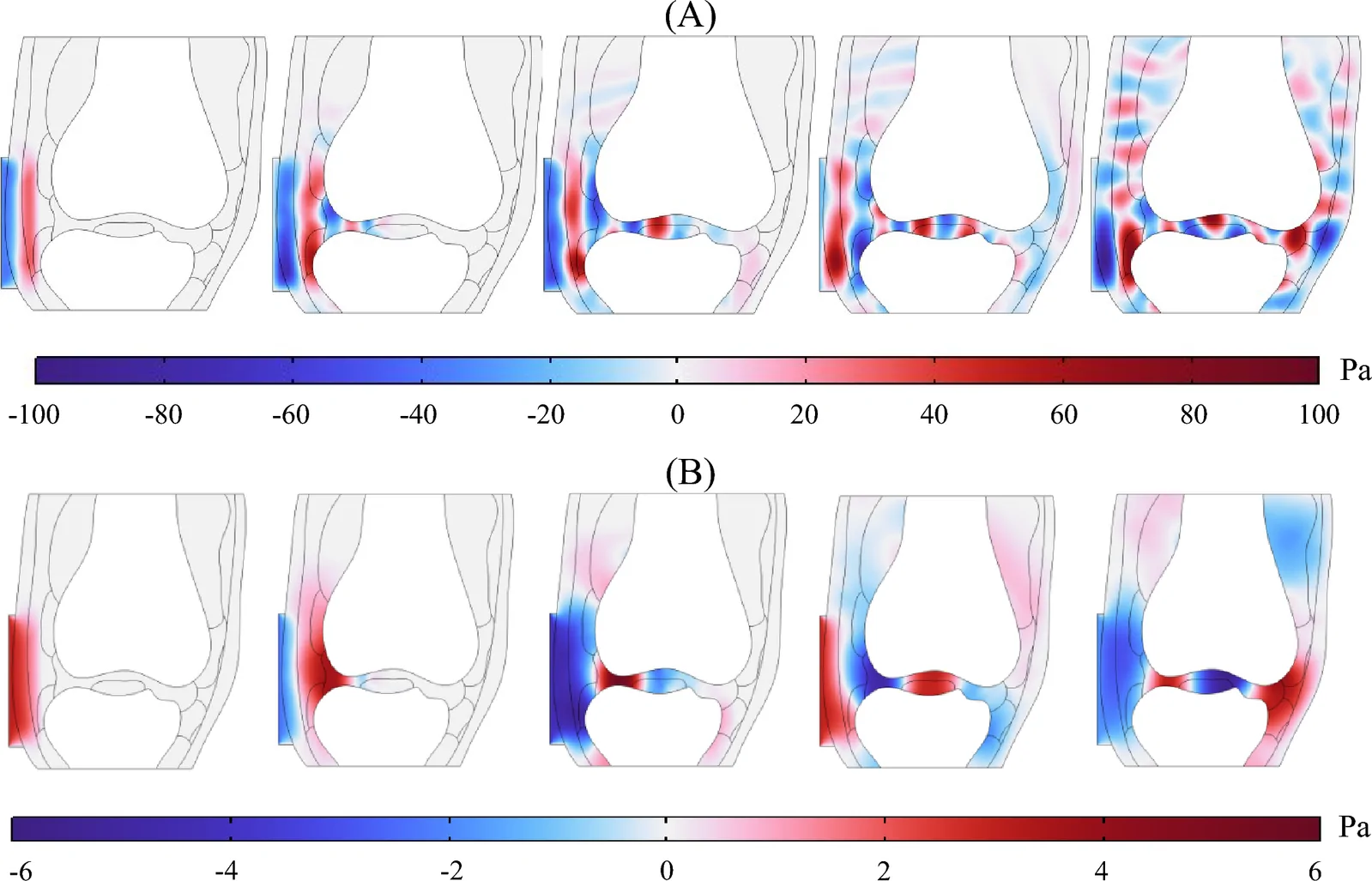
Time-explicit acoustic pressure field showing wave propagation across the knee joint for patient A using ideal ${f}_{0}$ for **A** Healthy Patients and **B** OA Patients. The colour scale represents instantaneous pressure (Pa)

**Fig. 26:**
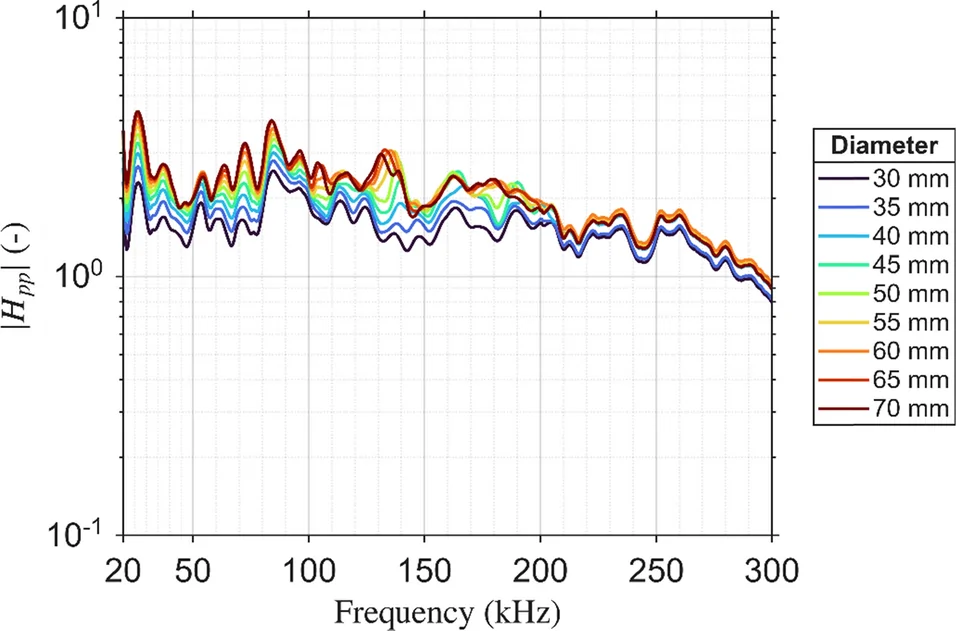
2D Transfer function magnitude ${|H}_{pp}|$ ​from transducer to cartilage for Patient A as a function of frequency for varying transducer diameters (30–70 mm)

**Fig. 27:**
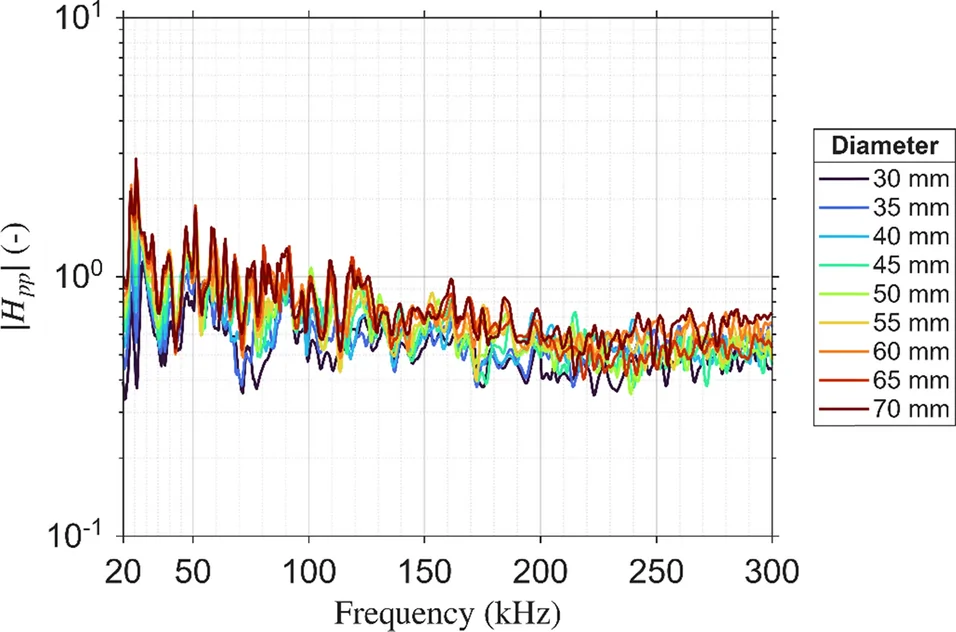
2D Transfer function magnitude ${|H}_{pp}|$ ​from transducer to cartilage for Patient B as a function of frequency for varying transducer diameters (30–70 mm)

**Fig. 28:**
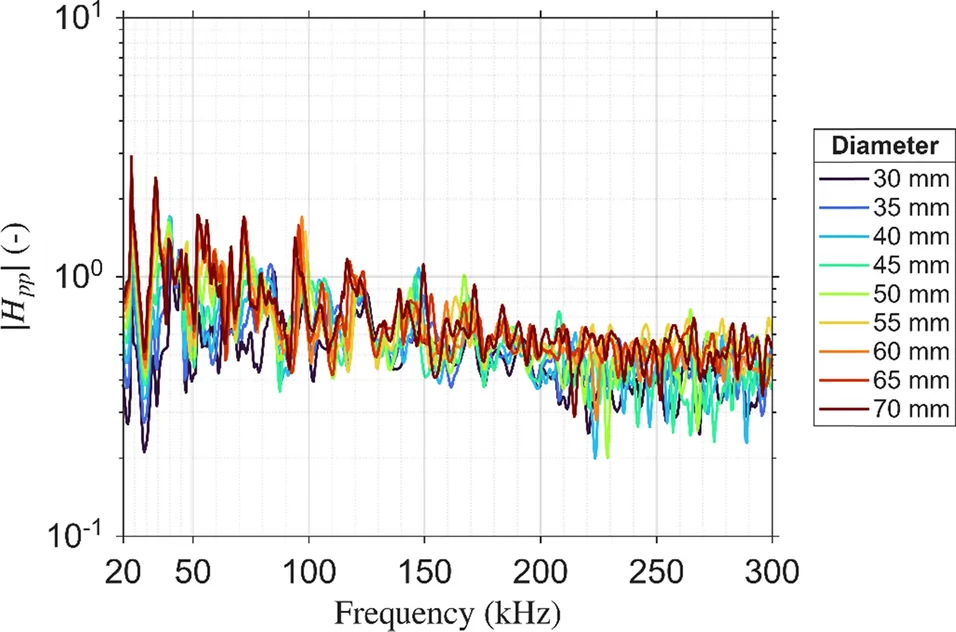
2D Transfer function magnitude ${|H}_{pp}|$ ​from transducer to cartilage for Patient C as a function of frequency for varying transducer diameters (30–70 mm)

**Fig. 29:**
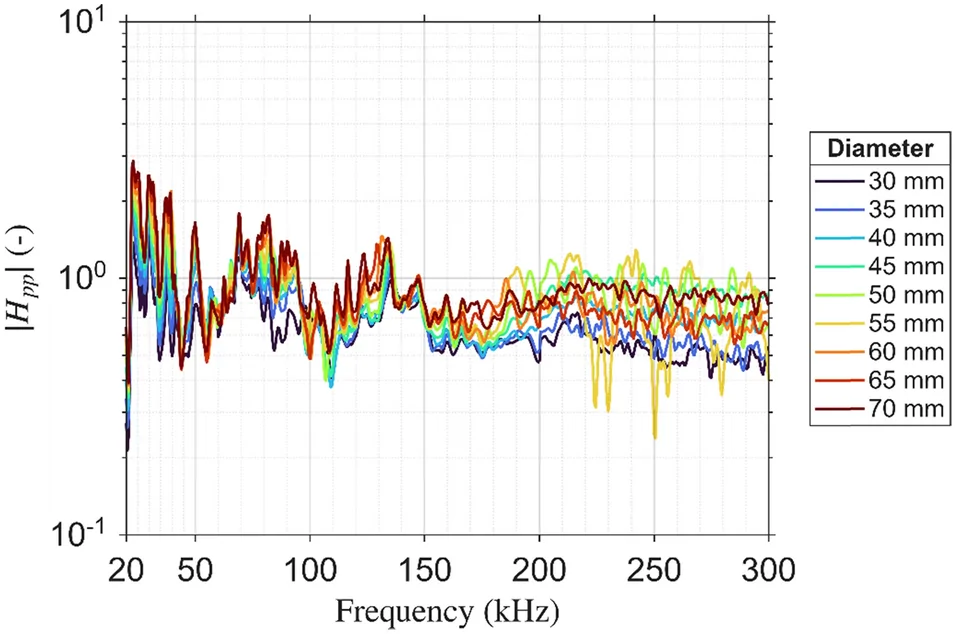
2D Transfer function magnitude ${|H}_{pp}|$ ​from transducer to cartilage for Patient D as a function of frequency for varying transducer diameters (30–70 mm)

**Fig. 30:**
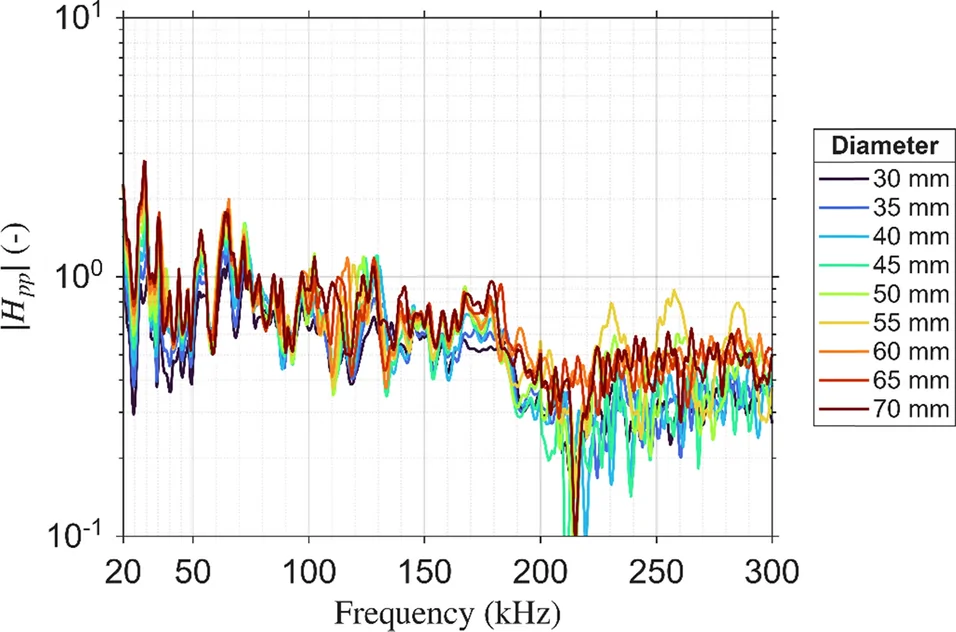
2D Transfer function magnitude ${|H}_{pp}|$ ​from transducer to cartilage for Patient E as a function of frequency for varying transducer diameters (30–70 mm)

**Fig. 31:**
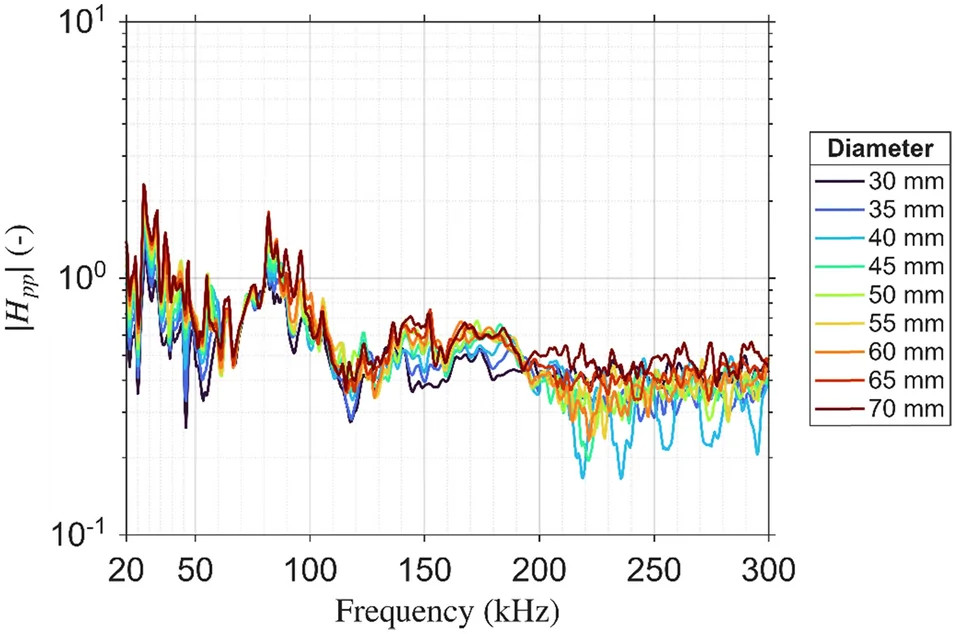
2D Transfer function magnitude ${|H}_{pp}|$ ​from transducer to cartilage for Patient F as a function of frequency for varying transducer diameters (30–70 mm)

**Fig. 32:**
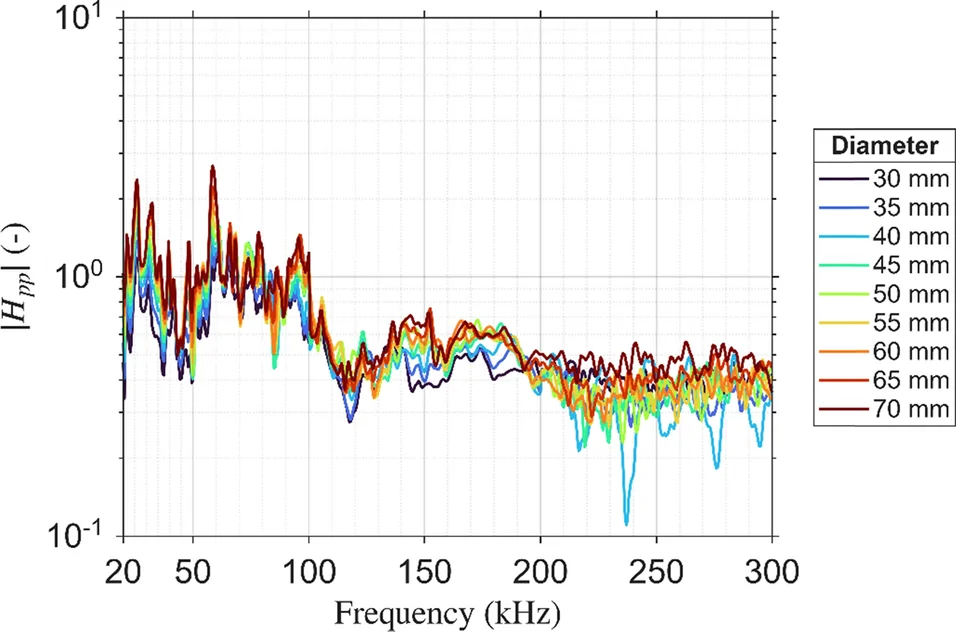
2D Transfer function magnitude ${|H}_{pp}|$ ​from transducer to cartilage for Patient G as a function of frequency for varying transducer diameters (30–70 mm)

**Fig. 33:**
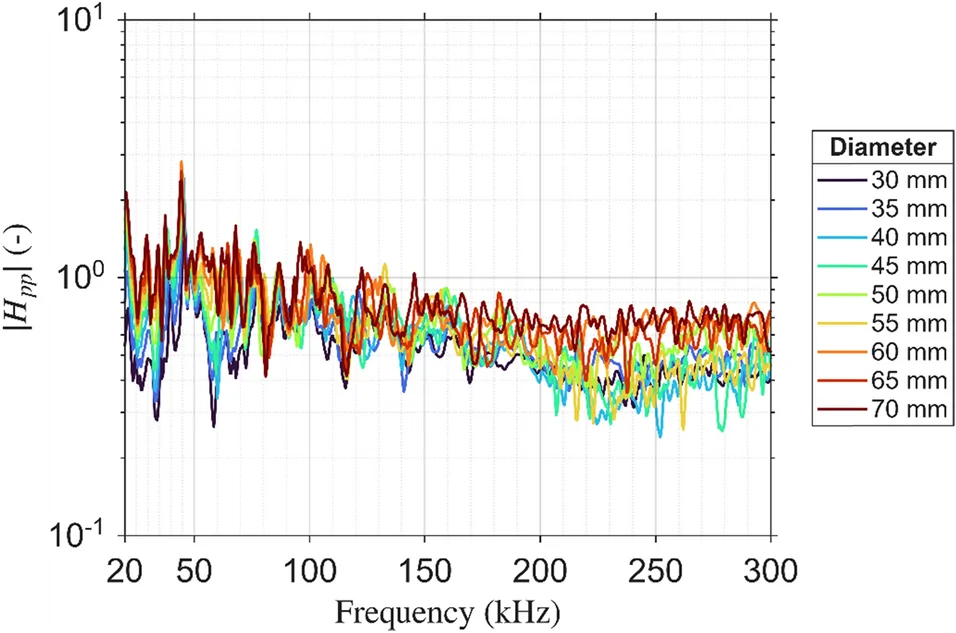
2D Transfer function magnitude ${|H}_{pp}|$ ​from transducer to cartilage for Patient H as a function of frequency for varying transducer diameters (30–70 mm)

**Fig. 34:**
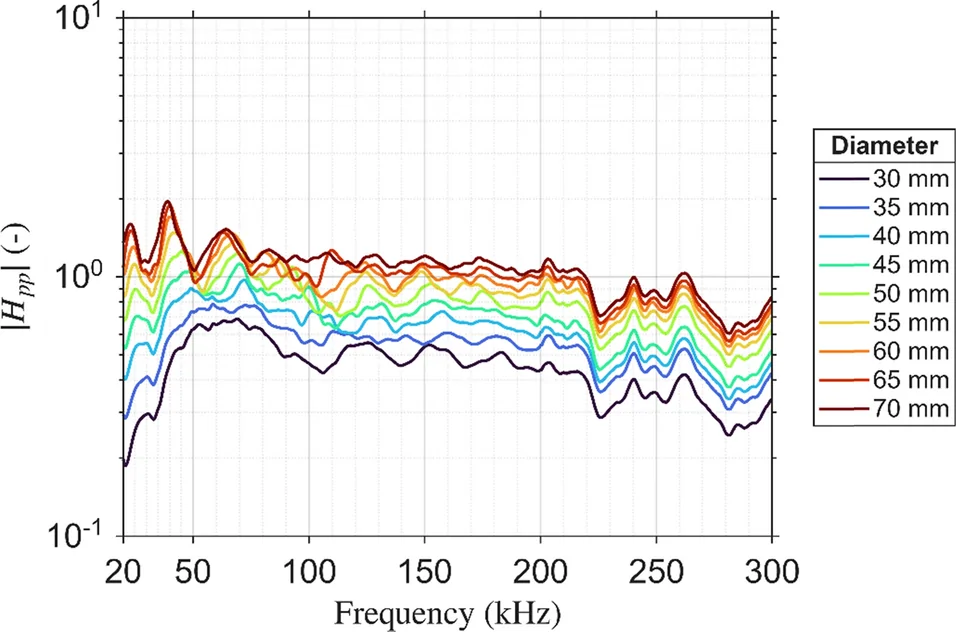
3D Transfer function magnitude ${|H}_{pp}|$ ​from transducer to cartilage for Patient A as a function of frequency for varying transducer diameters (30–70 mm)

**Fig. 35:**
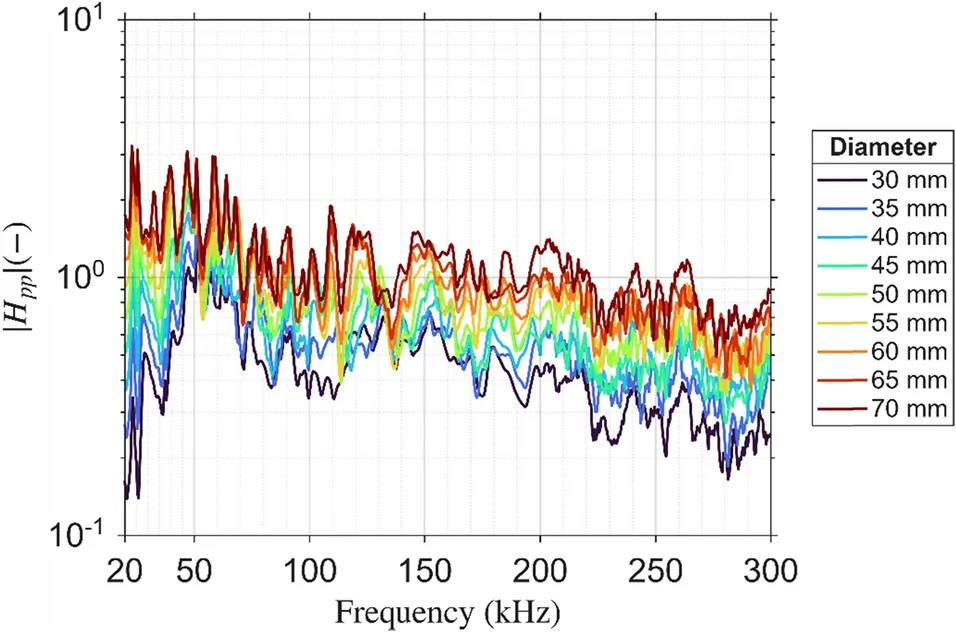
3D Transfer function magnitude ${|H}_{pp}|$ ​from transducer to cartilage for Patient B as a function of frequency for varying transducer diameters (30–70 mm)

**Fig. 36:**
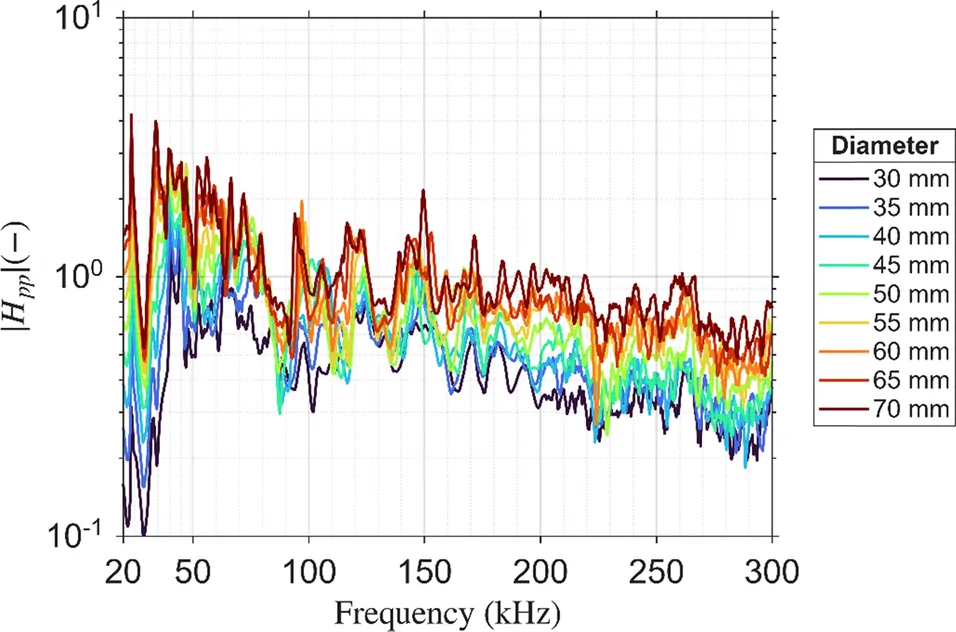
3D Transfer function magnitude ${|H}_{pp}|$ from transducer to cartilage for Patient C as a function of frequency for varying transducer diameters (30–70 mm)

**Fig. 37:**
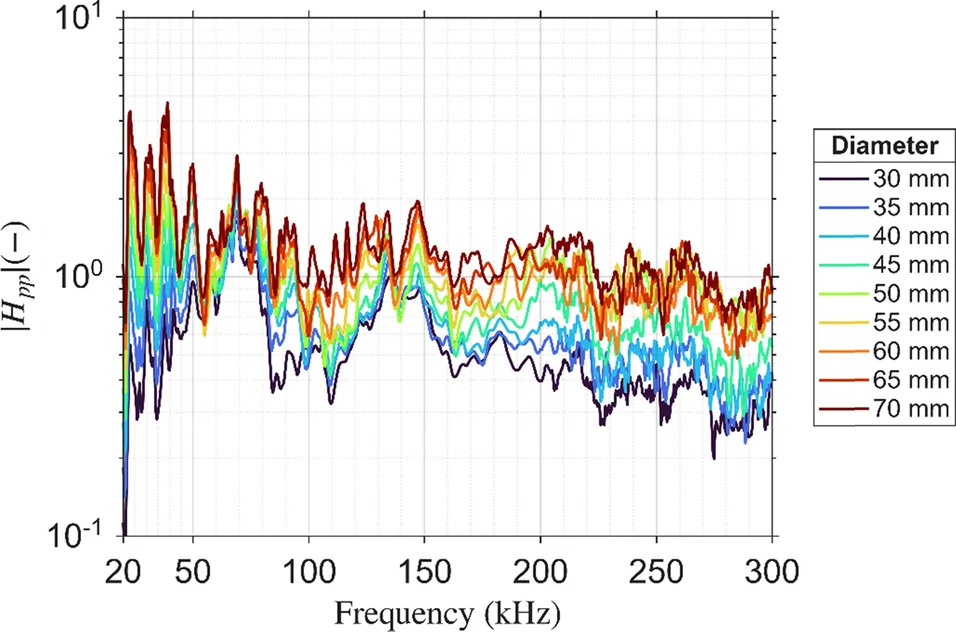
3D Transfer function magnitude ${|H}_{pp}|$ from transducer to cartilage for Patient D as a function of frequency for varying transducer diameters (30–70 mm)

**Fig. 38:**
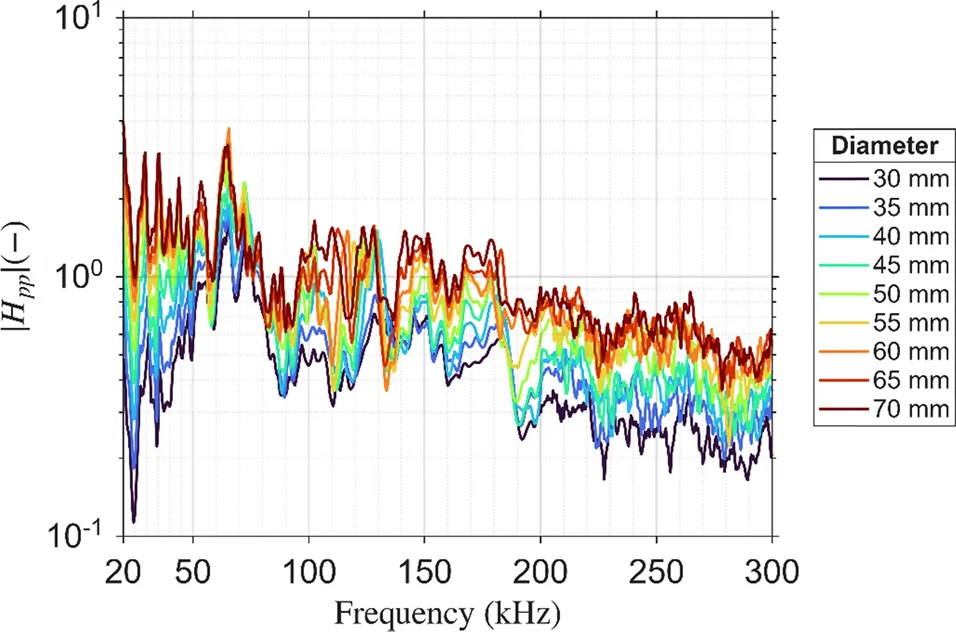
3D Transfer function magnitude ${|H}_{pp}|$ from transducer to cartilage for Patient E as a function of frequency for varying transducer diameters (30–70 mm)

**Fig. 39:**
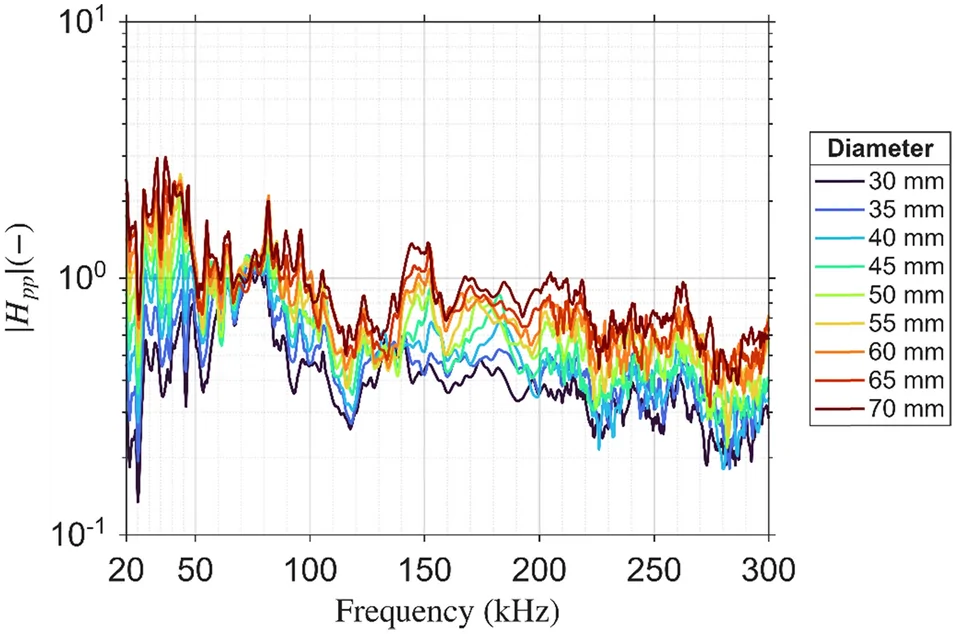
3D Transfer function magnitude ${|H}_{pp}|$ from transducer to cartilage for Patient F as a function of frequency for varying transducer diameters (30–70 mm)

**Fig. 40:**
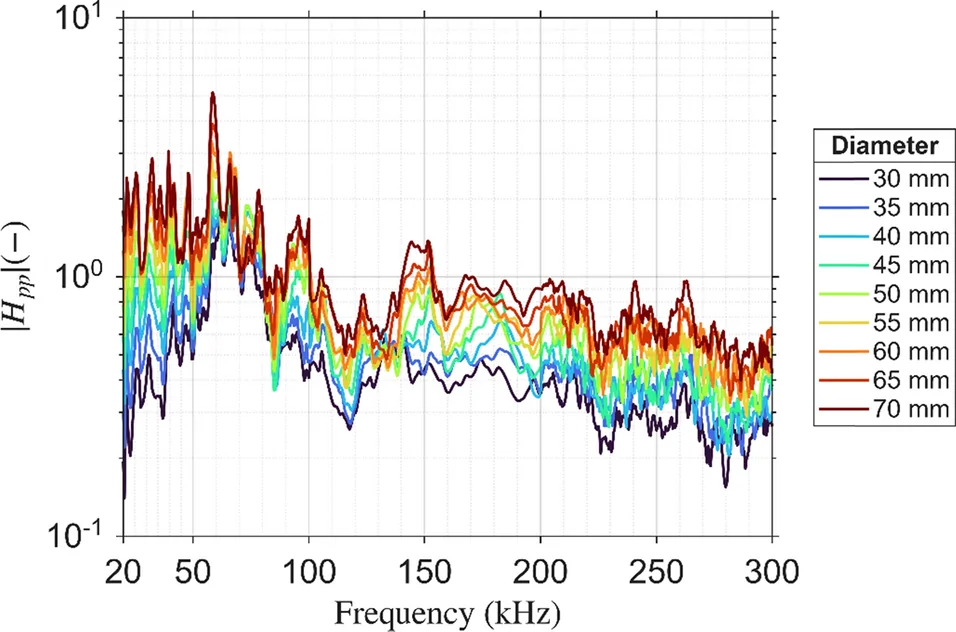
3D Transfer function magnitude ${|H}_{pp}|$ from transducer to cartilage for Patient G as a function of frequency for varying transducer diameters (30–70 mm)

**Fig. 41:**
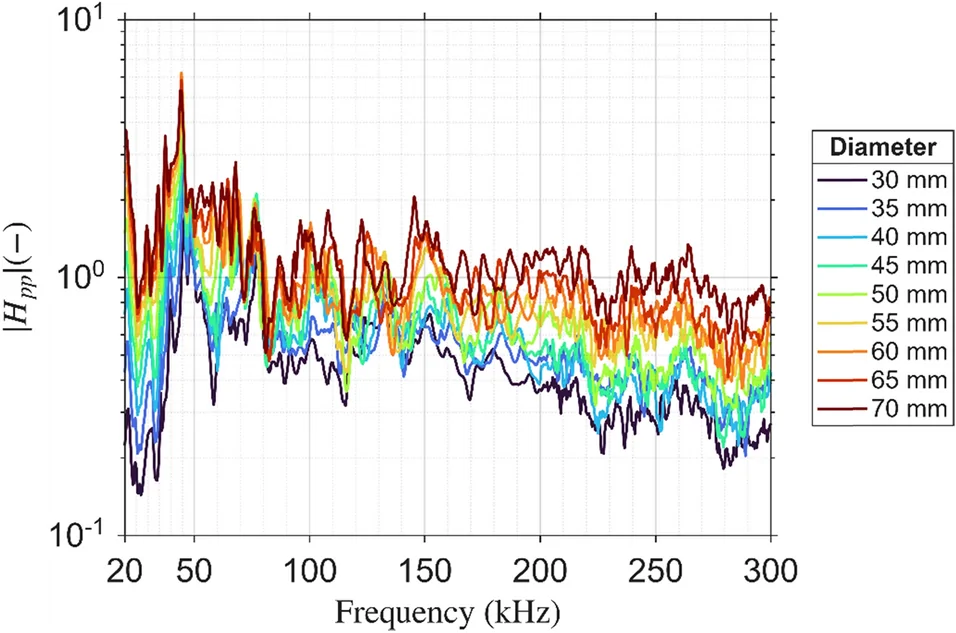
3D Transfer function magnitude ${|H}_{pp}|$ from transducer to cartilage for Patient H as a function of frequency for varying transducer diameters (30–70 mm)

The pressure field in Fig. 24 illustrates the transient time-explicit propagation of the acoustic wave through the layered knee structures for the selected excitation conditions. In both configurations, the wave originates at the lateral soft-tissue boundary and progresses medially towards the joint space, interacting with tissue interfaces and impedance discontinuities along its path.

In the healthy configuration (Fig. 24 A), the pressure amplitudes are higher, and the wave pattern shows pronounced oscillatory behaviour as it traverses muscle, adipose tissue, and cartilage. Clear alternating compressive and rarefactive regions develop within the joint cavity, indicating strong wave reflection and -interference between cartilage surfaces and adjacent bone. The spatial distribution suggests partial confinement of acoustic energy within the joint space, leading to localized amplification along cartilage interfaces. The interference pattern becomes increasingly structured as the wave penetrates deeper, consistent with resonance-driven transmission previously identified in the frequency-domain analysis.

In contrast, the OA configuration (Fig. 24 B) exhibits significantly lower pressure amplitudes and a more diffuse wave pattern. The oscillatory structure within the joint space is less pronounced, and pressure magnitudes attenuate more rapidly as the wave propagates across the knee. This behaviour indicates reduced constructive interference and weaker energy confinement within the cartilage region. The lower amplitude and smoother spatial gradients are consistent with the altered mechanical properties and damping characteristics associated with OA tissue, which shift transmissibility towards lower frequencies and suppress higher-frequency amplification.

Across both cases, the wave clearly propagates through superficial tissues before reaching the joint cavity, demonstrating that macro-scale excitation can effectively transmit energy to cartilage. However, the spatial organization and magnitude of the pressure field depend strongly on the underlying tissue mechanics. The healthy knee supports more structured standing-wave–like patterns within the joint, whereas the OA knee shows a more attenuated and less resonant response. These findings align with the multi-scale transfer function results, confirming that disease-related mechanical changes modify not only frequency-domain transmissibility but also the spatial distribution of acoustic energy within the knee.

## Discussion

The findings in this study demonstrate that acoustic stimulation of cartilage is frequency-band dependent, disease-sensitive (because it will mechanically alter the condition of the chondrocytes (Nahian and Sapra 2025; Chawla et al. 2022)), and predominantly linear under clinically relevant pressures.

At the cellular scale, modal analysis demonstrated that chondrocytes exhibit spheroidal and torsional vibration modes analogous to elastic sphere behaviour. Among these, the ${s}_{3}$​ spheroidal mode produced the most significant nuclear deformation pattern, making it the primary mechanotransductive target. Parametric analyses showed that chondrocyte radius is the dominant geometric factor governing modal frequency shifts, while PCM thickness has a secondary effect and plasma membrane thickness and intercellular spacing have negligible influence. In OA cells, modal frequencies shift downward due to hypertrophy and altered stiffness, confirming disease-dependent dynamic behaviour. Although the OA nucleus was assigned a higher elastic modulus, the corresponding reduction in resonance frequency does not contradict the classical stiffness–frequency relationship. The chondrocyte is a coupled multilayer system, and its natural frequencies are governed by the global eigenvalue problem, rather than by the stiffness of an individual cellular component. In the OA model, the decrease in cytoplasmic stiffness, combined with the substantial increase in the relative volume of the cytoplasm, reduces the effective stiffness of the dominant coupled modes. This effect outweighs the localized increase in nuclear stiffness, leading to lower predicted natural frequencies. Frequency–response analysis revealed that nuclear strain amplification occurs within specific frequency bands rather than at a single discrete frequency. Healthy chondrocytes exhibited a broader mid-frequency amplification band, whereas OA cells showed a dominant low-frequency peak with reduced high-frequency sensitivity.

At the macroscale, acoustic wave propagation through the layered knee tissues demonstrated that energy reaches the cartilage with spatially heterogeneous distribution governed by impedance mismatches and geometric confinement. Beyond a certain transducer diameter, transmissibility stabilizes, indicating limited sensitivity to source placement. Despite the amplitude differences, both formulations (2D and 3D) confirmed that acoustic energy reached the joint region. The 3D results provide a more physically complete representation of this process by accounting for energy redistribution in all spatial directions. As such, the 2D model remains useful for identifying general propagation tendencies and conducting broad parametric analyses, but it may overestimate localized pressures and cannot fully reproduce the complex interference patterns present in the three-dimensional anatomy.

Multi-scale coupling of macro-scale pressure to micro-scale nuclear strain confirmed that disease state significantly alters pressure-to-strain transmissibility. Healthy cartilage supports broader and higher-frequency amplification (50–170 kHz), whereas OA tissue concentrates transmissibility in a narrower low-frequency band (30–85 kHz). The comparison demonstrates that OA-related alterations enhance and shift macro-to-micro coupling towards lower frequencies while simultaneously narrowing the frequency range over which substantial nuclear deformation is maintained. Cellular hypertrophy, PCM softening, and changes in the mechanical properties of the intracellular components may collectively reduce the effective dynamic stiffness of the chondrocyte–PCM system, thereby increasing its low-frequency compliance. Conversely, the broader response of healthy chondrocytes suggests more distributed modal participation and greater preservation of higher-frequency coupling. The predicted differences remain dependent on the adopted geometrical and constitutive assumptions and should therefore be interpreted as model-based trends pending experimental validation.

Time-explicit simulations comparing linear and nonlinear acoustic formulations demonstrated negligible harmonic generation and no significant nonlinear spectral growth at the applied pressure levels. This indicates that, within the mechanotransductive strain range considered, wave propagation is adequately described by linear acoustics.

The ranges reported in this study are markedly different from the stimulation frequencies typically employed in the cartilage and OA literature. Conventional therapeutic ultrasound therapies (continuous or pulsed) typically have a representative carrier frequency of 1–3 MHz (Matthews and Stretanski 2023). For continuous therapeutic ultrasound RCTs report that the frequency used across is 1 MHz, with intensities commonly in the 1–2 W/cm^2^ range and session times ~5–10 min (Dantas et al. 2021). Long-duration ultrasound treatment can also be applied at 3 MHz at 132 mW/cm^2^ for 4 h daily (Winkler et al. 2021). Regarding Low-Intensity Pulsed Ultrasound (LIPUS), Miller et al. note that the vast majority of in vitro and in vivo cartilage restoration protocols rely on empirically derived LIPUS regimens at 1.0–1.5 MHz without explicit tuning to cellular resonances (Miller et al. 2017), and similarly Zhou et al. describe standard LIPUS devices operating at 1–3 MHz, most commonly around 1.5 MHz (Zhou et al. 2024). Similarly, Sekino et al. apply pulsed ultrasound at 1.5 MHz with intensities <100 mW/cm^2^ (Sekino et al. 2018). For cartilage-directed approaches, a randomised trial of focussed LIPUS (FLIPUS) for knee OA reported a 0.6 MHz carrier frequency (600 kHz) with a low-intensity pulsed regime and described this as enabling more direct cartilage stimulation than earlier unfocussed approaches (Jia et al. 2022).

In fact, the chosen frequencies for chondrocyte stimulation directly contradict general therapeutic ultrasound teaching: higher-frequency ultrasound attenuates more rapidly and is commonly used for more superficial effects, while lower frequency should be used for deeper heating (Matthews and Stretanski 2023). This is in part corroborated by current findings in the literature. For conventional therapeutic ultrasound in knee OA (typically 1 MHz), a GRADE systematic review assessment found small, statistically significant improvements in pain versus sham controls, but judged the overall certainty of evidence as low, with substantial limitations across trials (Dantas et al. 2021). A large sham-controlled trial using LIPUS-type device (1.5 MHz carrier) reported no clinically meaningful improvement in symptoms or cartilage thickness over a lengthy treatment period (48 weeks) (Sawitzke et al. 2022). A comparative review of multiple major OA guidelines reported insufficient evidence to recommend therapeutic ultrasound (along with several other passive modalities), even while exercise, weight management strategies are more consistently supported across guidelines (Overton et al. 2022). These findings might also be partially explained due to the fact that authors often primarily report symptom-management claims rather than confirmed disease-modification, and the modality’s mechanism is commonly framed in the “deep heating/diathermy over hours” regime (Winkler et al. 2021), suggesting that high frequency protocols may be inherently inefficient at producing the nuclear deformation necessary.

On the other hand, vibration-based approaches for cartilage and OA typically use very low frequencies, either whole-body vibration in the 5–20 Hz range in animal OA models (Lützenberg et al. 2019; Wang et al. 2023), or direct mechanical vibration of chondrocytes at hundreds of hertz, with enhanced proliferation reported above 350 Hz but still well below kilohertz regime identified in our model (Kaupp and Waldman 2008). Taken together, this suggests that the predicted optimal bands of 30–85 kHz (OA) and 50–170 kHz (healthy) occupy an underexplored frequency region between classical low-frequency mechanical loading and conventional MHz LIPUS, and that future experimental work explicitly targeting these bands could test whether resonance-based stimulation at cell- and disease-specific frequencies yields more efficient mechanobiological responses than the empirically chosen frequencies currently used in the literature.

The suggested range is already applied in transdermal ultrasound/sonophoresis literature, with 20–200 kHz being a standard definition of “low frequency ultrasound”(Marathe et al. 2024). A clinical prototype for chronic wounds has been developed to operate in the 20–100 kHz range, explicitly aiming for non-thermal, non-inertial-cavitational exposure (with peak pressure limited and extended wearability) (Ngo et al. 2019).

This study presents a novel multi-scale computational framework linking macro-scale acoustic wave propagation in the knee to mechanotransductive strain at the level of the chondrocyte nucleus. A primary strength of the work is that it is, to our knowledge, the first study to explicitly relate ultrasound-based stimulation strategies to nuclear strain at the single-cell level within a patient-specific joint geometry. Although previously articles have previously studied the modal analysis and harmonic responses of individual cells (Ahmed Mubeen et al. 2020), or macro-scale acoustic wave propagation (Fernandes et al. xxxx; Sharma et al. 2025), the integration of modal analysis, frequency–response characterization, and macro-to-micro transmissibility within a unified computational environment represents a significant methodological advance. The multi-scale coupling strategy, connecting tissue-level acoustic fields to intracellular strain amplification, provides a mechanistically grounded basis for frequency selection and highlights disease-dependent differences between healthy and osteoarthritic cartilage. Additionally, the systematic comparison between linear and nonlinear acoustic formulations strengthens the modelling assumptions by demonstrating that, under the investigated pressure ranges, nonlinear propagation effects are negligible.

Experimental evidence increasingly indicates that cartilage degeneration produces measurable alterations in both acoustic and mechanical behaviour. Knee-generated acoustic signals, analysed using multiscale signal-processing and machine-learning methods, have shown potential for distinguishing different stages of cartilage damage and supporting non-invasive OA assessment (Machrowska et al. 2025). In living human knees, intraoperative ultrasonic measurements demonstrated that increasing cartilage degeneration grade was associated with reduced acoustic signal intensity and cartilage thickness, confirming that disease-related structural changes are reflected in the measured ultrasonic response (Nakagawa et al. 2023). Similarly, quantitative high-frequency ultrasound measurements in an ovariectomized rat model identified site-dependent changes in cartilage thickness, surface roughness index, and reflection coefficient following OA-related degeneration (Wang et al. 2016). Ultrasound-based elastography has also been shown to distinguish alterations in cartilage stiffness under experimentally induced OA conditions, demonstrating that acoustic-wave propagation is sensitive to changes in cartilage mechanical properties (Georgas et al. 2024) More broadly, ultrasound can function not only as a diagnostic modality but also as a mechanical stimulus capable of regulating mechanotransductive pathways, supporting the investigation of frequency-dependent acoustic stimulation for musculoskeletal regeneration and therapy (Qin 2025). Collectively, these studies support the biological and mechanical plausibility of using acoustic responses to characterize tissue degeneration and motivate the experimental evaluation of the candidate frequency bands predicted in the present work. Nevertheless, because the cited studies employed different excitation mechanisms, frequencies, experimental models, and outcome measures, they provide contextual support rather than direct validation of the present numerical results.

However, several limitations must be acknowledged. The study relies entirely on computational modelling, and the predicted nuclear strain levels and optimal frequency bands require experimental validation. Material properties, particularly high-frequency viscoelastic and attenuation parameters at the cellular scale, are derived from available literature and may not fully capture in vivo behaviour. Medical phantoms with controlled acoustic and mechanical properties could be explored as a reproducible alternative to biological tissues for preliminary experimental validation of wave propagation and pressure-to-strain transmission (Santos et al. 2025; Mencarelli et al. 2026; Fernandes et al. 2025c). Furthermore, biological mechanotransduction involves complex biochemical feedback mechanisms that are not represented in the present mechanical model. Future work should therefore include controlled experimental measurements, both at the tissue level and at the cellular/nuclear level, to validate the predicted strain magnitudes and frequency-dependent responses. Interspecies differences in bone microarchitecture, density, porosity, and mineralization may affect local acoustic reflection and transmission, particularly near bone–soft tissue interfaces. These differences may influence the absolute magnitude of the localized pressure amplification factor, $|{H}_{pp}|$, although the overall amplification trends are expected to remain representative because the adopted impedance values lie within physiological ranges reported for human tissues. Despite these limitations, the proposed framework establishes a quantitative foundation for designing ultrasound stimulation protocols grounded in cellular-scale mechanics.

Although the macroscopic propagation framework and tissue-specific dissipative parameters were previously calibrated using experimentally measured acoustic transmission through ex vivo biological tissues, the complete knee-specific model presented here has not yet been directly validated using an anatomically representative knee phantom or cadaveric specimen. Directly comparable experimental datasets describing frequency-dependent pressure transmission through the heterogeneous knee joint remain scarce, particularly within the frequency range investigated here. Consequently, the predicted resonance bands should be interpreted as computationally derived candidates intended to guide subsequent experimental studies rather than as clinically validated stimulation frequencies. Future work will focus on knee-specific phantom and ex vivo validation of the predicted acoustic transmission profiles and resonance behaviour.

Articular cartilage was represented as a homogeneous effective medium in the macroscopic model. Although native cartilage exhibits depth-dependent anisotropy, the wavelengths associated with the investigated frequencies, which extend up to 200 kHz, are substantially larger than the characteristic dimensions of its individual structural zones. The homogenized approximation was therefore considered appropriate for evaluating whole-joint acoustic transmission. Nevertheless, zonal variations may affect local pressure amplitudes, attenuation, and phase and should be investigated in future multilayer or anisotropic models.

Several unanswered questions remain and provide clear directions for future research. First, while the model predicts frequency-dependent nuclear strain amplification, the direct relationship between predicted strain magnitudes and specific biological outcomes, such as gene expression, matrix synthesis, or inflammatory modulation, has not yet been experimentally established. The precise mechanotransductive thresholds for chondrocytes under high-frequency acoustic loading remain insufficiently characterized, particularly in osteoarthritic conditions. Additionally, the present framework assumes homogeneous and isotropic tissue properties, whereas cartilage exhibits depth-dependent structure and anisotropy that may influence wave propagation and energy focussing. It would also be worthwhile to adopt the framework developed by Girón and Lelis (Gómez Girón and Rodríguez Lelis 2025), to model chondrocytes, rather than representing the entire domain as a multi-layer homogeneous continuum. In particular, the cytoskeleton could be explicitly discretized using truss elements, allowing the structural contribution of the filament network to be captured more realistically. The resulting model could then be validated against experimental data available in the literature, thereby improving the accuracy and physical representativeness of the RVE formulation.

The use of infinitesimal-strain linear elasticity does not imply that the predicted cellular deformation is biologically insignificant. It indicates that the strain amplitude is assumed to remain sufficiently small for a linear constitutive approximation. Mechanotransduction may occur under small cyclic deformation through repeated loading of the cell membrane, cytoskeleton, nucleus, and extracellular matrix attachments, particularly when excitation occurs near a mechanically responsive mode. Under these conditions, modal amplification may increase local cytoplasmic or nuclear strains relative to off-resonance stimulation, even when the externally imposed acoustic displacement is small. A strain range of approximately 1–10% has been reported as a useful biological reference for mechanically induced cellular and nuclear responses; however, this should not be interpreted as a universal threshold, since the response also depends on frequency, exposure duration, number of cycles, cell type, and loading configuration. The present model therefore identifies frequencies that are mechanically favourable for deformation but does not directly predict downstream biochemical signalling.

Periodic boundary conditions were applied to the microscale RVE to represent a chondrocyte embedded within a continuous tissue environment and to minimize artificial boundary effects associated with isolated finite domains. This approximation is appropriate for evaluating the controlled influence of selected geometrical parameters on the local chondrocyte–PCM–ECM response. Chondrocyte spacing was explicitly included as a parametric variable, allowing the influence of cellular spatial periodicity on the predicted resonant behaviour to be assessed. Within the investigated range, the dominant changes in natural frequency were primarily associated with chondrocyte radius and PCM thickness, whereas chondrocyte spacing produced comparatively smaller variations.

Nevertheless, the periodic RVE should be interpreted as an idealized microscale representation rather than a complete description of severely degraded OA cartilage. Advanced OA tissue may contain irregular chondrocyte clusters, disrupted PCM regions, fissures, and spatially heterogeneous matrix properties that are not fully represented by a single periodic unit cell. Consequently, the present model does not explicitly resolve nonperiodic cluster-to-cluster stress interactions. Future studies should incorporate stochastic or image-based multi-cell RVEs to determine how OA-induced clustering and local matrix heterogeneity affect cellular-scale acoustic strain transfer.

The long-term biological consequences of repeated or chronic acoustic stimulation, including potential cumulative effects or adaptation mechanisms, also remain unexplored. Future work should integrate experimental validation at multiple scales, incorporate more detailed tissue heterogeneity, and potentially couple mechanical models with biochemical signalling pathways to bridge mechanics and cell biology. The broader implications of this work extend beyond cartilage stimulation: the proposed multi-scale strategy could inform the design of targeted ultrasound therapies for other mechanosensitive tissues, enabling patient-specific optimization of stimulation parameters grounded in cellular-scale mechanics rather than empirical frequency selection.

## Conclusion

This study addressed the need for a mechanistic understanding of how externally applied acoustic waves used in cartilage stimulation translate into biologically relevant deformation at the level of the chondrocyte nucleus. Current ultrasound protocols are largely empirical and do not explicitly account for multi-scale mechanical transmission from the joint to the cell. To resolve this gap, a patient-specific multi-scale computational framework was developed, coupling macro-scale acoustic wave propagation in the knee with micro-scale cellular mechanics through a representative volume element approach. The results demonstrated that nuclear strain amplification occurs within defined frequency bands rather than at a single discrete frequency, with the spheroidal s_3_ mode playing a dominant role in nuclear deformation. Osteoarthritic alterations were shown to shift the optimal frequency band towards lower frequencies and reduce higher-frequency sensitivity (Healthy: 50–170 kHz, OA: 30–85 kHz). Despite anatomical variability across patients, consistent transmissibility trends were observed, and time-explicit simulations confirmed that, at the applied pressure levels, nonlinear acoustic effects are negligible. It should nevertheless be acknowledged that the proposed framework relies on several simplifying assumptions regarding the geometrical representation, material properties, and mechanical behaviour of the biological tissues. These assumptions were necessary to reduce model complexity and maintain computational feasibility, but they may limit the extent to which the numerical predictions reproduce the full physiological response of the knee joint. Further experimental validation is therefore required to confirm the accuracy of the predicted acoustic fields, modal behaviour, and tissue-level mechanical responses. The key takeaway is that effective cartilage stimulation should be frequency-band targeted and disease-sensitive, and that linear acoustic modelling is sufficient within mechanotransductive strain ranges relevant for therapy. The proposed framework provides a quantitative basis for rational selection of ultrasound parameters grounded in cellular-scale mechanics rather than empirical tuning.

## Figures and Tables

**Fig. 25:**
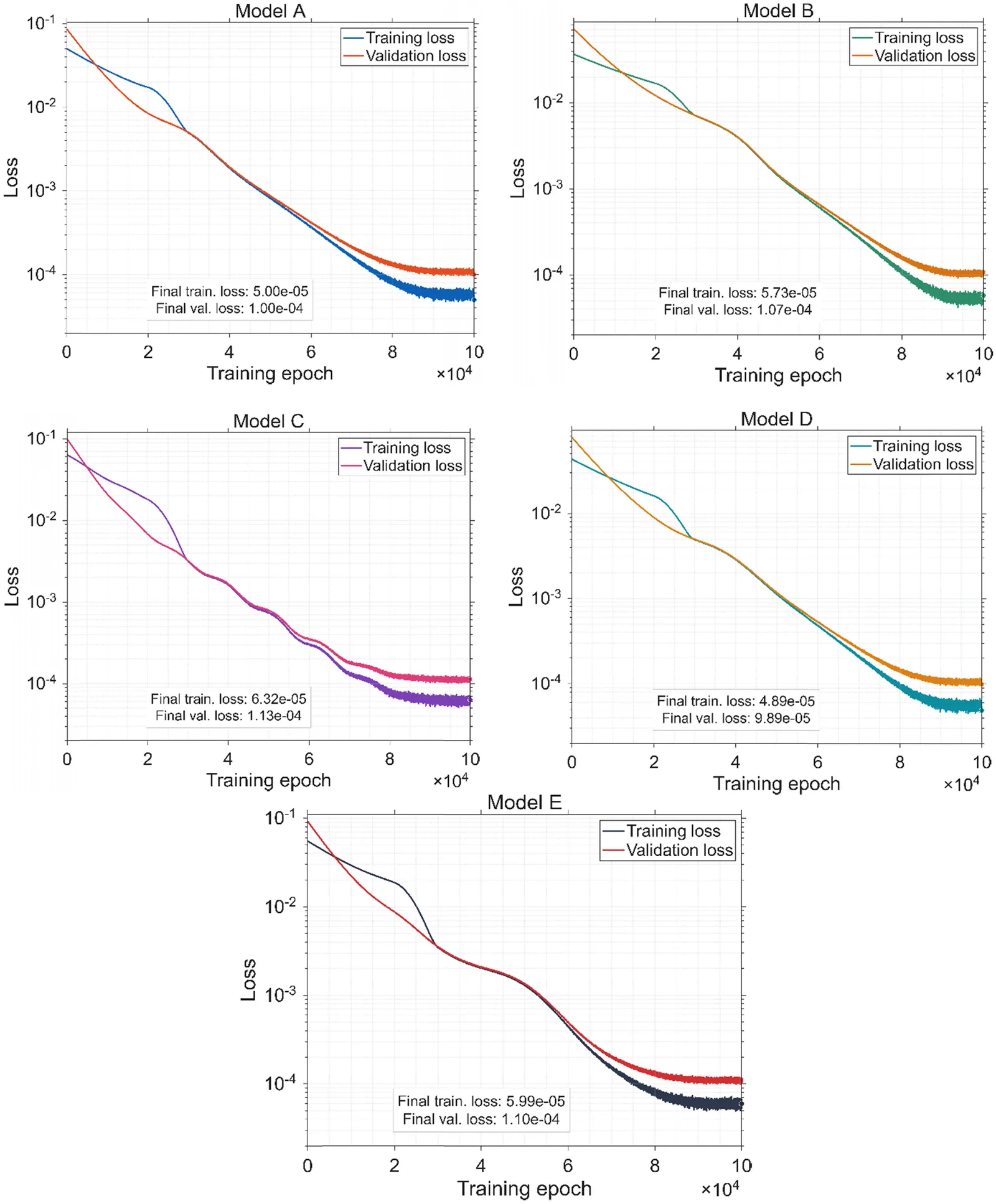
raining and validation loss histories for DNN surrogate Models A–E over 100,000 epochs

## Data Availability

The data used for the patient's knee geometry was obtained from the Open Knee(s): Virtual Biomechanical Representations of the Knee Joint, available at https://simtk.org/projects/openknee

## Appendix A Deep neural network surrogate models validation and training

DNN surrogate models were developed to approximate the relationship between the geometrical parameters of the chondrocyte microenvironment and the corresponding modal frequencies obtained from the finite-element simulations. The surrogate-modelling approach enabled rapid exploration of the multidimensional parameter space while substantially reducing the computational cost associated with repeated finite-element evaluations. The networks were trained using simulation-generated datasets and were applied only within the parameter ranges represented during training.

In total five models were explored, as is described in Table

**Table 5:** Summary of the five deep neural network surrogate models, including the corresponding finite-element analysis, input geometrical parameters, predicted modal-frequency or frequency–response outputs, and parameter-space sampling method

Model	Description	Input	Output	Sampling method
A	Modal analysis of healthy chondrocytes without ECM	${\mathbf{x}} = [r_{{\mathrm{c}}} , {t}_{\mathrm{P}\mathrm{C}\mathrm{M}}]$	$\mathbf{y}=\left[{\omega}_{{s}_{1}},{\hspace{0.17em}}{\omega}_{{s}_{2}},{\hspace{0.17em}}{\omega}_{{s}_{3}},{\hspace{0.17em}}{\omega}_{{s}_{4}},{\omega}_{{t}_{1}},{\hspace{0.17em}}{\omega}_{{t}_{2}}\right]$	Uniform
B	Modal analysis of healthy chondrocytes with ECM	$\mathbf{x}=[{r}_{\mathrm{c}}, {t}_{\mathrm{P}\mathrm{C}\mathrm{M}}]$	$\mathbf{y}=\left[{\omega}_{{s}_{1}},{\hspace{0.17em}}{\omega}_{{s}_{2}},{\hspace{0.17em}}{\omega}_{{s}_{3}},{\hspace{0.17em}}{\omega}_{{s}_{4}},{\omega}_{{t}_{1}},{\hspace{0.17em}}{\omega}_{{t}_{2}}\right]$	Uniform
C	Modal analysis of OA chondrocytes without ECM	$\mathbf{x}=[{r}_{\mathrm{c}}, {t}_{\mathrm{P}\mathrm{C}\mathrm{M}}]$	$\mathbf{y}=\left[{\omega}_{{s}_{1}},{\hspace{0.17em}}{\omega}_{{s}_{2}},{\hspace{0.17em}}{\omega}_{{s}_{3}},{\hspace{0.17em}}{\omega}_{{s}_{4}},{\omega}_{{t}_{1}},{\hspace{0.17em}}{\omega}_{{t}_{2}}\right]$	Uniform
D	FRF of healthy chondrocytes with ECM	$\mathbf{x}=[{r}_{\mathrm{c}}, {t}_{\mathrm{P}\mathrm{C}\mathrm{M}}]$	$\mathbf{y}=\left[{\varepsilon}_{{s}_{1}},{\hspace{0.17em}}{\varepsilon}_{{s}_{2}},{\hspace{0.17em}}{\varepsilon}_{{s}_{3}},{\hspace{0.17em}}{\varepsilon}_{{s}_{4}},{\hspace{0.17em}}{\varepsilon}_{{t}_{1}},{\hspace{0.17em}}{\varepsilon}_{{t}_{2}}\right]$	Uniform
E	FRF of OA chondrocytes with ECM	$\mathbf{x}=[{r}_{\mathrm{c}}, {t}_{\mathrm{P}\mathrm{C}\mathrm{M}}]$	$\mathbf{y}=\left[{\varepsilon}_{{s}_{1}},{\hspace{0.17em}}{\varepsilon}_{{s}_{2}},{\hspace{0.17em}}{\varepsilon}_{{s}_{3}},{\hspace{0.17em}}{\varepsilon}_{{s}_{4}},{\hspace{0.17em}}{\varepsilon}_{{t}_{1}},{\hspace{0.17em}}{\varepsilon}_{{t}_{2}}\right]$	Uniform

5.

For each model, 1000 simulations were conducted using a uniform parameter-space sampling strategy. Cases that failed to converge or were otherwise unsuccessful were excluded from the dataset, together with repeated parameter combinations.

Because the ordering of the eigenfrequencies changed for some parameter combinations, the modes were classified according to their deformation patterns rather than their numerical position in the eigensolution. Symmetric and torsional modes were identified from the spatial displacement fields and tracked throughout the parameter space to preserve a physically consistent correspondence between the network outputs and the underlying vibration modes, using Eq. (13).

Both input and output variables were normalized prior to network training. Geometrical input parameters were expressed in micrometres (*µ*m), whereas the predicted modal frequencies were reported in kilohertz (kHz). The dataset was randomly partitioned into training, validation, and independent testing subsets, comprising 80%, 10%, and 10% of the available observations, respectively, using a fixed random seed to ensure reproducibility.

The DNN architecture was defined as $\left[2,256,128,64,32,6\right]$, comprising two input neurons associated with the selected geometrical parameters, an inverted pyramid scheme of four fully connected hidden layers containing 256, 128, 64, and 32 neurons, respectively, and a six-neuron linear output layer. Gaussian Error Linear Unit (GELU) activation functions were applied throughout the hidden layers. Depending on the modelling objective, the output layer simultaneously predicted either the frequencies of the six vibration modes or their corresponding frequency–response amplitudes.

Training occurred over 100,000 epochs, with a learning rate of 10^–6^, weight decay of 10^–5^, batch size of 256. Predictive performance and convergence were monitored through the training and validation loss histories, with the optimization process attaining a validation loss of approximately 10^–4^, as can be observed in Fig. 25. 

All five DNN models exhibited a consistent reduction in training and validation losses over 100,000 epochs, followed by stabilization during the final training stage. Final training losses ranged from $4.89\cdot {10}^{-5}$ to $6.32\cdot {10}^{-5}$, while validation losses varied between $9.89\cdot {10}^{-5}$ and $1.13\cdot {10}^{-4}$. Model D achieved the lowest validation loss, whereas Model C presented the highest value.

The comparable convergence patterns and limited separation between the training and validation curves indicate stable optimization and satisfactory generalization across the five modelling conditions. The slightly higher validation losses suggest a modest generalization gap, although no progressive divergence indicative of substantial overfitting was observed. The similar final values further demonstrate that the adopted architecture and training configuration provided consistent predictive performance for both the modal and frequency–response models.

The resulting DNNs should be interpreted as computational surrogates of the finite-element models rather than independent physical models. Their predictions therefore inherit the assumptions associated with the material properties, geometrical idealizations, constitutive descriptions, and boundary conditions adopted in the numerical simulations. Furthermore, model use was restricted to the parameter intervals represented in the training dataset, because predictive accuracy outside this domain was not established. Particular caution is required near modal-transition regions, where changes in mode ordering or deformation morphology may reduce the smoothness of the learned input–output relationship.

## Appendix B:Transfer function magnitude from transducer to cartilage for 2D simulations

See Figs.26, 27, 28, 29, 30, 31, 32 and 33.

## Appendix C:Transfer function magnitude from transducer to cartilage for 3D simulations

See Figs. 34, 35, 36, 37, 38, 39, 40 and 41.

## Abbreviations

ASI: Acoustic-structure interaction
CFL: Courant–Friedrichs–Lewy
DG: Discontinuous Galerkin
DNN: Deep neural network
ECM: Extracellular matrix
FLIPUS: Focussed low-intensity pulsed ultrasound
FRF: Frequency response function
LIPUS: Low-intensity pulsed ultrasound
OA: Osteoarthritis
PCM: Pericellular matrix
RVE: Representative volume element
